# A photometric stereo-based 3D imaging system using computer vision and deep learning for tracking plant growth

**DOI:** 10.1093/gigascience/giz056

**Published:** 2019-05-25

**Authors:** Gytis Bernotas, Livia C T Scorza, Mark F Hansen, Ian J Hales, Karen J Halliday, Lyndon N Smith, Melvyn L Smith, Alistair J McCormick

**Affiliations:** 1 Centre for Machine Vision, Bristol Robotics Laboratory, University of the West of England, T block, Frenchay Campus, Coldharbour Lane, Bristol BS16 1QY, UK; 2 SynthSys & Institute of Molecular Plant Sciences, School of Biological Sciences, University of Edinburgh, The King's Buildings, Edinburgh EH9 3BF, UK

**Keywords:** Arabidopsis thaliana, leaf angle, segmentation, machine learning, near-infrared LEDs, photomorphogenesis, thermomorphogenesis

## Abstract

**Background:**

Tracking and predicting the growth performance of plants in different environments is critical for predicting the impact of global climate change. Automated approaches for image capture and analysis have allowed for substantial increases in the throughput of quantitative growth trait measurements compared with manual assessments. Recent work has focused on adopting computer vision and machine learning approaches to improve the accuracy of automated plant phenotyping. Here we present PS-Plant, a low-cost and portable 3D plant phenotyping platform based on an imaging technique novel to plant phenotyping called photometric stereo (PS).

**Results:**

We calibrated PS-Plant to track the model plant *Arabidopsis thaliana* throughout the day-night (diel) cycle and investigated growth architecture under a variety of conditions to illustrate the dramatic effect of the environment on plant phenotype. We developed bespoke computer vision algorithms and assessed available deep neural network architectures to automate the segmentation of rosettes and individual leaves, and extract basic and more advanced traits from PS-derived data, including the tracking of 3D plant growth and diel leaf hyponastic movement. Furthermore, we have produced the first PS training data set, which includes 221 manually annotated *Arabidopsis* rosettes that were used for training and data analysis (1,768 images in total). A full protocol is provided, including all software components and an additional test data set.

**Conclusions:**

PS-Plant is a powerful new phenotyping tool for plant research that provides robust data at high temporal and spatial resolutions. The system is well-suited for small- and large-scale research and will help to accelerate bridging of the phenotype-to-genotype gap.

## Introduction

Quantitative and accurate methods are required to aid strategies for predicting plant growth performances in our changeable natural environments. Such tools are critical for calibrating predictive models in the face of a changing global climate and our growing global population [1–6]. Computer vision is an evolving technology that is helping to drive advances in plant phenotyping in both fundamental research and agriculture [7–10]. Reflecting its considerable promise, effort has been directed toward automated ground vehicles [11, 12], satellite [13], drone [14], and gantry-style platform imaging of field plants [15], and automated phenotyping of greenhouse- [16, 17] and laboratory-grown plants (the challenges are different for field and indoor phenotyping) [18, 19]. While there have been significant advances, problems associated with high cost, automated data capture, large data sets, and variable visual and temporal resolutions have created barriers to the uptake of these technologies. These challenges are currently being addressed in the next generation of plant phenotyping tools.

Above ground growth is a strong indicator of plant yield, and therefore 3D imaging of vegetative growth is a very active area of phenotyping research [20–25]. A number of excellent 2D imaging systems have been developed [26–28]; however, while they represent a qualitative improvement on manual data capture, they have limited capacity to resolve plant architecture at high resolution. For example, leaf area measurements are affected by blade curvature, leaf angle, and movement, making accurate estimations of plant growth challenging using 2D methods [9, 29]. Several 3D imaging methods have been developed that overcome some of the limitations of 2 dimensions. These can be classified as passive and active 3D imaging approaches and are briefly outlined below.

Passive 3D imaging approaches capture plant architecture without introducing new energy (e.g., light) into the environment [30]. Methods and technologies using this approach include multi-view stereo [31, 32], of which the most common is binocular stereo [33, 34], structure from motion [35], light-field (plenoptic) cameras [36], and space-carving approaches [37]. Passive approaches that use ≥2 sensors or have moving parts (e.g., robot arm or gantry systems) often encounter difficulties in identifying and aligning the same points in different images (i.e., the so-called correspondence problem), which can result in imprecise reconstruction of 3D shapes [38]. Plant leaves and canopies can be particularly challenging because they often represent large homogenous areas with little salient texture. Imprecise 3D reconstructions can be smoothed but at the expense of plant surface detail [39]. Space carving overcomes the correspondence problem but requires many different views of an object and may still fail to reconstruct crowded areas (e.g., overlapping leaves) [37]. To our knowledge, only light-field cameras have been utilized successfully for capturing 3D plant growth throughout the diel (day-night) cycle [36, 40]. However, light-field systems rely on expensive camera technology to capture high-resolution data and, like other passive approaches, require consistent and favourable lighting conditions.

Active 3D imaging approaches emit energy (e.g., light), which can overcome several problems associated with passive approaches. Structured light [41] and laser scanners [42–44] are active technologies that rely on triangulation to determine the point locations in a 3D space. Both methods can provide high-quality 3D reconstructions of plant canopy architecture, but structured light approaches require very accurate correspondence between images while laser scanners can be slow and can potentially heat or even damage plants at high frequencies [45]. Furthermore, triangulation techniques are susceptible to occlusions (e.g., other objects in the environment or leaf overlap) that can reduce data quality. Time-of-flight cameras (e.g., light detection and ranging [LiDAR]) comprise another active 3D imaging approach that determines the distance of a point directly from the time delay between an emitted light pulse and its reflection. However, the resolution of time-of-flight cameras is still relatively low, which has tended to limit their use to imaging larger plants [46, 47]. Although both passive and active 3D imaging approaches can significantly improve the accuracy of plant growth measurements and even expand on the architectural traits available to capture compared with 2D imaging, existing 3D imaging techniques still lack in several crucial areas such as speed, availability, portability, spatial resolution, and cost [25].

**Figure 1:**
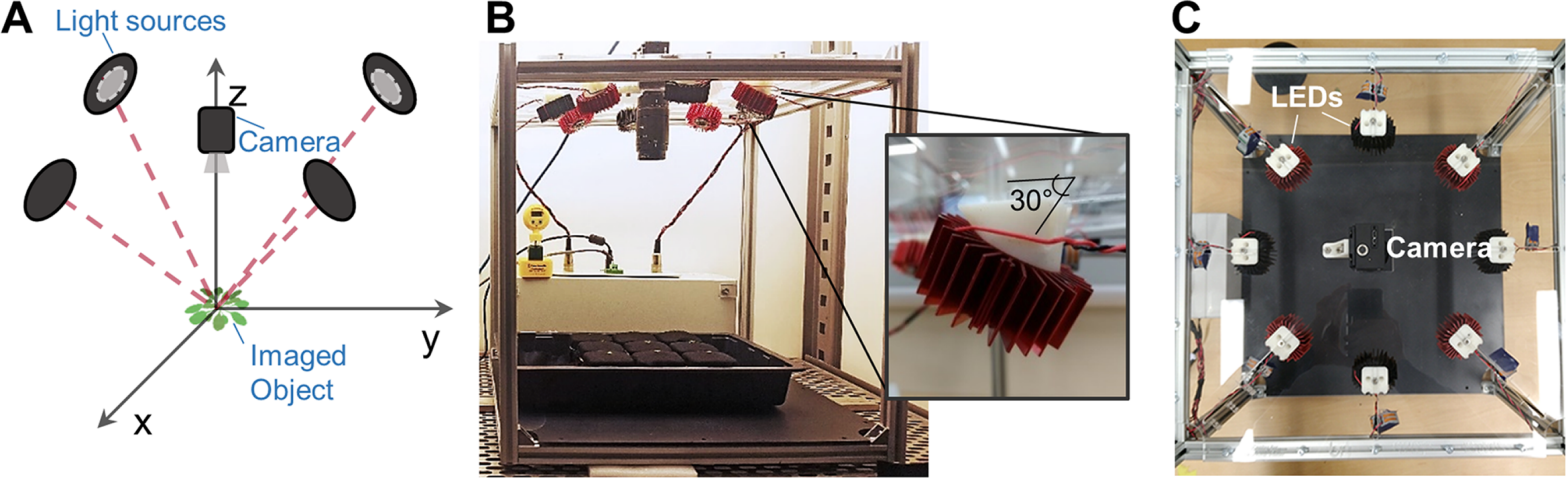
Capturing dynamic plant growth traits using photometric stereo imaging. (**A**) PS comprises a circular arrangement of NIR LEDs with a central camera positioned above the plant(s). Red dashed lines show the direction of light vectors. (**B, C**) Assembled PS-Plant system shown from side and top views. Each LED is attached to a dedicated heatsink and angled at 30° using a custom 3D-printed bracket to minimize the light distribution across the field of view. Both the camera and light sources are stationary.

Photometric stereo (PS) is an active imaging technique that is low-cost and can achieve high image resolutions and fast capture speeds [48]. This approach has been applied only recently to plant phenotyping and has shown significant promise [49]. PS relies on a set of images of an object captured under controlled, varied, and directional illumination (Fig. 1; Supplementary Information S1). The obtained images are then used to generate a dense surface normal (SN) map of matching resolution, where each pixel represents a normal vector's components (i.e., the orientation in 3 cardinal directions—*x, y*, and *z*) that allows the overall orientation of the object to be determined. Prior work has shown that plant leaf SN data acquired by PS can be captured at high resolutions (4.1 megapixels) and thus has significant advantages in encoding complex 3D morphology to aid challenging automated recognition and quantification tasks, such as the extraction of plant growth data [49, 50].

Machine learning is now emerging as a promising field to transform the automation of trait extractions from plant image data sets [51, 52]. Work in the model plant *Arabidopsis thaliana* (hereafter *Arabidopsis*) has revealed much about the molecular processes underlying the relationship between leaf area, biomass, and yield [53], and several methods have been developed for automating data extraction from *Arabidopsis* images [54–56]. Recently, significant advances have been made in the development of artificial neural networks (NNs) for automated segmentation of the rosette and individual leaves, and leaf counting using 2D image data [57–59]. However, the performance of NN approaches for leaf segmentation, for example, are still limited by a need for large annotated data sets for training because models trained with small-scale databases typically generalize weakly. To our knowledge, currently there are no NN models optimized for leaf segmentation using 3D data. A subsequent challenge is accurate object tracking to enable segmented leaves to be tracked across different time points of a data set [60, 61].

Here we present a novel, low-cost imaging system called PS-Plant that for the first time utilizes PS for monitoring the growth and development of *Arabidopsis* in 3 dimensions. We compared the accuracy of 3D vs 2D data from PS-Plant for estimating leaf area, angle, and rosette growth against ground truth measurements and showed comparable results to the state-of-the-art 3D light-field camera and laser scanning systems [36, 43, 44]. To demonstrate the versatility of PS-Plant, we analysed growth under a matrix of different conditions that illustrate the dramatic effect of the environment on the 3D phenotype of a wild-type *Arabidopsis* plant. Furthermore, we showed that 3D data from PS-Plant can be used to train NN models for automated leaf segmentation of a growing rosette, as an important first step in extracting plant features. Finally, we demonstrated that utilization of machine learning for leaf segmentation and PS data can be combined to extract useful growth traits related to dynamic leaf movement and rosette development.

## Results

### Photometric stereo imaging using PS-Plant provides accurate spatial data for *Arabidopsis* plants

PS-Plant consists of a machine vision camera surrounded by 4 or 8 near-infrared (NIR) light-emitting diodes (LEDs) and a bespoke LED controller that allows rapid switching of the LEDs for high temporal data acquisition (Fig. 1; Supplementary Data S1). PS-Plant can acquire up to 40 2D images per second at a spatial resolution of 2,048 × 2,048 pixels. The acquisition process takes 125–225 milliseconds per set of PS images, followed by ∼5 s to process the 2D images to compute SN map estimations and 3D surface integration (Supplementary Information S1 and S2). An NIR filter positioned in front of the lens provides consistent contrast and brightness for images captured throughout the diel cycle. The camera provides a 17 × 17 cm field of view that allows simultaneous tracking of ≤9 *Arabidopsis* plants in 5 × 5 cm pots. Growth data sets for individual plants were extracted from each master image experiment data set using Python-based graphical user interface (GUI) software. Overall, PS-Plant is portable and lightweight (∼7 kg without a personal computer) and could be adjusted to fit in different growth environments including growth cabinets or greenhouse environments. At the time of manufacture, the total cost for PS-Plant was approximately US$3200.

**Figure 2:**
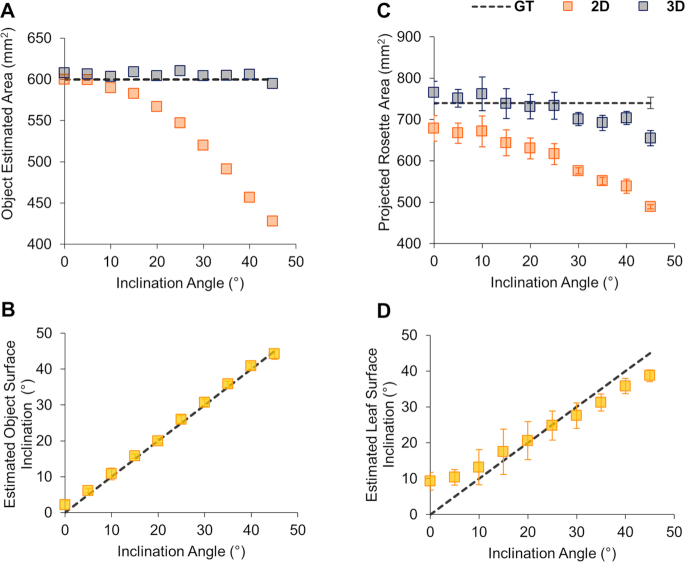
Evaluating the accuracy of PS-Plant with 2D and 3D data. (**A, B**) The estimated area and inclination angle of a flat, matte object (600 mm^2^) from 0° to 45° at 5° intervals. Each data point represents the average of 30 randomly selected regional patches of varying size (35–600 mm^2^). (**C, D**) The area of 3 similarly sized *Arabidopsis* whole rosettes (750 ± 13.5 mm^2^) and leaf inclination angles were estimated from 0° to 45° at 5° intervals. The dashed black lines indicate ground truth (GT) measurements. Error bars represent SD of the means.

A key assumption in PS is that the surface of the imaged object should exhibit Lambertian reflectance (i.e., it reflects light equally in all directions, while the reflected intensity diminishes according to Lambert's cosine law) (Supplementary Information S1) [48]. As the reflectance of the object deviates from the Lambertian model, the subsequent estimation error increases accordingly. To verify whether PS-Plant could accurately estimate the total area and angle of an object, we initially used rectangular flat pieces of acrylic of known area (600 mm^2^) covered in white matte paper, which achieved a close approximation of Lambertian reflectance [62], and imaged with a black matte background to facilitate image segmentation [63]. The acrylic objects were placed on laser-cut wedges to allow imaging at a range of known angles (Fig. 2A). The projected areas were estimated using 2D and 3D data obtained from PS-Plant. The 3D data enabled us to estimate the object inclination angles, which were compared with the ground truth (Fig. 2B). Using 3D data, the area was estimated accurately up to 45° with a mean relative error (MRE) of 1.0% (see Supplementary Information S3 for formulas). In contrast, estimates based on 2D data became inaccurate at inclinations >10°, with an MRE of 10.3% when all angles were considered. Angle estimations consistently matched the known angle for all inclinations tested with a mean absolute error of 0.89°. These results highlighted the accuracy of PS-Plant in estimating the angle and area of a flat object in 3D space.

Next, we investigated *Arabidopsis* rosettes in PS-Plant and observed that *Arabidopsis* leaves exhibited near-Lambertian reflectance under NIR light (Supplementary Information S1). We hypothesized that longer wavelengths penetrate deeper into the leaf and are then typically scattered, rather than specularly reflected at the leaf surface [64, 65]. Similarly to the object area and angle estimation experiment, we imaged *Arabidopsis* rosettes inclined from 0° to 45° using a rotary inclination table and compared the estimated areas using 2D and 3D data with ground truth measurements of the imaged rosettes (Fig. 2C). Even without inclination (i.e., at 0°), estimates based on 3D data were more accurate than those from 2D data, indicating that the former was more capable of approximating areas for complex objects that include a degree of surface topographic relief (e.g., an *Arabidopsis* rosette). 3D data continued to outperform 2D data at increased inclinations with an MRE of 4.5% and 18.1% for 3D and 2D estimations, respectively. The accuracy of 3D estimations did decrease at angles >30° as a result of the increase in leaf (self-) occlusion that occurred when the whole rosette was inclined (Supplementary Information S4). When the accuracy of angle estimations was tested with selected individual leaves from the *Arabidopsis* rosettes (Fig. 2D), PS-Plant achieved a mean absolute error of 3.8° for leaf angle estimations. We observed that the estimated and known leaf inclination angle correlated in the mid-range (10–30°) but less so at lower and higher angles. This was likely due to the natural curvature of *Arabidopsis* leaves compared to a flat surface, as *Arabidopsis* leaf blades typically have a convex shape when observed from above. Therefore, when the leaves were not inclined (i.e., at 0°), the estimated angles were still >0° because they were calculated from the varying SN values across each leaf blade surface.

### PS-Plant enables accurate 3D reconstructions of growing *Arabidopsis* rosettes

**Figure 3:**
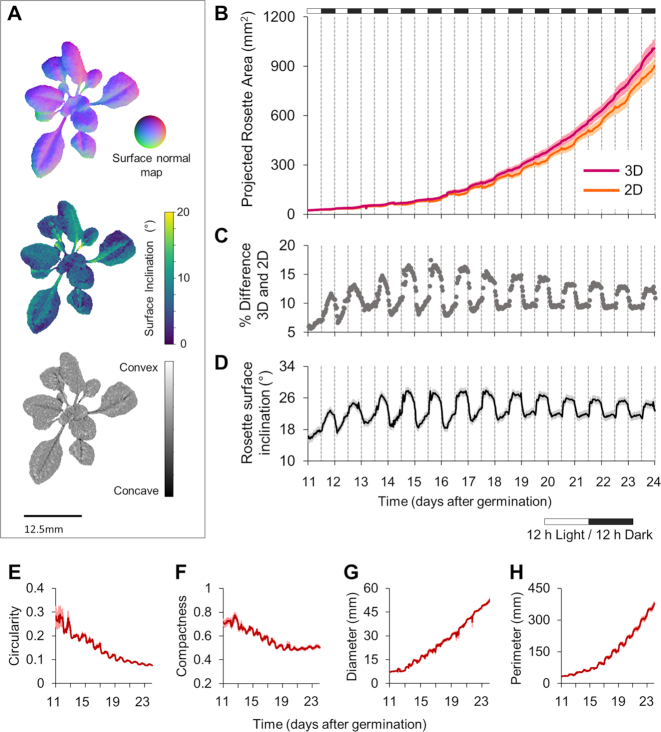
Data outputs of PS-Plant for *Arabidopsis*. (**A**) Surface normal map (top) rendered for a wild-type *Arabidopsis* rosette used to derive models for surface inclination (middle) and convexity (bottom). (**B**) Projected rosette area estimates captured for wild-type plants under standard growth conditions (22°C, 150 µmol photons m^−2^ s^−1^, 12:12 h light:dark) for 2D and 3D data from the mean ± SE values of 13 biological replicates. (**C**) Percentage difference between 2D and 3D estimations. (**D**) Estimated rosette mean inclination angles across the rosette surface. (**E−H**) Circularity, compactness, diameter, and perimeter estimates derived from 2D data.

Following validation, we assessed the accuracy and consistency of PS-Plant in monitoring plant growth and mean rosette inclination over time (Fig. 3). PS-Plant captured both 2D and 3D data for *Arabidopsis* plants for 12 days, starting from 11 days after germination (DAG) in standard growth conditions (22°C, 150 µmol photons m^−2^ s^−1^, 12:12 h light:dark). The automated image capture program resulted in an SN map produced for each plant every 30 minutes that was used to characterize rosette surface curvature (Fig. 3A) as described in Supplementary Information S1. Furthermore, SN data could be used to derive rosette surface inclination angles and concavity/convexity values. Such information can be used, for example, in leaf developmental analysis to evaluate perturbances in normal leaf abaxial/adaxial expansion [66, 67].

Both 2D and 3D data sets produced exponential growth curves for projected rosette area (PRA) that were typical for *Arabidopsis* growth (Fig. 3B). However, 2D data consistently underestimated PRA and showed erroneous reductions in area estimates consistent with rhythmic nastic leaf movements (Fig. 3C and D; Supplementary Data S2). In contrast, 3D data accounted for leaf curvature and movement (Supplementary Information S1), such that PRA increased more smoothly over the time course of the experiment. The small decreases observed for PRA from 3D data were associated with self-occlusion at high leaf inclination angles (as in Fig. 2). A number of studies have shown that growing *Arabidopsis* leaves exhibit rhythmic movement that is controlled by the circadian oscillator [68–71]. PS-Plant estimations of rosette surface inclination (i.e., the total inclination of all rosette leaf blades and petioles) is able to accurately record this rhythmicity, which in our 12:12 h light:dark conditions achieved an amplitude peak at 4–6 h after dusk (Fig. 3D) (calculated using BioDare2; see Materials and Methods). Interestingly, our data showed that leaf rhythmicity appears to be anticipatory up to 16 DAG, after which it was strictly diurnal. Because older plants have a higher proportion of mature leaves that are no longer elongating, our data suggest that these leaves still exhibit rhythmic movements but they are driven by the daily light-dark cycle rather than the circadian oscillator. These data highlight the capability of PS-Plant not only to provide accurate area estimates but to capture leaf movement rhythms that are regulated by the circadian clock and the prevailing photoperiod.

Rosette architectural parameters derived from 2D data were also obtained from PS-Plant, including circularity (or stockiness), compactness, diameter, and perimeter (Fig. 3E-H) [36, 72, 54]. These data showed, for example, an increase in perimeter and diameter that was consistent with plant growth, and a decrease in compactness, which was associated with elongation of leaf petioles as the rosette developed.

### PS-Plant reveals 3D growth traits for *Arabidopsis* plant grown in different environments

**Figure 4:**
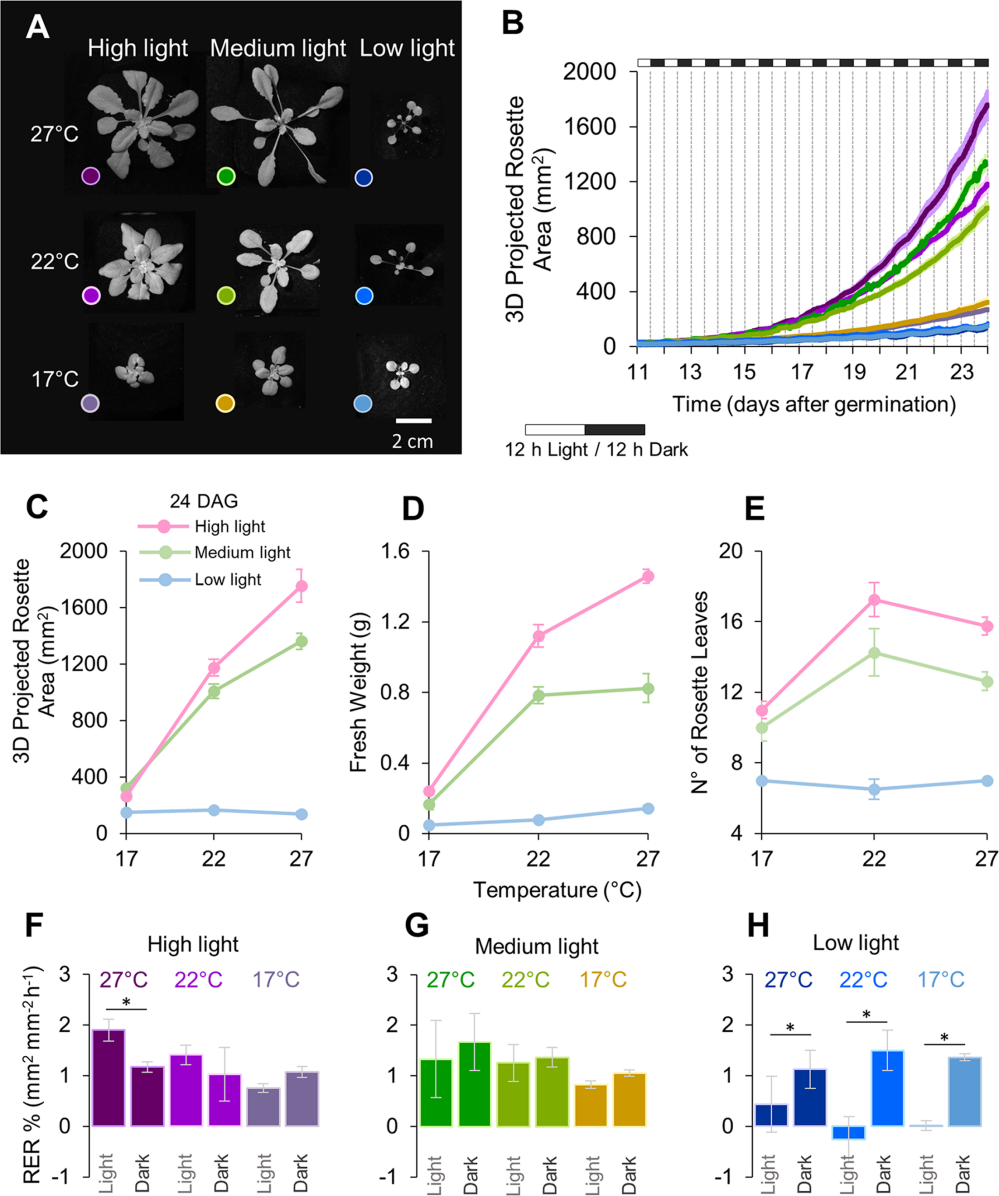
PS-Plant shows that *Arabidopsis* plants grown under different conditions show differences in growth architecture. (**A**) Wild-type *Arabidopsis* plants (24 DAG) following growth under 9 different light and temperature conditions. (**B**) Estimated 3D projected rosette area growth of rosettes grown under the different environments. (**C–E**) Estimated 3D projected area, fresh weights, and leaf count for rosettes at 24 DAG. (**F–H**) The average relative expansion rate (RER) during light and dark periods for each growth condition (calculated from 15–18 DAG with a 4-hr sliding window). Values represent the mean ± SE values of ≥3 biological replicates. Asterisks indicate significant differences between light and dark values for each condition based on Student's *t*-test (*P* < 0.05). The colour legends in A are applicable to B, and F–H.

We next wanted to establish whether PS-Plant could capture alterations in growth plasticity induced by changes in the external light and temperature environment. Low levels of photosynthetic active radiation are known to elicit a shade avoidance response, where plants exhibit elongated stems and petioles, increased hyponasty, and smaller and fewer leaves [73–75]. As high temperatures to some extent target the same molecular pathways, heat also elicits a shade avoidance –type response [76, 77]. These studies illustrate that the growth strategy adopted by the plant is strongly dependent on the surrounding light environment and the ambient temperature. To capture these morphological changes we tracked *Arabidopsis* plants under 9 conditions that differed in temperature (17°C [low temperature, LT], 22°C [medium temperature, MT], and 27°C [high temperature, HT]) and light intensity (40 [low light, LL], 150 [medium light, ML], and 300 µmol photons m^−2^ s^−1^ [high light, HL]) (Fig. 4A; Supplementary Fig. S1; Data S3).

Plants grown in LL had small leaves, recorded as low PRA, which was comparable in plants grown at different temperatures. Increases in light levels led to a concomitant increase in PRA; however, at light intensities of >150 µmol m^−2^ s^−1^ the PRA was strictly temperature-dependent, with the highest PRA achieved at the highest light and temperature levels (Fig. 4B). The observed differences in PRA were reasonably consistent with overall biomass accumulation at 24 DAG (Fig. 4C and D). Notably, in ML plants a shift from 17°C to 22°C led to an increase in biomass, while a shift from 22°C to 27°C did not. Although we have not measured leaf thickness, previous work has shown that plants grown in high temperatures tend to have thinner leaves and a higher specific leaf area (the ratio of leaf area to dry mass) [78, 79], which could explain the increase in area from 22°C to 27°C but no increase in biomass. HL and ML plants produced more leaves at 22°C than at 17°C, signifying a larger investment in vegetative growth. Growth at 27°C induced flowering in HL and ML plants, and so their final leaf number was slightly lower than at 22°C (Fig. 4E).

Together, these results could be explained by the thermodynamic relationship between the dark reactions (e.g., ribulose bisphosphate carboxylase/oxygenase [rubisco] activity and the Calvin cycle) and light reactions of photosynthesis. The assimilation rate of CO_2_ by rubisco is temperature-dependent, such that increased temperatures (up to ∼30°C) typically correlate with increased CO_2_ assimilation in C3 plants grown under non-limiting light conditions [80–82]. These photochemical processes most likely underlie the light- and temperature-dependent changes in PRA and investment in leaf biomass production. Contrasting with this, in LL the supply of adenosine triphosphate and nicotinamide adenine dinucleotide phosphate to the Calvin cycle by the light reactions may have constrained CO_2_ uptake, and thus growth rates were not increased by higher temperatures.

PS-Plant also captured differences in petiole length. Analysis of the ML and HL specimens illustrated that increased temperature stimulated petiole elongation in these plants. This is evident in PS-Plant measurements of plant compactness. However, these data also show that HL plants were generally more compact than ML (Supplementary Fig. S2) and that temperature-mediated differences in compactness were less evident in plants grown in HL. This indicates that plants tend to invest more in leaf expansion compared to petiole elongation under higher light intensities.

We then compared the relative expansion rate (RER) based on 3D PRA data for different light-temperature conditions over the diel cycle (Fig. 4F–H). RER data for *Arabidopsis* vary between different studies but generally have comparable rates within light and dark periods for wild-type plants grown under standard growth conditions [9, 36, 43, 83]. In the present study, RER in the dark period was not significantly different across all growth conditions tested (as determined by 1-way analysis of variance [ANOVA] [*P* < 0.05], followed by Tukey's honest significant difference [HSD] tests). This was not unexpected because the rate of leaf starch turnover during the night is known to be maintained over a wide range of environmental conditions and temperatures in *Arabidopsis* [84, 85]. RER values during the light period were comparable for plants grown in ML and plants grown in HL-MT and HL-LT. In contrast, HL-HT plants showed an increased RER in the light compared to the dark period. As HL-HT plants also had the highest biomass accumulation (Fig. 4D), results obtained with PS-Plant suggest that HL-HT plants were limited more by carbon turnover than CO_2_ assimilation. All plants grown in LL had a significantly decreased RER in the light compared to the dark period. Notably, temperature had no impact on RER in the light for LL plants, indicating that photosynthetic growth was primarily limited by the low irradiance. Further studies on carbon allocation and starch turnover should be carried out to complement these observations and hypotheses generated using PS-Plant data.

**Figure 5:**
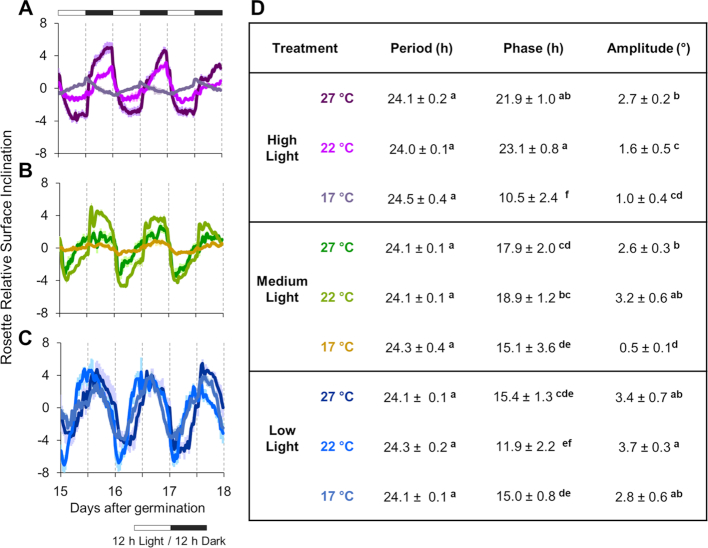
*Arabidopsis* plants grown under different conditions show differences in circadian movement. (**A-C**) The relative rosette surface inclination (i.e., rosette surface inclination following baseline detrending and alignment to the mean) for plants grown in high, medium, and low light from 15 to 18 DAG (see Supplementary Figure S3 for full data sets). (**D**) Period, phase, and amplitude calculated by the MFourFit method [87] using data from 11–24 DAG. Values are the mean ± SD of measurements made on ≥3 biological replicates. Values within each column followed by different letters are significantly different from each other and values followed by the same letter are not (*P* < 0.05) as determined by ANOVA followed by Tukey's HSD tests.

The internal circadian clock in plants has a periodicity close to 24 h that can be entrained by environmental cues [86]. Thus, we next used PS-Plant to examine the rhythmicity of total leaf movement (i.e., rosette surface inclination; see Fig. 3D) to compare the capacity of entrainment of the clock to different growth conditions (Fig. 5A–C; Supplementary Fig. S3) [68]. We compared 3 standard parameters: period, phase, and amplitude [86, 87].

As expected, all conditions showed a similar period for leaf movement of ∼24 h (*P* < 0.05) because all plants were grown in a 12:12 h light:dark cycle (Fig. 5D). However, phase and amplitude differed between growth conditions. Through all conditions peak phase occurred during the night, with the general observation that incremental increases in light intensity led to a phase delay in the peak. A possible exception is that in 17°C HL rhythms peaked at the end of the day. It is noteworthy that the 17°C ML and HL leaf rhythm traces are very low amplitude, most likely because these plants had very limited petiole growth. We also found that temperature affected the phase of the rhythm across all light conditions. For example, in both ML and HL growth at 27°C advanced the peak phase compared to 22°C.

Monitoring plant behaviour through time revealed the impact of light and temperature through development (Supplementary Fig. S3). A common trend is that warm temperatures increase mean rosette leaf inclination angle, or hyponasty, although the threshold for this response varied in the different light treatments. Another notable feature is that hyponasty and rhythm amplitude dampen over time. Our data show that under LL the leaf movement rhythms were more sinusoidal and higher amplitude rhythms than in ML and HL. Leaf movement rhythm waveforms of ML and HL were also quite different from LL, with some evidence of tracking dawn and dusk. Interestingly in HL the rhythm at 17°C was clearly in antiphase with 22°C and 27°C. Through time the 17°C rhythm dampened to high leaf hyponasty, while 22°C/27°C leaf rhythms dampened to a low leaf angle. In both cases this effect seems to arise from a gradual reduction in rhythmic regulation during the night period. Overall, these data illustrate that PS-Plant was able to extract quantitative data on a large range of traits associated with rhythmic leaf growth that are typically challenging to capture.

### Use of PS-Plant data and machine learning for accurate leaf segmentations

Our next goal was to examine the capacity of PS-Plant to track the phenotypic behaviour of individual leaves on a growing *Arabidopsis* rosette. To achieve this, we labelled individual leaves in 221 images of ML-MT rosettes (Supplementary Information S5) and used machine learning approaches to segment leaves [88]. We compared 2 available NN architectures, the end-to-end recurrent neural network with recurrent attention (RNN) [58] and the Mask R-convolutional neural network (R-CNN) [89], to examine the suitability of PS-Plant data for NNs designed for instance segmentation using RGB (colour) images. We focused on ML-MT plants because their growth was more uniform across different individuals compared to other growth conditions, which allowed the models to converge faster and achieve better results during the training process. The data set was split into 179 and 42 images (∼80:20 ratio) for training and validating the models, respectively. To avoid overfitting the model, we manually partitioned plant images for training and validation data sets to ensure that all time-series images of a single specimen appear in either training or validation data sets but not both.

PS-Plant produces a range of different data: from greyscale images to SN maps (e.g., Fig. 3). We trained the RNN and R-CNN architectures from initial random weights, while R-CNN was also pre-trained using transfer learning weights generated using the Common Objects in Context (COCO) data set [90]. The RNN and R-CNN architectures were trained using 3 different types of PS data to compare for segmentation accuracy: (i) composite (SN in *x* and *y* direction, and albedo for RGB layers), (ii) greyscale, and (iii) albedo images. All data used for training, including the raw PS-Plant data and rosette masks, are available as outlined in Supplementary Information S5. The obtained leaf segmentations were compared to the ground truth images using symmetric best dice (SBD; score of the accuracy of leaf instance segmentation) and foreground-background dice (FBD; score of the accuracy of rosette segmentation) evaluation formulas (Supplementary Information S3) [91].

**Figure 6:**
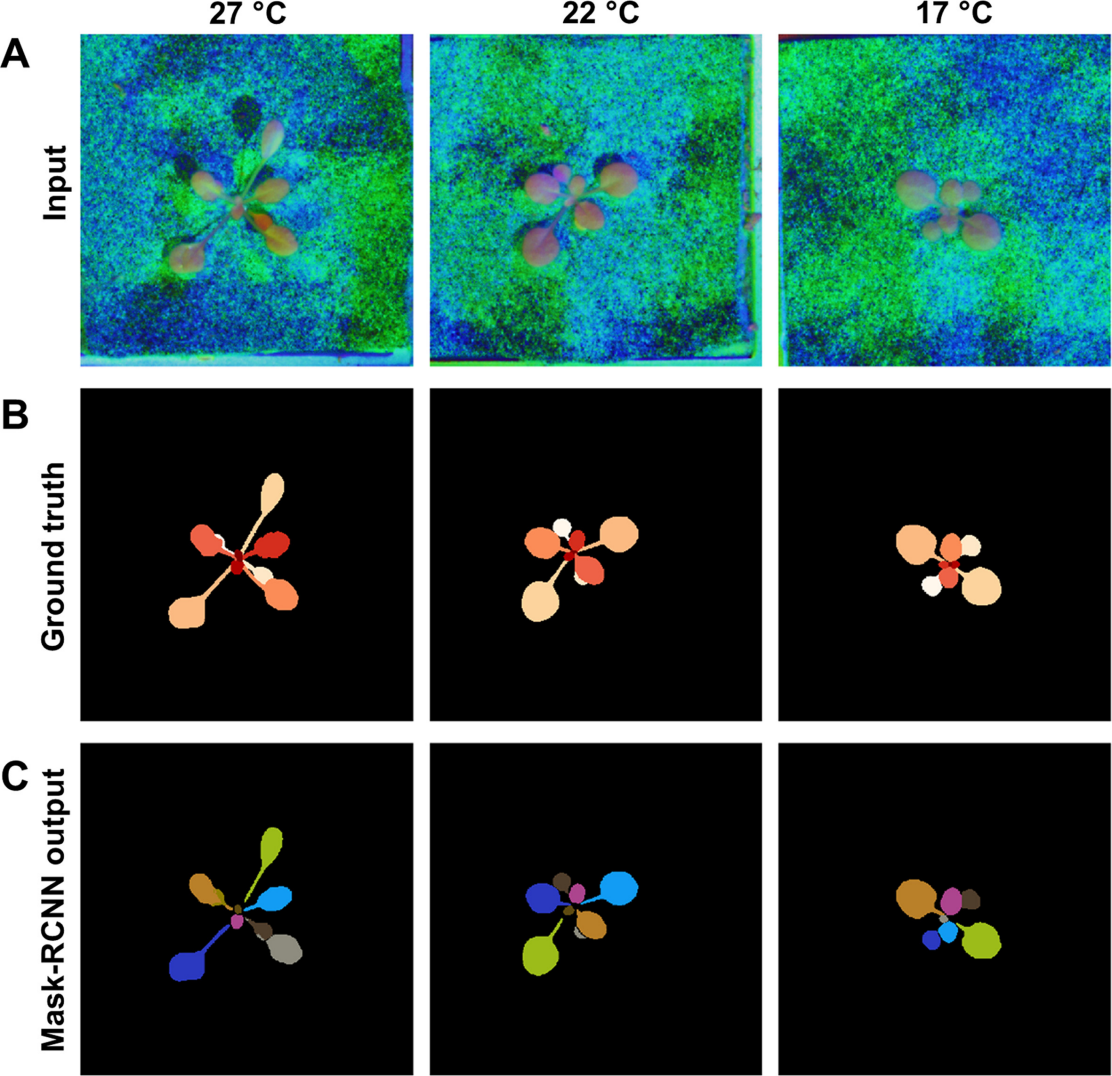
Automated segmentation of individual *Arabidopsis* leaves using PS-Plant data. Examples are shown based on the Mask R-CNN architecture for plants grown in ML at 3 different temperatures. (**A**) Composite input images are composed of surface normals in *x, y* directions and albedo data. (**B**) Manually labelled images (ground truth) used for training. (**C**) Mask R-CNN output images showing automated leaf segmentation. For ground truth images and Mask R-CNN outputs each leaf was assigned a unique arbitrary colour.

The type of PS data used did not significantly influence SBD or FBD scores, suggesting that the accuracy of RGB-based models was not affected by the different types of PS-based data. The most accurate leaf segmentation results were achieved with models based on the R-CNN architecture using pre-trained weights (Fig. 6; Table 1), resulting in SBD scores that ranged from 0.806 (composite image) to 0.814 (albedo). In comparison, the RNN architecture resulted in lower SBD scores ranging from 0.440 (composite image) to 0.560 (albedo). The pre-trained R-CNN model also achieved the most accurate rosette segmentation results, with FBD scores that ranged from 0.940 (albedo) to 0.946 (greyscale). In contrast, FBD scores for the RNN model varied from 0.798 (composite image) to 0.891 (albedo), indicating that the relative performance of the RNN architecture was worse for both leaf and rosette segmentation with our data sets when compared to the R-CNN approach.

**Table 1:** Performance comparison of leaf instance segmentation for 2 different machine learning architectures

Image type	Mask R-CNN				RNN	
	Random weights		Pre-trained weights		Random weights	
	SBD	FBD	SBD	FBD	SBD	FBD
Greyscale	0.813	0.942	0.812	0.946	0.556	0.866
Albedo	0.758	0.913	0.814	0.940	0.560	0.891
Surface normal map	0.789	0.922	0.806	0.941	0.440	0.798

The Mask R-CNN [89] and RNN [58] architectures were trained with composite (SN in *x* and *y* direction, and albedo for RGB layers), greyscale, or albedo images. The training procedure for the RNN architecture was the same as proposed by the authors [58], while the Mask R-CNN was as follows: head layers for 10 epochs at 10^−2^ learning rate (LR); all layers for 30 epochs at 10^−2^ LR, 30 epochs at 10^−3^ LR, 30 epochs at 10^−4^ LR, and head layers for 10 epochs at 10^−4^ LR. The Mask R-CNN was trained both from initial random weights and from pre-trained model weights, while RNN was only trained from initial random weights. SBD: symmetric best dice; FBD: foreground-background dice.

### Using PS-Plant data for dynamic tracking of individual leaf growth and movement

**Figure 7:**
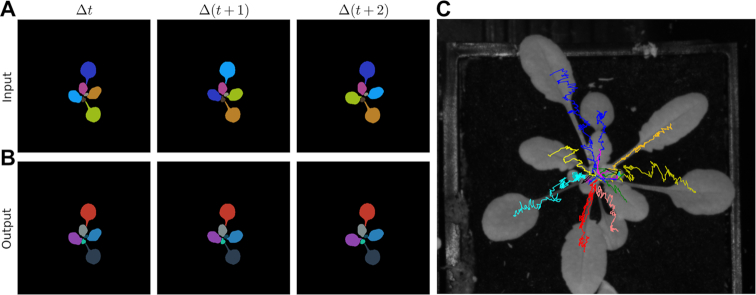
Automated tracking of leaf labels from segmented *Arabidopsis* rosettes. (**A**) Three consecutive frames for labelled leaves produced using the trained Mask R-CNN architecture (as in Fig. 6). (**B**) Tracked leaves retained the same colour after application of label tracking (see Rich Media 4). The particle filter allowed calibration of a variety of parameters, including span (the velocity of "span + 1" recent frames), search radius (the farthest distance [in pixels] an object may travel between frames), frame memory (the maximum number of frames a seen/tracked object that is absent will be remembered), and filter (the minimum number of frames in which an object must be seen/tracked to be included). The following particle filter settings produced the best results: span (10), search radius (30), frame memory (3), and filter (100). (**C**) Example of leaf tracking using leaf centroid locations. Each coloured line represents the movement of the centroid location of an individual leaf from 11 to 24 DAG.

We next investigated the performances of 4 different approaches for tracking leaves using the segmented image data sets (e.g., Fig. 6): (i) kernelized correlation filters [92], (ii) optical flow [93], (iii) multiple-instance learning tracker [94], and (iv) a particle filter [95]. Object tracking, especially with partially or even completely occluded objects, is one of the most challenging areas in computer vision [60, 61]. Tracking *Arabidopsis* leaves over time is particularly challenging owing to changes in both shape and movement during growth together with associated occlusions (Supplementary Information S4). The best results were achieved with a particle filter based on leaf instance centroid location and velocity across the time-series images (Fig. 7). Leaf overlap remained a limitation, as an occluding leaf was sometimes assigned the label of an occluded leaf. However, erroneous labelling was found to be infrequent and straightforward to manually correct post hoc, resulting in a robust semi-automated leaf tracker (Supplementary Data S4).

**Figure 8:**
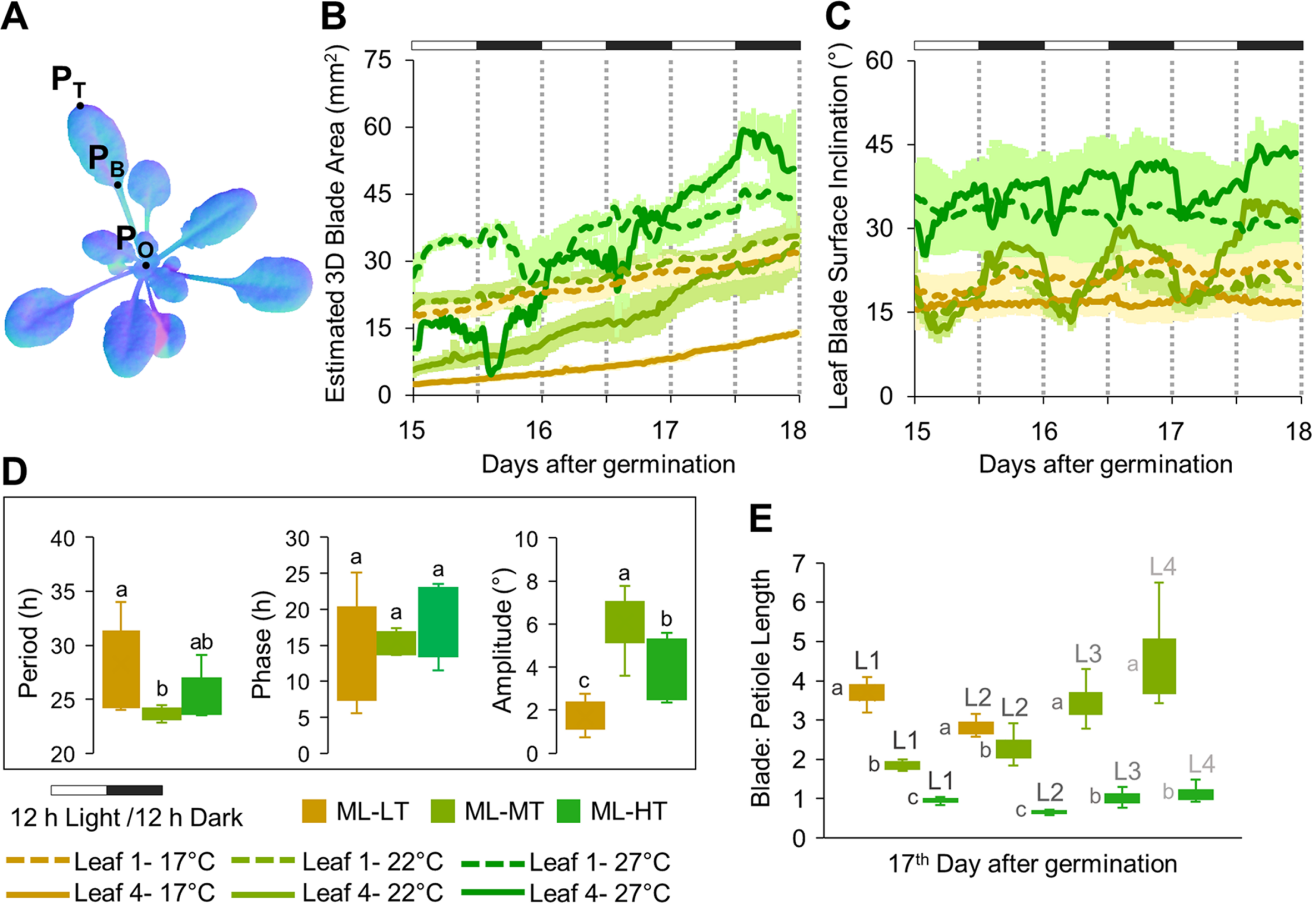
Analyses of growth and movement for individual leaves. (**A**) Key landmarks for leaf analysis: rosette origin (P_O_), leaf base/leaf blade and petiole intersection point (P_B_), and leaf tip (P_T_). Data are shown from plants grown in ML at 3 different temperatures (17°C [LT], 22°C [MT], or 27°C [HT]). (**B, C**) Leaf blade area and mean surface inclination of a maturing leaf (leaf 1) and an immature leaf (leaf 4) from 15 to 18 DAG. Error bars represent the mean ± SE of 3 separate leaves. (**D**) Period, phase, and amplitude values of the leaf blade from immature leaves (leaves 3 and 4; n = 6 leaves). Letters above the error bars indicate significant differences within each data type (*P* < 0.05) as determined by ANOVA followed by Tukey's HSD tests. Data sets with the same letter are not significantly different. (**E**) The ratio of leaf blade to petiole length for leaves 1–4 (L1–L4). Values represent the mean ratio over 24 h (17–18 DAG) for 3 separate leaves. Letters indicate significant differences (*P* < 0.05) within each leaf data set for different temperatures (i.e., L1, L2, L3, and L4).

Once we were confident that we could reliably track individual leaves using PS-Plant, we separated leaf blades and petioles by applying a morphological opening function with a predefined radius (3–11 pixels based on the leaf size) to the leaf binary mask. The point of differentiation (P_B_) is the mean *x* and *y* coordinates of the leaf blade and petiole (Fig. 8A). This enabled separate examinations of leaf blade and petiole traits. We then derived separated tissue-specific data including leaf blade area and inclination angle, and leaf blade and petiole length. The angle of leaf blade inclination was estimated using 2 different methods: (i) a point-based approach where leaf blade angle was determined using SN data across the line from P_B_ to the leaf tip (P_T_), and (ii) the mean surface inclination of the whole leaf blade. Both methods produced similar results (Supplementary Fig. S4). However, we chose to use the latter (ii) because the P_B_ was not always visible owing to leaf occlusions or the petiole being too small to be distinguished (e.g., maturing leaves or leaves grown in low temperature).

To demonstrate our approach, we tracked leaves 1–4 of plants grown in ML at 3 different temperatures from 15 to 18 DAG. Leaves 1–4 were chosen as representative examples of maturing (1 and 2) and immature (3 and 4) leaves (Fig. 8; Supplementary Data S5A–D). Consistent with our findings for PRA under different growth conditions (Fig. 4; Supplementary Fig. S1), the leaf blade areas of maturing and immature leaves from HT plants were significantly larger than leaf blades from MT and LT plants (as determined by 1-way ANOVA [*P* < 0.05], followed by Tukey's HSD tests; Fig. 8B). The latter results confirmed that the increased PRA observed using PS-Plant for plants grown in HT was specifically associated with an increase in leaf blade area. Leaves that emerged prior to the start of the experiment at 11 DAG (i.e., leaf 1) showed an increase in leaf blade area in HT plants compared to MT and LT plants (Fig. 8B). However, leaves that emerged after 11 DAG (i.e., leaf 4) had an even more dramatic growth response to increased temperatures. For example, the blade area for leaf 1 and 4 at 17 DAG was 40% and 130% higher in HT compared to LT, respectively. Similarly, the mean surface inclination of leaf blades was higher in HT (Fig. 8C). The latter result was also consistent with our findings for whole rosette surface inclination at higher temperatures (Figs 3 and 5; Supplementary Fig. S3).

We then calculated parameters associated with diurnal movement for individual leaf blades (Fig. 8D). We targeted immature leaf blades because their movement patterns were clearer and more consistent than those of maturing leaf blades. Period or phase measurements from immature leaf blades were generally similar between growth conditions and comparable to values for whole rosettes (Fig. 5). In contrast, measurements of immature leaf blade amplitude were significantly enhanced at MT and HT and generally higher than values for whole rosettes. This was not unexpected because immature leaves are more active than older leaves and contribute more to overall whole rosette amplitude (see Supplementary Data S3 and S5). Furthermore, the observed temperature-associated increases in amplitude and leaf hyponasty were consistent with whole-rosette data (Fig. 5D; Supplementary Fig. S3B). Thus, we concluded that measurements of periodic rhythms can be performed reliably with PS-Plant data using whole rosettes or individual leaf blades. The values obtained in the present study for period and phase are comparable to those reported for wild-type plants under standard growth conditions by other automated top-down systems for monitoring leaf movement, such as OSCILLATOR [96].

Finally, we used PS-Plant to reveal whether petiole elongation showed a response to temperature similar to that of the leaf blade by comparing the ratio of leaf blade and petiole length from maturing and immature leaves (Fig. 8E). Petioles have been shown to elongate faster at higher temperatures [76, 79, 97]. In the present study we observed that leaves from MT and LT plants had a blade-to-petiole length ratio that ranged from 2:1 to 4:1. Immature leaves did not have a detectable petiole under LL; thus, only maturing leaves were included at LT. In contrast, HT plants had ratios of approximately 1:1 for both maturing and immature leaves, indicating that HT resulted in an increased petiole elongation relative to leaf blade growth under ML. Future work should examine this ratio at different light intensities because petioles and leaf blades are known to have different responses to light. For example, petioles are known to elongate faster under LL while leaf blades grow more slowly [77, 98].

## Conclusion

In this article, we have introduced an adaptable and low-maintenance platform for affordable, advanced image-based phenotyping. A key goal was to ensure accessibility to the research community. In this regard, PS-Plant can be considered a powerful, alternative solution to 3D systems based on laser scanning and light-field camera technologies [36, 43], which is particularly well-suited for setup in low-income or developing countries. Our system exploits the richer data provided by PS-Plant with a combination of traditional image processing and machine learning techniques to extract rosette and leaf-level measurements in an automated manner. Here, we have demonstrated that PS-Plant is able to accurately monitor several growth traits and diurnal rhythms of different phenotypes of *Arabidopsis* plants produced in response to varied environments. This provides credibility that future work with PS-Plant will produce robust data for a wide variety of mutant phenotypes. Additionally, the concomitant quantification of overall growth, leaf traits, and circadian rhythms can facilitate a better understanding of the relationships among environment, plant yield, and internal molecular networks. Previous work has also highlighted that PS can capture high-resolution 3D surface details of leaf surface structures, such as leaf curvature and trichomes, which could be used to investigate dynamic changes in leaf development [50]. Research in plant phenotyping needs to focus on increasing accessibility and instituting effective data standards and management practices to assist with improving plant productivity and genetic gain [99, 100]. To help accelerate the latter, we have provided the PS training imaging data set from this study for community access (Supplementary Information S5) and a detailed protocol for software usage and data analysis with a test experimental data set (Supplementary Information S6). In its current design, PS-Plant is optimal for measuring growth traits in rosette-shaped plants such as *Arabidopsis*. However, we believe it can also be used during the seedling stage of other eudicot species (e.g., tomato, cabbage, oilseed rape) to analyse circadian rhythms by observing the rhythmic movements of cotyledons. Future work with PS-Plant will focus on improvements in leaf tracking [101], integration with spectral information [102], and incorporation of a low-cost depth camera to combine the high resolution of PS with a lower resolution depth map to characterize whole plants with more complex architectures.

## Materials and Methods

### Plant materials

*Arabidopsis* (*Arabidopsis thaliana* [L.] Heynh. Col-0) wild-type seeds were stratified for 2–3 days at 4°C. Each seed was placed in a square pot (50 mm) containing F2+S compost (Levington, Frimley, UK) covered in acrylic black felt fabric with a central hole (5 mm) and germinated at 22°C under white light (150 µmol photons m^−2^ s^−1^ at the plant level) in 12:12 h light:dark for 10 days in a Percival growth cabinet (SE‐41AR2, CLF Plant Climatics, Wertingen, Germany). For the plant area validation experiment, the plants were kept in this cabinet for 22 DAG. For imaging with PS-Plant, the seedlings were transferred to a Snijders growth cabinet (Microclima MC1000, CEC Technology, Helensburgh, UK).

### PS-Plant hardware

PS-Plant consists of a machine vision NIR monochrome camera (Grasshopper3 GS3-U3–41C6NIR-C, FLIR Systems, Wilsonville, USA) with a 16-mm fixed focal length lens (Kowa 1”SC LM25SC, Kowa Company Limited, Yokohama, Japan) with an NIR filter attached (LP920, MidOpt, Illinois, USA), 4 or 8 NIR LEDs (PowerStar IR 940 nm, Intelligent LED Solutions, Thatcham, UK), and an in-house–designed LED controller that allows rapid switching of LEDs using an Arduino platform (MKRZero, Arduino, Ivrea, Italy). The camera and LEDs were fixed on a square acrylic sheet (44 × 44 cm) and positioned at a height of 40 cm above the imaging plants (Fig.   1B and C). The camera was positioned centrally in the sheet and the LEDs were positioned around the camera at 45° angle increments. The LEDs were tilted at a 30° angle to illuminate the area under the camera field of view (Fig. 1B). The base of the rig was painted matt black to limit the introduction of specularities from the background. A laptop computer (K501UQ-DM050T, AsusTek Computer Inc., Taipei, Taiwan) was used to control LED illuminations and acquire, store, and process images using GUI software written in Python. Details on rig assembly and the LED controller design are outlined in Supplementary Information S2.

### Leaf movement rhythm analysis

The leaf movement rhythm analysis was performed using the mean inclination angles (whole rosette or individual leaf blade) as an input for BioDare2 beta [103]. The data were treated with baseline detrending prior to period, phase, and amplitude estimations, which was done using the MFourFit algorithm [87].

## Availability of source code and requirements

Project name: PS-Plant-Framework

Project home page: https://github.com/g2-bernotas/PS-Plant-Framework

Operating system(s): Platform independent (tested on Windows 7/10, Ubuntu 16.04)

Programming language: Python

Other requirements: Python v3.5, Python interpreter (Miniconda and Pyzo are suggested) and FlyCapture camera software packages (see Supplementary Information 6 for details)

License: GNU GPLv3 License

SciCrunch RRID:SCR_017032

## Availability of supporting data and materials

The training data set supporting the results of this article is available in an Edinburgh DataShare repository (https://datashare.is.ed.ac.uk/handle/10283/3280) and outlined in Supplementary Information S5. This data set represents approximately 0.4% of the 50,625 images captured during the “matrix” growth experiment (see Fig. 4A). A user protocol is available in Supplementary Information S6 to assist with software installation, and a test data set is available in the Edinburgh DataShare repository (https://datashare.is.ed.ac.uk/handle/10283/3279). Images of our training set and code, including other supporting data, are available in the *GigaScience* repository, GigaDB [88].

## Additional files

**Supplementary Information S1**. Overview of 2D image data processing captured using PS-Plant.

**Supplementary Information S2**. Overview of the PS-Plant hardware.

**Supplementary Information S3**. Formulas.

**Supplementary Information S4**. Area estimation errors.

**Supplementary Information S5**. PS-Plant training data set description.

**Supplementary Information S6**. PS-Plant protocol.

**Supplementary Figure S1**. Rosette and individual leaf growth analysis.

**Supplementary Figure S2**. Rosette compactness for plants grown in different conditions.

**Supplementary Figure S3**. Mean rosette surface inclinations for all growth conditions separated by light treatment.

**Supplementary Figure S4**. Estimated leaf inclination of leaf 1 in medium light and 27°C.

**Supplementary Data S1**. Interactive 3D model of the PS-Plant system. The model is provided as an .stl file (Rich Media 1.stl), and a zoomable, colour version can be found at https://bit.ly/2GXNhLy.

**Supplementary Data S2**. Comparison of *Arabidopsis* growth from 2D and 3D data. The graph (top) includes standard deviation of PRA data for 3 plants growing under conditions outlined in Fig. 3. Examples of plant growth are shown below for 2D (albedo; bottom left [see Supp. Info. 1 for details]) and surface normal map data (bottom right).

**Supplementary Data S3**. *Arabidopsis* plants grow and move differently under different light and temperature conditions. Examples of (**A**) surface normal models or (**B**) greyscale images for plants of the same age under each of the growth conditions tested (see Fig. 4) are shown from 11 to 24 DAG.

**Supplementary Data S4**. Automated tracking of individual *Arabidopsis* leaves. Example of leaf label tracking following rosette segmentation of an ML-MT plant shown from 15 to 18 DAG. Note that leaves retained the same colour after tracking (right).

**Supplementary Data S5**. Using PS-Plant for automated tracking of individual *Arabidopsis* leaf movement in 3 dimensions. Four videos illustrate leaf blade tracking of leaves 1–4, respectively, for a plant grown in ML-MT from 15 to 18 DAG. Each video shows a trail of leaf blade centroid movement (red dots) on an albedo 2D video (top left). Blue dots illustrate leaf blade movement on 2D *x-y* (bottom left) and *y-z* projections (bottom right), and a 3D *x-y-z* graph (top right).

giz056_GIGA-D-18-00459_Original_Submission

giz056_GIGA-D-18-00459_Revision_1

giz056_GIGA-D-18-00459_Revision_2

giz056_Response_to_Reviewer_Comments_Original_Submission

giz056_Response_to_Reviewer_Comments_Revision_1

giz056_Reviewer_1_Report_Original_Submission -- Chris Armit 12/4/2018 Reviewed

giz056_Reviewer_1_Report_Revision_1 -- Chris Armit 4/2/2019 Reviewed

giz056_Reviewer_2_Report_Original_Submission -- Nathan Miller 1/18/2019 Reviewed

giz056_Reviewer_2_Report_Revision_1 -- Nathan Miller 4/10/2019 Reviewed

giz056_Reviewer_3_Report_Original_Submission -- Ji Zhou 2/6/2019 Reviewed

giz056_Reviewer_3_Report_Revision_1 -- Ji Zhou 4/4/2019 Reviewed

giz056_Reviewer_4_Report_Original_Submission -- Dijun Chen 2/7/2019 Reviewed

giz056_Reviewer_4_Report_Revision_1 -- Dijun Chen 4/1/2019 Reviewed

giz056_Supplemental_Files

## Abbreviations

2D: 2-dimensional; 3D: 3-dimensional; ANOVA: analysis of variance; COCO: Common Objects in Context; DAG: days after germination; FBD: foreground-background dice score; GUI: graphical user interface; HL: high light; HSD: honest significant difference; HT: high temperature; LED: light-emitting diode; LiDAR: light detection and ranging (distance measurement method using pulsed laser light); LL: low light; LR: learning rate; LT: low temperature; ML: medium light; MRE: mean relative error; MT: medium temperature; NIR: near-infrared; NN: neural network; P_B_: leaf base point, or intersection point between leaf blade and petiole; P_O_: rosette origin point; PRA: projected rosette area; PS: photometric stereo; P_T_: leaf tip point; R-CNN: Mask R-CNN NN architecture; RER: relative expansion rate; RGB: red, green, and blue channels, or a colour image; RNN: end-to-end instance segmentation with recurrent attention NN architecture; rubisco: ribulose bisphosphate carboxylase/oxygenase; SBD: symmetric best dice score; SD: standard deviation; SN: surface normal.

## Consent for publication

This study abides by UK guidelines and legislation for plant science research.

## Competing interests

The authors declare that they have no competing interests.

## Funding

This work was supported by the UK Biotechnology and Biological Sciences Research Council grants BB/N02334X/1, BB/M025551/1, and BB/N005147/1. G.B. was funded by the University of the West of England (UWE) Partnership Fund.

## Authors’ contributions

G.B., M.F.H., and I.J.H. designed the hardware and software of the PS-Plant system including the image-processing pipeline. A.J.M., K.J.H., and L.C.T.S. designed the plant experimental setup. G.B. and L.C.T.S. performed and analysed the validation experiments. L.C.T.S. performed and analysed plant growth experiments. G.B. designed the study for NN model generation for leaf segmentation. A.J.M., L.C.T.S., and G.B. wrote the manuscript, with assistance from all authors. A.J.M., L.N.S., and M.L.S. supervised the project.

## References

[1] Meinke H Agricultural impacts: Europe's diminishing bread basket. Nat Clim Chang. 2014;4:541–2.

[2] Long SP, Marshall-Colon A, Zhu XG Meeting the global food demand of the future by engineering crop photosynthesis and yield potential. Cell. 2015;161:56–66. 25815985 10.1016/j.cell.2015.03.019

[3] Chew YH, Wenden B, Flis A, et al. Multiscale digital *Arabidopsis* predicts individual organ and whole-organism growth. Proc Natl Acad Sci U S A. 2015;112:E2556. 25197087 10.1073/pnas.1410238111 PMC4191812

[4] Elliott J, Deryng D, Müller C, et al. Constraints and potentials of future irrigation water availability on agricultural production under climate change. Proc Natl Acad Sci U S A. 2014;111:3239–44. 24344283 10.1073/pnas.1222474110 PMC3948288

[5] Cang FA, Wilson AA, Wiens JJ Climate change is projected to outpace rates of niche change in grasses. Biol Lett. 2016;12:20160368. 27677813 10.1098/rsbl.2016.0368 PMC5046922

[6] Liang X-Z, Wu Y, Chambers RG, et al. Determining climate effects on US total agricultural productivity. Proc Natl Acad Sci U S A. 2017;114:2285–92. 10.1073/pnas.1615922114 PMC5373336 28265075

[7] Ahmad J, Sun J, Smith L, et al. Improving photometric stereo through per-pixel light vector calculation. In: Martinez-Carranza J, ed. Electronic Proceedings of the 5th UK Computer Vision Student Workshop (BMVW), Bristol, UK 2013.

[8] Furbank RT, Tester M Phenomics - technologies to relieve the phenotyping bottleneck. Trends Plant Sci. 2011;16:635–44. 22074787 10.1016/j.tplants.2011.09.005

[9] Dobrescu A, Scorza LCT, Tsaftaris SA, et al. A “do-it-yourself” phenotyping system: Measuring growth and morphology throughout the diel cycle in rosette shaped plants. Plant Methods. 2017;13:1–12. 29151842 10.1186/s13007-017-0247-6 PMC5678596

[10] Shakoor N, Lee S, Mockler TC High throughput phenotyping to accelerate crop breeding and monitoring of diseases in the field. Curr Opin Plant Biol. 2017;38:184–92. 28738313 10.1016/j.pbi.2017.05.006

[11] Underwood J, Wendel A, Schofield B, et al. Efficient in-field plant phenomics for row-crops with an autonomous ground vehicle. J Field Robot. 2017;34:1061–83.

[12] Ruckelshausen A, Biber P, Dorna M, et al. BoniRob: an autonomous field robot platform for individual plant phenotyping. Precis Agric. 2009;9:841–7.

[13] Tattaris M, Reynolds MP, Chapman SC A direct comparison of remote sensing approaches for high-throughput phenotyping in plant breeding. Front Plant Sci. 2016;7:1–9. 27536304 10.3389/fpls.2016.01131 PMC4971441

[14] Sankaran S, Khot LR, Espinoza CZ, et al. Low-altitude, high-resolution aerial imaging systems for row and field crop phenotyping: A review. Eur J Agron. 2015;70:112–23.

[15] Virlet N, Sabermanesh K, Sadeghi-Tehran P, et al. Field Scanalyzer: An automated robotic field phenotyping platform for detailed crop monitoring. Funct Plant Biol. 2017;44:143–53. 10.1071/FP16163 32480553

[16] da Costa RMF, Simister R, Roberts LA, et al. Nutrient and drought stress: implications for phenology and biomass quality in *Miscanthus*. Ann Bot. 2018,doi:10.1093/aob/mcy155. PMC6821376 30137291

[17] Tester M, Awlia M, Brown T, et al. Using phenomic analysis of photosynthetic function for abiotic stress response gene discovery. Arabidopsis Book. 2016;14:e0185. 27695390 10.1199/tab.0185 PMC5042155

[18] Tardieu F, Cabrera-Bosquet L, Pridmore T, et al. Plant phenomics, from sensors to knowledge. Curr Biol. 2017;27:770–83. 10.1016/j.cub.2017.05.055 28787611

[19] Araus JL, Kefauver SC Breeding to adapt agriculture to climate change: affordable phenotyping solutions. Curr Opin Plant Biol. 2018;45:1–11. 29853283 10.1016/j.pbi.2018.05.003

[20] Sharma RC Selection for biomass yield in wheat. Euphytica. 1993;70:35–42.

[21] Richards RA Selectable traits to increase crop photosynthesis and yield of grain crops. J Exp Bot. 2000;51:447–58. 10938853 10.1093/jexbot/51.suppl_1.447

[22] Arora VK, Singh CB, Sidhu AS, et al. Irrigation, tillage and mulching effects on soybean yield and water productivity in relation to soil texture. Agric Water Manag. 2011;98:563–8.

[23] Zhang H, Flottmann S Seed yield of canola (*Brassica napus* L.) is determined primarily by biomass in a high-yielding environment. Crop Pasture Sci. 2016;67:369–80.

[24] Zhang H, Flottmann S Genotypic variation in the accumulation of water-soluble carbohydrate in canola and its potential contribution to seed yield in different environments. Field Crop Res. 2016;196:124–33.

[25] Vázquez-Arellano M, Griepentrog HW, Reiser D, et al. 3-D imaging systems for agricultural applications—a review. Sensors (Basel). 2016;16:E618. 27136560 10.3390/s16050618 PMC4883309

[26] Green JM, Appel H, Rehrig EM, et al. PhenoPhyte: A flexible affordable method to quantify 2D phenotypes from imagery. Plant Methods. 2012;8:1–12. 23131141 10.1186/1746-4811-8-45 PMC3546069

[27] Dhondt S, Gonzalez N, Blomme J, et al. High-resolution time-resolved imaging of *in vitro Arabidopsis* rosette growth. Plant J. 2014;80:172–84. 25041085 10.1111/tpj.12610

[28] Minervini M, Giuffrida MV, Perata P, et al. Phenotiki: an open software and hardware platform for affordable and easy image-based phenotyping of rosette-shaped plants. Plant J. 2017;90:204–16. 28066963 10.1111/tpj.13472

[29] Chen JM, Black TA Defining leaf area index for non‐flat leaves. Plant Cell Environ. 1992;15:421–9.

[30] Bianco G, Gallo A, Bruno F, et al. A comparative analysis between active and passive techniques for underwater 3D reconstruction of close-range objects. Sensors (Basel). 2013;13:11007–31. 23966193 10.3390/s130811007 PMC3812639

[31] Pound MP, French AP, Fozard JA, et al. A patch-based approach to 3D plant shoot phenotyping. Mach Vis Appl. 2016;27:767–79.

[32] Pound MP, French AP, Murchie EH, et al. Automated recovery of three-dimensional models of plant shoots from multiple color images. Plant Physiol. 2014;166:1688–98. 25332504 10.1104/pp.114.248971 PMC4256878

[33] Biskup B, Scharr H, Schurr U, et al. A stereo imaging system for measuring structural parameters of plant canopies. Plant Cell Environ. 2007;30:1299–308. 17727419 10.1111/j.1365-3040.2007.01702.x

[34] Burgess AJ, Retkute R, Pound MP, et al. Image-based 3D canopy reconstruction to determine potential productivity in complex multi-species crop systems. Ann Bot. 2017;119:517–32. 28065926 10.1093/aob/mcw242 PMC5458713

[35] Jay S, Rabatel G, Hadoux X, et al. In-field crop row phenotyping from 3D modeling performed using Structure from Motion. Comput Electron Agric. 2015;110:70–7.

[36] Apelt F, Breuer D, Nikoloski Z, et al. Phytotyping^4D^: A light-field imaging system for non-invasive and accurate monitoring of spatio-temporal plant growth. Plant J. 2015;82:693–706. 25801304 10.1111/tpj.12833

[37] Gibbs J, Pound M, French A, et al. Plant phenotyping: an active vision cell for three-dimensional plant shoot reconstruction. Plant Physiol. 2018;178(2):524–34. 30097468 10.1104/pp.18.00664 PMC6181042

[38] Tippetts B, Lee DJ, Lillywhite K, et al. Review of stereo vision algorithms and their suitability for resource-limited systems. J Real-Time Image Process. 2016;11:5–25.

[39] Xiong X, Yu L, Yang W, et al. A high-throughput stereo-imaging system for quantifying rape leaf traits during the seedling stage. Plant Methods. 2017;13:1–17. 28163771 10.1186/s13007-017-0157-7 PMC5282657

[40] Apelt F, Breuer D, Olas JJ, et al. Circadian, carbon, and light control of expansion growth and leaf movement. Plant Physiol. 2017;174:1949–68. 28559360 10.1104/pp.17.00503 PMC5490918

[41] Nguyen TT, Slaughter DC, Max N, et al. Structured light-based 3D reconstruction system for plants. Sensors (Basel). 2015;15:18587–612. 26230701 10.3390/s150818587 PMC4570338

[42] Paulus S, Schumann H, Kuhlmann H, et al. High-precision laser scanning system for capturing 3D plant architecture and analysing growth of cereal plants. Biosyst Eng. 2014;121:1–11.

[43] Dornbusch T, Michaud O, Xenarios I, et al. Differentially phased leaf growth and movements in *Arabidopsis* depend on coordinated circadian and light regulation. Plant Cell. 2014;26:3911–21. 25281688 10.1105/tpc.114.129031 PMC4247567

[44] Dornbusch T, Lorrain Ś, Kuznetsov D, et al. Measuring the diurnal pattern of leaf hyponasty and growth in *Arabidopsis*—a novel phenotyping approach using laser scanning. Funct Plant Biol. 2012;39:860–9. 10.1071/FP12018 32480836

[45] Paulus S, Eichert T, Goldbach HE, et al. Limits of active laser triangulation as an instrument for high precision plant imaging. Sensors (Basel). 2014;14:2489–509. 24504106 10.3390/s140202489 PMC3958280

[46] Herrero-Huerta M, Lindenbergh R, Gard W Leaf movements of indoor plants monitored by terrestrial LiDAR. Front Plant Sci. 2018;9:189. 29527217 10.3389/fpls.2018.00189 PMC5829619

[47] Thapa S, Zhu F, Walia H, et al. A novel LiDAR-based instrument for high-throughput, 3D measurement of morphological traits in maize and sorghum. Sensors (Basel). 2018;18, doi:10.3390/s18041187. PMC5948551 29652788

[48] Woodham RJ Photometric method for determining surface orientation from multiple images. Opt Eng. 1980;19:139–44.

[49] Smith LN, Zhang W, Hansen MF, et al. Innovative 3D and 2D machine vision methods for analysis of plants and crops in the field. Comput Ind. 2018;97:122–31. 29997402 10.1016/j.compind.2018.02.002 PMC6034441

[50] Zhang W, Hansen MF, Smith M, et al. Photometric stereo for three-dimensional leaf venation extraction. Comput Ind. 2018;98:56–67. 29997404 10.1016/j.compind.2018.02.006 PMC6034445

[51] Singh A, Ganapathysubramanian B, Singh AK, et al. Machine learning for high-throughput stress phenotyping in plants. Trends Plant Sci. 2016;21:110–24. 26651918 10.1016/j.tplants.2015.10.015

[52] Pound MP, Atkinson JA, Townsend AJ, et al. Deep machine learning provides state-of-the-art performance in image-based plant phenotyping. Gigascience. 2017;6:1–10. 10.1093/gigascience/gix083 PMC5632296 29020747

[53] Gonzalez N, Beemster GT, Inzé D David and Goliath: what can the tiny weed *Arabidopsis* teach us to improve biomass production in crops?. Curr Opin Plant Biol. 2009;12:157–64. 19119056 10.1016/j.pbi.2008.11.003

[54] De Vylder J, Vandenbussche F, Hu Y, et al. Rosette tracker: an open source image analysis tool for automatic quantification of genotype effects. Plant Physiol. 2012;160:1149–59. 22942389 10.1104/pp.112.202762 PMC3490612

[55] Zhou J, Applegate C, Alonso AD, et al. Leaf-GP: An open and automated software application for measuring growth phenotypes for *Arabidopsis* and wheat. Plant Methods. 2017;13:1–17. 29299051 10.1186/s13007-017-0266-3 PMC5740932

[56] Tomé F, Jansseune K, Saey B, et al. rosettr: protocol and software for seedling area and growth analysis. Plant Methods. 2017;13:1–10. 28331535 10.1186/s13007-017-0163-9 PMC5353781

[57] Aich S, Stavness I Leaf counting with deep convolutional and deconvolutional networks. In: Proceedings of the IEEE International Conference on Computer Vision Workshops (ICCVW) 2017:2080–9.

[58] Ren M, Zemel RS End-to-End instance segmentation with recurrent attention. In: 2017 IEEE Conference on Computer Vision and Pattern Recognition (CVPR). 21–6.

[59] Ubbens J, Cieslak M, Prusinkiewicz P, et al. The use of plant models in deep learning: An application to leaf counting in rosette plants. Plant Methods. 2018;14:1–10. 29375647 10.1186/s13007-018-0273-z PMC5773030

[60] Yang H, Shao L, Zheng F, et al. Recent advances and trends in visual tracking: A review. Neurocomputing. 2011;74:3823–31.

[61] Smeulders AWM, Chu DM, Cucchiara R, et al. Visual tracking: An experimental survey. IEEE Trans Pattern Anal Mach Intell. 2014;36:1442–68. 26353314 10.1109/TPAMI.2013.230

[62] Azhar F, Emrith K, Pollard S, et al. Testing the validity of Lambert's law for micro-scale photometric stereo applied to paper substrates. In: Proceedings of the 10th International Conference on Computer Vision Theory and Applications, Berlin, Germany, 2015. 2015:246–53.

[63] Otsu N A threshold selection method from gray-level histograms. IEEE Trans Syst Man Cybern. 1979;9:62–6.

[64] Jacquemoud S, Baret F PROSPECT: A model of leaf optical properties spectra. Remote Sens Environ. 1990;34:75–91.

[65] Chelle M Could plant leaves be treated as Lambertian surfaces in dense crop canopies to estimate light absorption?. Ecol Modell. 2006;198:219–28.

[66] Karidas P, Challa KR, Nath U The tarani mutation alters surface curvature in *Arabidopsis* leaves by perturbing the patterns of surface expansion and cell division. J Exp Bot. 2015;66:2107–22. 25711708 10.1093/jxb/erv015 PMC4378639

[67] Sandalio LM, Rodríguez-Serrano M, Romero-Puertas MC Leaf epinasty and auxin: A biochemical and molecular overview. Plant Sci. 2016;253:187–93. 27968987 10.1016/j.plantsci.2016.10.002

[68] Edwards KD, Millar AJ Analysis of circadian leaf movement rhythms in *Arabidopsis thaliana*. Methods Mol Biol. 2007;362:103–13. 17417004 10.1007/978-1-59745-257-1_7

[69] Mizoguchi T, Wheatley K, Hanzawa Y, et al. LHY and CCA1 are partially redundant genes required to maintain circadian rhythms in *Arabidopsis*. Dev Cell. 2002;2:629–41. 12015970 10.1016/s1534-5807(02)00170-3

[70] Doyle MR, Davis SJ, Bastow RM, et al. The ELF4 gene controls circadian rhythms and flowering time in *Arabidopsis thaliana*. Nature. 2002;419:74–7. 12214234 10.1038/nature00954

[71] Yoo SK, Hong SM, Lee JS, et al. A genetic screen for leaf movement mutants identifies a potential role for AGAMOUS-LIKE 6 (AGL6) in circadian-clock control. Mol Cells. 2011;31:281–7. 21331777 10.1007/s10059-011-0035-5 PMC3932699

[72] Jansen M, Gilmer F, Biskup B, et al. Simultaneous phenotyping of leaf growth and chlorophyll fluorescence via GROWSCREEN FLUORO allows detection of stress tolerance in *Arabidopsis thaliana* and other rosette plants. Funct Plant Biol. 2009;36(11):902–14. 10.1071/FP09095 32688701

[73] Casal JJ Shade avoidance. Arabidopsis Book. 2012;10, doi:10.1199/tab.0157. PMC3350169 22582029

[74] Pierik R, De Wit M Shade avoidance: Phytochrome signalling and other aboveground neighbour detection cues. J Exp Bot. 2014;65:2815–24. 24323503 10.1093/jxb/ert389

[75] Gommers CMM, Keuskamp DH, Buti S, et al. Molecular profiles of contrasting shade response strategies in wild plants: differential control of immunity and shoot elongation. Plant Cell. 2017;29:331–44. 28138015 10.1105/tpc.16.00790 PMC5354195

[76] Quint M, Delker C, Franklin KA, et al. Molecular and genetic control of plant thermomorphogenesis. Nat Plants. 2016;2:1–9. 10.1038/nplants.2015.190 27250752

[77] de Wit M, Ljung K, Fankhauser C Contrasting growth responses in lamina and petiole during neighbor detection depend on differential auxin responsiveness rather than different auxin levels. New Phytol. 2015;208:198–209. 25963518 10.1111/nph.13449

[78] Vile D, Pervent M, Belluau M, et al. *Arabidopsis* growth under prolonged high temperature and water deficit: Independent or interactive effects?. Plant Cell Environ. 2012;35:702–18. 21988660 10.1111/j.1365-3040.2011.02445.x

[79] Crawford AJ, McLachlan DH, Hetherington AM, et al. High temperature exposure increases plant cooling capacity. Curr Biol. 2012;22:R396–7. 22625853 10.1016/j.cub.2012.03.044

[80] Berry J, Bjorkman O Photosynthetic response and adaptation to temperature in higher plants. Annu Rev Plant Physiol. 1980;31:491–543.

[81] Bunce JA Acclimation of photosynthesis to temperature in eight cool and warm climate herbaceous C(3) species: Temperature dependence of parameters of a biochemical photosynthesis model. Photosynth Res. 2000;63:59–67. 16252165 10.1023/A:1006325724086

[82] Yamori W, von Caemmerer S Effect of rubisco activase deficiency on the temperature response of CO_2_ assimilation rate and rubisco activation state: insights from transgenic tobacco with reduced amounts of rubisco activase. Plant Physiol. 2009;151:2073–82. 19837817 10.1104/pp.109.146514 PMC2786000

[83] Wiese A, Christ MM, Virnich O, et al. Spatio-temporal leaf growth patterns of *Arabidopsis thaliana* and evidence for sugar control of the diel leaf growth cycle. New Phytol. 2007;174:752–61. 17504459 10.1111/j.1469-8137.2007.02053.x

[84] Smith AM, Stitt M Coordination of carbon supply and plant growth. Plant Cell Environ. 2007;30:1126–49. 17661751 10.1111/j.1365-3040.2007.01708.x

[85] Pyl E-T, Piques M, Ivakov A, et al. Metabolism and growth in *Arabidopsis* depend on the daytime temperature but are temperature-compensated against cool nights. Plant Cell. 2012;24:2443–69. 22739829 10.1105/tpc.112.097188 PMC3406903

[86] McClung CR Plant circadian rhythms. Plant Cell. 2006;18:792–803. 16595397 10.1105/tpc.106.040980 PMC1425852

[87] Zielinski T, Moore AM, Troup E, et al. Strengths and limitations of period estimation methods for circadian data. PLoS One. 2014;9:1–26. 10.1371/journal.pone.0096462 PMC4014635 24809473

[88] Bernotas G, Scorza LCT, Hansen MF, et al. Supporting data for “A photometric stereo-based 3D imaging system using computer vision and deep learning for tracking plant growth.”. GigaScience Database. 2019 10.5524/100594. PMC6534809 31127811

[89] He K, Gkioxari G, Dollar P, et al. Mask R-CNN. 2017 IEEE International Conference on Computer Visions. 2017;2980–88., doi:10.1109/ICCV.2017.322.

[90] Lin TY, Maire M, Belongie S, et al. Microsoft COCO: Common Objects in Context. In: Fleet D, Pajdla T, Schiele B et al., et al., eds. Computer Vision – ECCV 2014. 2014;740–55.

[91] Scharr H, Minervini M, French AP, et al. Leaf segmentation in plant phenotyping: a collation study. Mach Vis Appl. 2016;27:585–606.

[92] Henriques JF, Caseiro R, Martins P, et al. High-speed tracking with kernelized correlation filters. IEEE Trans Pattern Anal Mach Intell. 2015;37:583–96. 26353263 10.1109/TPAMI.2014.2345390

[93] Lucas BD, Kanade T An iterative image registration technique with an application to stereo vision. Proc 7th Intl Joint Conf on Artificial Intelligence (IJCAI).1981:674–79.

[94] Babenko B, Yang M-H, Belongie S Visual tracking with online multiple instance learning. In: 2009 IEEE Conference on Computer Vision and Pattern Recognition, Miami, FL. 2009:983–90.

[95] Allan D, Caswell T, Keim N, et al. trackpy: Trackpy v0.3.2. Zenodo. 2016, doi:10.5281/zenodo.60550.

[96] Bours R, Muthuraman M, Bouwmeester H, et al. OSCILLATOR: A system for analysis of diurnal leaf growth using infrared photography combined with wavelet transformation. Plant Methods. 2012;8:1–12. 22867627 10.1186/1746-4811-8-29 PMC3489599

[97] van Zanten M, Voesenek LACJ, Peeters AJM, et al. Hormone- and light-mediated regulation of heat-induced differential petiole growth in *Arabidopsis*. Plant Physiol. 2009;151:1446–58. 19741046 10.1104/pp.109.144386 PMC2773053

[98] Kozuka T, Horiguchi G, Kim GT, et al. The different growth responses of the *Arabidopsis thaliana* leaf blade and the petiole during shade avoidance are regulated by photoreceptors and sugar. Plant Cell Physiol. 2005;46:213–23. 15659441 10.1093/pcp/pci016

[99] Coppens F, Wuyts N, Inzé D, et al. Unlocking the potential of plant phenotyping data through integration and data-driven approaches. Curr Opin Syst Biol. 2017;4:58–63. 10.1016/j.coisb.2017.07.002 PMC7477990 32923745

[100] Araus JL, Kefauver SC, Zaman-Allah M, et al. Translating high-throughput phenotyping into genetic gain. Trends Plant Sci. 2018;23:451–66. 29555431 10.1016/j.tplants.2018.02.001 PMC5931794

[101] Valmadre J, Bertinetto L, Henriques JF, et al. End-to-end representation learning for Correlation Filter based tracking. In: 2017 IEEE Conference on Computer Vision and Pattern Recognition. 2017:5000–8.

[102] Li L, Zhang Q, Huang D A review of imaging techniques for plant phenotyping. Sensors (Basel). 2014;14:20078–111. 25347588 10.3390/s141120078 PMC4279472

[103] BioDare2 beta. https://biodare2.ed.ac.uk/. Accessed 1st March 2019

