# Supplementary material for: A photometric stereo-based 3D imaging system using computer vision and deep learning for tracking plant growth
Source: Gigascience. 2019 May 25;8(5):giz056. doi: 10.1093/gigascience/giz056 (PMC6534809; doi:10.1093/gigascience/giz056)
Supplement: giz056_GIGA-D-18-00459_Original_Submission [file giz056_giga-d-18-00459_original_submission.pdf]

GigaScience

A photometric stereo-based 3D imaging system using computer vision and deep learning for tracking plant growth

--Manuscript Draft--

**Manuscript Number:**	GIGA-D-18-00459
**Full Title:**	A photometric stereo-based 3D imaging system using computer vision and deep learning for tracking plant growth
**Article Type:**	Research
**Funding Information:**	Biotechnology and Biological Sciences Research Council (BB/N02334X/1) — Dr Alistair McCormick<br>Biotechnology and Biological Sciences Research Council (BB/M025551/1) — Prof. Karen J Halliday<br>Biotechnology and Biological Sciences Research Council (BB/N005147/1) — Prof. Karen J Halliday
**Abstract:**	Background: Tracking and predicting the growth performance of plants in different environments is critical for future crop development, which is under dual pressure from population expansion and global climate change. Automated approaches for image capture and analysis have allowed for substantial increases in the throughput of quantitative growth trait measurements compared to manual assessments. Recent work has focused on adopting computer vision and machine learning approaches to improve the accuracy of automated plant phenotyping. Here we present PS-Plant, a low-cost and portable 3D plant phenotyping platform based on an imaging technique novel to plant phenotyping called photometric stereo (PS).<br><br>Results: We calibrated PS-Plant to track the model plant Arabidopsis thaliana throughout the day-night (diel) cycle and investigated growth architecture under a variety of conditions to illustrate the dramatic effect of the environment on plant phenotype. We developed bespoke computer vision algorithms and assessed available deep neural network architectures to automate the segmentation of rosettes and individual leaves, and extract basic and more advanced traits from PS-derived data, including the tracking of 3D plant growth and diel leaf hyponastic movement. Furthermore, we have produced the first PS data set, which includes 221 manually annotated Arabidopsis rosettes that were used for training and data analysis (1768 images in total).<br><br>Conclusions: PS-Plant is a powerful new phenotyping tool for plant research that provides robust data at high temporal and spatial resolutions. The system is well-suited for small and large-scale research and will help to accelerate bridging of the phenotype-to-genotype gap.
**Corresponding Author:**	Alistair McCormick, Ph.D<br>University of Edinburgh<br>Edinburgh, Edinburgh UNITED KINGDOM
**Corresponding Author Secondary Information:**	
**Corresponding Author's Institution:**	University of Edinburgh
**Corresponding Author's Secondary Institution:**	
**First Author:**	Gytis Bernotas
**First Author Secondary Information:**	
**Order of Authors:**	Gytis Bernotas<br>Livia C.T. Scorza, Ph.D

Powered by Editorial Manager® and ProduXion Manager® from Aries Systems Corporation

	Mark F. Hansen, Ph.D
	Ian J J Hales, Ph.D
	Karen J Halliday
	Lyndon N Smith, Ph.D
	Melvyn L Smith, Ph.D
	Alistair McCormick, Ph.D
**Order of Authors Secondary Information:**	
**Additional Information:**	
**Question**	**Response**
Are you submitting this manuscript to a special series or article collection?	No
**Experimental design and statistics**<br><br>Full details of the experimental design and statistical methods used should be given in the Methods section, as detailed in our Minimum Standards Reporting Checklist. Information essential to interpreting the data presented should be made available in the figure legends.<br><br>Have you included all the information requested in your manuscript?	Yes
**Resources**<br><br>A description of all resources used, including antibodies, cell lines, animals and software tools, with enough information to allow them to be uniquely identified, should be included in the Methods section. Authors are strongly encouraged to cite Research Resource Identifiers (RRIDs) for antibodies, model organisms and tools, where possible.<br><br>Have you included the information requested as detailed in our Minimum Standards Reporting Checklist?	Yes
**Availability of data and materials**<br><br>All datasets and code on which the	Yes

conclusions of the paper rely must be either included in your submission or deposited in publicly available repositories (where available and ethically appropriate), referencing such data using a unique identifier in the references and in the "Availability of Data and Materials" section of your manuscript.<br><br>Have you have met the above requirement as detailed in our Minimum Standards Reporting Checklist?	

Manuscript

Click here to download Manuscript Manuscript text - with figs.docx

A photometric stereo-based 3D imaging system using computer vision and deep learning for tracking plant growth

Gytis Bernotas[1*], Livia C T Scorza[2*], Mark F Hansen[1*], Ian J Hales[1], Karen J Halliday[2], Lyndon N Smith[1], Melvyn L Smith[1], Alistair J McCormick[2,†]

[1] Centre for Machine Vision, Bristol Robotics Laboratory, University of the West of England, T block, Frenchay Campus, Coldharbour Lane, Bristol, BS16 1QY, UK

[2] SynthSys & Institute of Molecular Plant Sciences, School of Biological Sciences, University of Edinburgh, Edinburgh, EH9 3BF, UK

[*]co-first authors ; [†] Corresponding author

Article type: Research

Email addresses

Authors:	Emails	ORCID
Gytis Bernotas:	gytis.bernotas@uwe.ac.uk	0000-0002-3418-5758
Livia C T Scorza:	livia.scorza@ed.ac.uk	0000-0002-0145-3592
Mark F Hansen:	mark.hansen@uwe.ac.uk	0000-0003-4681-6251
Ian J Hales:	contact@ian-hales.com	
Karen Halliday:	karen.halliday@ed.ac.uk	0000-0003-0467-104X
Lyndon N Smith:	lyndon.smith@uwe.ac.uk	
Melvyn L Smith:	melvyn.smith@uwe.ac.uk	
Alistair McCormick:	alistair.mccormick@ed.ac.uk	0000-0002-7255-872X

[†]corresponding author:

Dr Alistair J. McCormick

Daniel Rutherford Building, SynthSys & Institute of Molecular Plant Sciences

School of Biological Sciences, University of Edinburgh

The King's Buildings, EH9 3BF Phone: +44 (0)1316505316

Abstract

Background: Tracking and predicting the growth performance of plants in different environments is critical for future crop development, which is under dual pressure from population expansion and global climate change. Automated approaches for image capture and analysis have allowed for substantial increases in the throughput of quantitative growth trait measurements compared to manual assessments. Recent work has focused on adopting computer vision and machine learning approaches to improve the accuracy of automated plant phenotyping. Here we present PS-Plant, a low-cost and portable 3D plant phenotyping platform based on an imaging technique novel to plant phenotyping called photometric stereo (PS).

Results: We calibrated PS-Plant to track the model plant *Arabidopsis thaliana* throughout the day-night (diel) cycle and investigated growth architecture under a variety of conditions to illustrate the dramatic effect of the environment on plant phenotype. We developed bespoke computer vision algorithms and assessed available deep neural network architectures to automate the segmentation of rosettes and individual leaves, and extract basic and more advanced traits from PS-derived data, including the tracking of 3D plant growth and diel leaf hyponastic movement. Furthermore, we have produced the first PS data set, which includes 221 manually annotated *Arabidopsis* rosettes that were used for training and data analysis (1768 images in total).

Conclusions: PS-Plant is a powerful new phenotyping tool for plant research that provides robust data at high temporal and spatial resolutions. The system is well-suited for small and large-scale research and will help to accelerate bridging of the phenotype-to-genotype gap.

Keywords

Arabidopsis thaliana, leaf angle, segmentation, machine learning, near-infrared (NIR) LEDs, photomorphogenesis, thermomorphogenesis.

Introduction

To feed our growing global population, the productivity of staple crops will require an increase in yields of approximately 85% by 2050 [1]. Quantitative and accurate methods are required to aid strategies for improving plant performance and predict yields in our changeable natural environments. Such tools are critical for calibrating predictive models in the face of a changing global climate [2–6]. Computer vision is an evolving technology that is helping to drive advances in plant phenotyping both in fundamental research and agriculture [7–10]. Reflecting its considerable promise, effort has been directed toward automated ground vehicles (AGVs), satellite, drone and gantry-style platform imaging of field plants, and automated phenotyping of greenhouse and lab-grown plants [11,12]. While there have been significant advances, problems associated with high cost, automated data capture, large data sets and variable visual and temporal resolutions have created barriers to the uptake of these technologies. These challenges are currently being addressed in the next generation of plant phenotyping tools.

Above ground growth is a strong indicator of plant yield and therefore 3D imaging of vegetative growth is a very active area of phenotyping research [13–18]. A number of excellent 2D imaging systems have been developed [19–21], however, while they represent a qualitative improvement on manual data capture, they have limited capacity to resolve plant architecture at high resolution. For example, leaf area measurements are affected by blade curvature, leaf angle and movement, making accurate estimations of plant growth challenging using 2D [9,22]. Several 3D imaging methods have been developed that overcome some of the limitations of 2D. These can be classified as passive and active 3D imaging approaches and are briefly outlined below.

Passive 3D imaging approaches capture plant architecture without introducing new energy (e.g. light) into the environment [23]. Methods and technologies using this approach include multi-

view stereo [24,25], of which the most common is binocular stereo [26,27], structure from motion [28], light-field (plenoptic) cameras [29], and space carving approaches [30]. Passive approaches that use two or more sensors, or have moving parts (e.g. robot arm or gantry systems), often encounter difficulties in identifying and aligning the same points in different images (i.e. the so called 'correspondence problem'), which can result in imprecise reconstruction of 3D shapes [31]. Plant leaves and canopies can be particularly challenging as they often represent large homogenous areas with little salient texture. Imprecise 3D reconstructions can be smoothed, but at the expense of plant surface detail [32]. Space carving overcomes the correspondence problem, but requires many different views of an object and may still fail to reconstruct crowded areas (e.g. overlapping leaves) [30]. To our knowledge, only light-field cameras have been utilised successfully for capturing 3D plant growth throughout the diel (day-night) cycle [29,33]. However, light-field systems rely on expensive camera technology to capture high-resolution data, and like other passive approaches, require consistent and favourable lighting conditions.

Active 3D imaging approaches emit energy (e.g. light), which can overcome several problems associated with passive approaches. Structured light [34] and laser scanners [35–37] are active technologies that rely on triangulation to determine the point locations in a 3D space. Both methods can provide high-quality 3D reconstructions of plant canopy architecture, but structured light approaches require very accurate correspondence between images while laser scanners can be slow, and can potentially heat or even damage plants at high frequencies [38]. Furthermore, triangulation techniques are susceptible to occlusions (e.g. other objects in the environment or leaf overlap) that can reduce data quality. Time of Flight (ToF) cameras (e.g. LiDAR) comprise another active 3D imaging approach that determine the distance of a point directly from the time delay between an emitted light pulse and its reflection. However, the resolution of ToF cameras is still relatively low, which has tended to limit its use to imaging

larger plants [39,40]. Although both passive and active 3D imaging approaches can significantly improve the accuracy of plant growth measurements and even expand on the architectural traits available to capture compared to 2D imaging, existing 3D imaging techniques still lack in several crucial areas such as speed, availability, portability, spatial resolution and cost [18].

Photometric Stereo (PS) is an active imaging technique that is low-cost and can achieve high image resolutions and fast capture speeds [41]. This approach has been applied only recently to plant phenotyping and has shown significant promise [42]. PS relies on a set of images of an object captured under controlled, varied and directional illumination (Fig. 1; Supplementary Information S1). The obtained images are then used to generate a dense surface normal (SN) map of matching resolution, where each pixel represents a normal vector's components (i.e. the orientation in three cardinal directions - x, y and z) that allows the overall orientation of the object to be determined. Prior work has shown that plant leaf SN data acquired by PS can be captured at very high resolutions, and thus has significant advantages in encoding complex 3D morphology to aid challenging automated recognition and quantification tasks, such as the extraction of plant growth data [42,43].

Machine learning is now emerging as a promising field to transform the automation of trait extractions from plant image data sets [44,45]. Work in the model plant *Arabidopsis thaliana* (hereafter Arabidopsis) has revealed much about the molecular processes underlying the relationship between biomass and yield [46], and several methods have been developed for automating data extraction from Arabidopsis images [47]. Recently, significant advances have been made in the development of artificial neural networks (NNs) for automated segmentation of the rosette and individual leaves, and leaf counting using 2D image data [48–50]. However, the performance of NN approaches for leaf segmentation, for example, are still limited by a need for large annotated data sets for training, as models trained with small-scale databases

typically generalise weakly. To our knowledge, currently there are no NN models optimised for leaf segmentation using 3D data. A subsequent challenge is accurate object tracking to enable segmented leaves to be tracked across different time points of a data set [51,52].

Here we present a novel, low-cost imaging system called PS-Plant that for the first time utilises PS for monitoring the growth and development of Arabidopsis in 3D. We compared the accuracy of 3D vs 2D data from PS-Plant for estimating leaf area, angle, and rosette growth against ground truth measurements and showed comparable results to the state-of-the-art 3D light-field camera and laser scanning systems [29,36,37]. To demonstrate the versatility of PS-Plant, we analysed growth under a matrix of different conditions that illustrate the dramatic effect of the environment on the 3D phenotype of a wild-type Arabidopsis plant. Furthermore, we showed that 3D data from PS-Plant can be used to train NN models for automated leaf segmentation of a growing rosette, as an important first step in extracting plant features. Finally, we demonstrated that utilisation of machine learning for leaf segmentation and PS data can be combined to extract useful growth traits related to dynamic leaf movement and rosette development.

Results and discussion

Photometric stereo imaging using PS-Plant provides accurate spatial data for Arabidopsis plants

PS-Plant consists of a machine vision camera surrounded by four or eight Near Infrared (NIR) Light Emitting Diodes (LEDs) and a bespoke LED controller that allows rapid switching of the LEDs for high temporal data acquisition (Fig. 1A-C; Supplementary Data S1). PS-Plant can acquire up to 40 2D images per second at a spatial resolution of 2048 × 2048 pixels. The acquisition process takes 125-225 milliseconds per set of PS images, followed by *ca*. 5 s to process the 2D images to compute SN map estimations and 3D surface integration

(Supplementary Information S1 and S2). A NIR filter positioned in front of the lens provides consistent contrast and brightness for images captured throughout the diel cycle. The camera provides a 17 x 17 cm field of view that allows simultaneous tracking of up to nine Arabidopsis plants in 5 x 5 cm pots. Growth data sets for individual plants were extracted from each master image experiment data set using a Python-based GUI software. Overall, PS-Plant is portable and light-weight (*ca*. 7 kg without a PC) and could be adjusted to fit in different growth environments including growth cabinets or greenhouse environments. At the time of manufacture, the total cost for PS-Plant was approximately US$3,200.

A key assumption in PS is that the surface of the imaged object should exhibit Lambertian reflectance (i.e. it reflects light equally in all directions, while the reflected intensity diminishes according to the Lambert's cosine law) (Supplementary Information S1) [41]. As the reflectance of the object deviates from the Lambertian model, the subsequent estimation error increases accordingly. To verify if PS-Plant could accurately estimate total area and angle of an object, we initially used rectangular flat pieces of acrylic of known area (600 mm^2) covered in white matte paper, which achieved a close approximation of Lambertian reflectance [53], and imaged with a black matte background to facilitate image segmentation [54]. The acrylic objects were placed on laser cut wedges to allow imaging at a range of known angles (Fig. 2A). The projected areas were estimated using 2D and 3D data obtained from PS-Plant. The 3D data enabled us to estimate the object inclination angles, which were compared to the ground truth (Fig. 2B). Using 3D data, the area was estimated accurately up to 45° with a Mean Relative Error (MRE) of 1.0% (see Supplementary Information S3 for formulas). In contrast, estimates based on 2D data became inaccurate at inclinations greater than 10°, with a MRE of 10.3% when all angles were considered. Angle estimations consistently matched the known angle for all inclinations tested with a Mean Absolute Error (MAE) of 0.89°. These results highlighted the accuracy of PS-Plant in estimating the angle and area of a flat object in 3D space.

Next, we investigated Arabidopsis rosettes in PS-Plant and observed that Arabidopsis leaves exhibited near Lambertian reflectance under NIR light (Supplementary Information S1). We hypothesised that longer wavelengths penetrate deeper into the leaf and are then typically scattered, rather than specularly reflected at the leaf surface [55,56]. Similarly to the object area and angle estimation experiment, we imaged Arabidopsis rosettes inclined from 0° to 45° using a rotary inclination table and compared the estimated areas using 2D and 3D data with ground truth measurements of the imaged rosettes (Fig. 2C). Even without inclination (i.e. at 0°) estimates based on 3D data were more accurate compared to those from 2D data, indicating that the former was more capable of approximating areas for complex objects that include a degree of surface topographic relief (e.g. an Arabidopsis rosette). 3D data continued to outperform 2D data at increased inclinations with a MRE of 4.5% and 18.1% for 3D and 2D estimations, respectively. The accuracy of 3D estimations did decrease at angles >30° due to the increase in leaf (self-) occlusion that occurred when the whole rosette was inclined (Supplementary Information S4). When the accuracy of angle estimations was tested with selected individual leaves from the Arabidopsis rosettes (Fig. 2D), PS-Plant achieved a MAE of 3.8° for leaf angle estimations. We observed that the estimated and known leaf inclination angle correlated in the mid-range (10 to 30°) but less so at lower and higher angles. This was likely due to the natural curvature of Arabidopsis leaves compared to a flat surface, as Arabidopsis leaf blades typically have a convex shape when observed from above. Therefore, when the leaves were not inclined (i.e. at 0°), the estimated angles were still higher than zero as they were calculated from the varying SN values across each leaf blade surface.

PS-Plant enables accurate 3D reconstructions of growing Arabidopsis rosettes

Following validation, we assessed the accuracy and consistency of PS-Plant in monitoring plant growth and mean rosette inclination over time (Fig. 3). PS-Plant captured both 2D and 3D data

for Arabidopsis plants for 12 days, starting from 11 days after germination (DAG) in standard growth conditions (22 °C, 150 µmol photons m^{-2} s^{-1}, 12 : 12 hr light : dark). The automated image capture program resulted in a SN map produced for each plant every 30 minutes that was used to characterise rosette surface curvature (Fig. 3A). Furthermore, SN data could be used to derive rosette surface inclination angles and concavity/convexity values. Information on leaf curvature can be used, for example, in leaf developmental analysis to evaluate perturbances in normal leaf abaxial / adaxial expansion [57,58].

Both 2D and 3D data sets produced exponential growth curves for projected rosette area (PRA) that were typical for Arabidopsis growth (Fig. 3B). However, 2D data consistently underestimated PRA and showed erroneous reductions in area estimates consistent with rhythmic nastic leaf movements (Fig. 3C, D; Supplementary Data S2). In contrast, 3D data accounted for leaf curvature and movement, such that PRA increased more smoothly over the time course of the experiment. The small decreases observed for PRA from 3D data were associated with self-occlusion at high leaf inclination angles (as in Fig. 2). A number of studies have shown that growing Arabidopsis leaves exhibit rhythmic movement that is controlled by the circadian oscillator [59–62]. PS-Plant estimations of rosette surface inclination (i.e. the total inclination of all rosette leaf blades and petioles) is able to accurately record this rhythmicity, which in our 12L:12D conditions achieved an amplitude peak at 4-6 hr post dusk (Fig. 3D) (calculated using BioDare2; see Materials and Methods). Interestingly, our data showed that leaf rhythmicity appears to be anticipatory up to 16 DAG, after which it was strictly diurnal. As older plants have a higher proportion of mature leaves, that are no longer elongating, our data suggests that these leaves still exhibit rhythmic movements but they are driven by the daily light-dark cycle rather than the circadian oscillator. These data highlight the capability of PS-

Plant to not only provide accurate area estimates, but to capture leaf movement rhythms that are regulated by the circadian clock and the prevailing photoperiod.

Rosette architectural parameters derived from 2D data were also obtained from PS-Plant, including circularity (or stockiness), compactness, diameter and perimeter (Fig. 3E-H) [29,63,64]. These data showed, for example, an increase in perimeter and diameter that was consistent with plant growth, and a decrease in compactness, which was associated with elongation of leaf petioles as the rosette developed.

PS-Plant reveals 3D growth traits for Arabidopsis plant grown in different environments

We next wanted to establish whether PS-Plant could capture alterations in growth plasticity induced by changes in the external light and temperature environment. Low levels of photosynthetic active radiation are known to elicit a shade avoidance response (SAR), where plants exhibit elongated stems and petioles, increased hyponasty and smaller and fewer leaves [65–67]. As high temperatures to some extent target the same molecular pathways, heat also elicits an SAR-type response [68,69]. These studies illustrate that the growth strategy adopted by the plant is strongly dependent on the surrounding light environment and the ambient temperature. To capture these morphological changes we tracked Arabidopsis plants under nine conditions that differed in temperature [17 (LT), 22 (LT) and 27°C (HT)] and light intensity [40 (LL), 150 (ML) and 300 µmol photons m^{-2} s^{-1} (HL)] (Fig. 4A; Supplementary Fig. S1; Data S3).

Plants grown in LL had small leaves, recorded as low PRA, which was comparable in plants grown at different temperatures. Increases in light levels led to a concomitant rise in PRA, however, over light intensities of 150 µmol m^{-2} s^{-1} the PRA was strictly temperature-dependent with the highest PRA achieved at the highest light and temperature (Fig. 4B). The observed

differences in PRA were reasonably consistent with overall biomass accumulation at 24 DAG (Fig. 4C, D). Notably, in ML plants a shift from 17°C to 22°C led to an increase in biomass, while a shift from 22°C to 27°C did not, suggesting that the observed increase in PRA at 27°C was a result of cell expansion rather than division. HL and ML plants produce more leaves at 22°C compared to 17°C, signifying a larger investment in vegetative growth. Plants grown at 27°C induced flowering in HL and ML plants and so their final leaf number was slightly lower than at 22°C (Fig. 4E).

Together, these results could be explained by the thermodynamic relationship between the dark reactions (e.g. Rubisco activity and the Calvin cycle) and light reactions of photosynthesis. The assimilation rate of CO_2 by Rubisco is temperature-dependent, such that increased temperatures (up to *ca*. 30 °C) typically correlate with increased CO_2 assimilation in C3 plants grown under non-limiting light conditions [70–72]. These photochemical processes most likely underlie the light- and temperature-dependent changes in PRA and investment in leaf biomass production. Contrasting with this, in LL the supply of ATP and NADPH to the Calvin cycle by the light reactions may have constrained CO_2 uptake, and thus growth rates were not improved by increased temperatures.

PS-Plant also captured differences in petiole length. Analysis of the ML and HL illustrated that increased temperature stimulated petiole elongation in these plants. This is evident in PS-Plant measurements of plant compactness. However, this data also show that HL plants are generally more compact than ML (Supplementary Fig. S2), and that temperature-mediated differences in compactness are less evident in plants grown in HL. This indicates that carbon resources are more readily allocated to leaf expansion rather than petiole growth under higher light intensities.

We then compared the relative expansion rate (RER) based on 3D PRA data for different light-temp conditions over the diel cycle (Fig. 4F-H). RER data for Arabidopsis vary between

different studies, but generally have comparable rates within light and dark periods for wild-type plants grown under standard growth conditions [9,29,36,73]. In the present study, RER in the dark period was not significantly different across all growth conditions tested [as determined by one-way ANOVA ($p < 0.05$), followed by Tukey's HSD tests]. This was not unexpected, as the rate of leaf starch turnover during the night is known to be maintained over a wide range of environmental conditions and temperatures in Arabidopsis [74,75]. RER values during the light period were comparable for plants grown in ML and plants grown in HL-MT and HL-LT. In contrast, HL-HT plants showed an increase RER in the light compared to the dark period. As HL-HT plants also had the highest biomass accumulation (Fig. 4D), results obtained with PS-Plant suggest HL-HT plants were limited more by carbon turnover than CO_2 assimilation. All plants grown in LL had a significantly decreased RER in the light compared to the dark period. Notably, temperature had no impact on RER in the light for LL plants, indicating that photosynthetic growth was primarily limited by the low irradiance.

The internal circadian clock in plants has a periodicity close to 24 hr that can be entrained by environmental cues [76]. Thus, we next used PS-Plant to examine the rhythmicity of total leaf movement (i.e. rosette surface inclination, see Fig. 3D) to compare the capacity of entrainment of the clock to different growth conditions (Fig. 5A-C; Supplementary Fig. S3) [59]. We compared three standard parameters: period, phase and amplitude [76,77].

As expected, all conditions showed a similar period for leaf movement of *ca*. 24 hr ($p < 0.05$) as all plants were grown in a 12 : 12 hr light : dark cycle (Fig. 5D). However, phase and amplitude differed between growth conditions. Through all conditions peak phase occurred during the night, with the general observation that incremental rises in light intensity led to a phase delay in the peak. A possible exception is that in 17°C HL rhythms peaked at the end of the day. It is noteworthy that the 17°C ML and HL leaf rhythm traces are very low amplitude, most likely because these plants had very limited petiole growth. We also found that

temperature effects the phase of the rhythm across all light conditions. For example, in both ML and HL growth at 27°C advanced the peak phase compared to 22°C.

Monitoring plant behaviour through time revealed the impact on light and temperature through development (Supplementary Fig. S3). A common trend is that warm temperatures increase mean rosette leaf inclination angle, or hyponasty, though the threshold for this response varies in the different light treatments. Another notable feature is that hyponasty and rhythm amplitude dampen over time. Our data show that under LL the leaf movement rhythms are more sinusoidal and higher amplitude rhythms than in ML and HL. Leaf movement rhythm waveforms of ML and HL are also quite different from LL, with some evidence of tracking dawn and dusk. Interestingly in HL the rhythm at 17°C is clearly in antiphase with 22°C and 27°C. Through time the 17°C rhythm dampens to high leaf hyponasty, while 22°C/ 27°C leaf rhythms dampen to a low leaf angle. In both cases this effect appears to arise from a gradual reduction in rhythmic regulation during the night period. Overall, these data illustrate that PS-Plant is able to extract quantitative data on a large range of traits associated with rhythmic leaf growth that are typically challenging to capture.

Use of PS-Plant data and machine learning for accurate leaf segmentations

Our next goal was to examine the capacity of PS-Plant to track the phenotypic behaviour of individual leaves on a growing Arabidopsis rosette. To achieve this, we labelled individual leaves in 221 images of ML-MT rosettes (Supplementary Information S5) and used machine learning approaches to segment leaves. We compared two available NN architectures, RNN with recurrent attention (RNN) [49] and Mask R-CNN (R-CNN) [78], to examine the suitability of PS-Plant data for NNs designed for instance segmentation using RGB images. We focused on ML-MT plants as their growth was more uniform across different individuals compared to other growth conditions, which allowed the models to converge faster and achieve

better results during the training process. The data set was split into 179 and 42 images (approx. 80: 20 ratio) for training and validating the models, respectively. To avoid overfitting the model, we manually selected plant images for training and validation data sets to ensure that all time-series images of a single specimen appear in either training or validation data sets, but not both.

PS-Plant produces a range of different data: from grayscale images to SN maps (e.g. Fig. 3). We trained the RNN and R-CNN architectures from initial random weights, while R-CNN was also pre-trained using transfer learning weights generated using the COCO data set [79]. The RNN and R-CNN architectures were trained using three different types of PS data to compare for segmentation accuracy: i) composite (SN in x and y direction, and albedo for RGB layers), ii) grayscale, and iii) albedo images. All data used for training, including the raw PS-Plant data and rosette masks are available as outlined Supplementary Information S5. The obtained leaf segmentations were compared to the ground truth images using symmetric best dice (SBD; score of the accuracy of leaf instance segmentation) and foreground-background dice (FBD; score of the accuracy of rosette segmentation) evaluation formulas (Supplementary Information S3) [47].

The type of PS data used did not significantly influence SBD or FBD scores, suggesting that accuracy of RGB-based models was not affected by the different types of PS-based data. The most accurate leaf segmentation results were achieved with models based on the R-CNN architecture using pre-trained weights (Fig. 6; Table 1), resulting in SBD scores that ranged from 0.806 (composite image) to 0.814 (albedo). In comparison, the RNN architecture resulted in lower SBD scores of 0.440 (composite image) and 0.560 (albedo and grayscale). The pre-trained R-CNN model also achieved the most accurate rosette segmentation results, with FBD scores ranging from 0.94 (albedo) to 0.946 (grayscale). In contrast, FBD scores for the RNN model varied from 0.798 (composite image) to 0.891 (albedo), indicating that the relative

performance of the RNN architecture was worse for both leaf and rosette segmentation with our data sets when compared to the R-CNN approach.

Table 1. Performance comparison of leaf instance segmentation for two different machine learning architectures.

	Mask R-CNN				RNN	
Image type	Random weights		Pre-trained weights		Random weights	
	SBD	FBD	SBD	FBD	SBD	FBD
Grayscale	0.813	0.942	0.812	0.946	0.556	0.866
Albedo	0.758	0.913	0.814	0.940	0.560	0.891
Surface normal map	0.789	0.922	0.806	0.941	0.440	0.798

The Mask R-CNN [78] and RNN with recurrent attention [49] architectures were trained with composite (SN in x and y direction, and albedo for RGB layers), grayscale or albedo images. The Mask R-CNN was trained both from initial random weights and from pre-trained model weights, while RNN was only trained from initial random weights. Abbreviations: SBD, Symmetric best dice; FBD foreground-background dice.

Using PS-Plant data for dynamic tracking of individual leaf growth and movement

We next investigated the performances of four different approaches for tracking leaves using the segmented image data sets (e.g. Fig. 6): i) kernelized correlation filters [80], ii) optical flow [81], iii) multiple instance learning tracker [82], and iv) a particle filter [83]. Object tracking, especially with partially or even completely occluded objects, is one of the most challenging areas in computer vision [51,52]. Tracking Arabidopsis leaves over time is particularly challenging due to changes in both shape and movement during growth together with

associated occlusions (Supplementary Information S4). The best results were achieved with a particle filter based on leaf instance centroid location and velocity across the time-series images (Fig. 7). Leaf overlap remained a limitation, as an occluding leaf was sometimes assigned the label of an occluded leaf. However, erroneous labelling was found to be infrequent and straightforward to manually corrected *post hoc*, resulting in a robust semi-automated leaf tracker (Supplementary Data S4).

Once we were confident that we could reliably track individual leaves using PS-Plant, we separated leaf blades and petioles by applying a morphological opening function with a predefined radius (3 to 11 pixels based on the leaf size) to the leaf binary mask. The point of differentiation (P_B) is the mean x and y coordinates of the leaf blade and petiole (Fig. 8A). This enabled separate examinations of leaf blade and petiole traits. We then derived separated tissue-specific data including leaf blade area and inclination angle, and leaf blade and petiole length. The angle of leaf blade inclination was estimated using two different methods: i) a point-based approach where leaf blade angle was determined using SN data across the line from P_B to the leaf tip (P_T), and ii) the mean surface inclination of the whole leaf blade. Both methods produced similar results (Supplementary Fig. S4). However, we chose to use the latter (ii) as the P_B was not always visible due to leaf occlusions or the petiole being too small to be distinguished (e.g. maturing leaves or leaves grown in low temperature).

To demonstrate our approach, we tracked leaves 1 to 4 of plants grown in ML at three different temperatures from 15 to 18 DAG. Leaves 1 to 4 were chosen as representative examples of maturing (1 and 2) and immature (3 and 4) leaves (Fig. 8; Supplementary Data S5A-D). Consistent with our findings for PRA under different growth conditions (Fig. 4; Supplementary Fig. S1), the leaf blade areas of maturing and immature leaves from HT plants were significantly larger than leaf blades from MT and LT plants [as determined by one-way ANOVA ($p < 0.05$), followed by Tukey’s HSD tests; Fig. 8B]. The latter results confirmed that

the increased PRA observed using PS-Plant for plants grown in HT plants was specifically associated with an increase in leaf blade area. Leaves that emerged prior to the start of the experiment at 11 DAG (i.e. leaf 1) showed an increase in leaf blade area in HT plants compared to MT and LT plants (Fig. 8B). However, leaves that emerged after 11 DAG (i.e. leaf 4) had an even more dramatic growth response to increased temperatures. For example, the blade area for leaf 1 and 4 at 17 DAG was 40% and 130% higher in HT compared to LT, respectively. Similarly, the mean surface inclination of leaf blades was higher in HT (Fig. 8C). The latter result was also consistent with our findings for whole rosette surface inclination at higher temperatures (Fig. 3; 5; Supplementary Fig. S3).

We then calculated parameters associated with diurnal movement for individual leaf blades (Fig. 8D). We targeted immature leaf blades as their movement patterns were clearer and more consistent compared to maturing leaf blades. Period or phase measurements from immature leaf blades were generally similar between growth conditions and comparable to values for whole rosettes (Fig. 5). In contrast, measurements of immature leaf blade amplitude were significantly enhanced at MT and HT and generally higher than values for whole rosettes. This was not unexpected as immature leaves are more active than older leaves and contribute more to overall whole rosette amplitude (see Supplementary Data S3 and S5). Furthermore, the observed temperature-associated increases in amplitude and leaf hyponasty were consistent with whole rosette data (Fig. 5D; Supplementary Fig. S3B). Thus, we concluded that measurements of periodic rhythms can be performed reliably with PS-Plant data using whole rosettes or individual leaf blades. The values obtained in the present study for period and phase are comparable to those reported for wild-type plants under standard growth conditions by other automated top-down systems for monitoring leaf movement, such as OSCILLATOR [84].

Finally, we used PS-Plant to reveal whether petiole elongation showed a similar response to temperature as the leaf blade by comparing the ratio of leaf blade and petiole length from maturing and immature leaves (Fig. 8E). Petioles have been shown to elongate faster at higher temperatures [68,85,86]. In the present study we observed that leaves from MT and LT plants had a blade: petiole length ratio that ranged from 2: 1 to 4: 1. Immature leaves did not have a detectable petiole under LL, thus only maturing leaves were included at LT. In contrast, HT plants had ratios of approximately 1: 1 for both maturing and immature leaves, indicating that HT resulted in an increase petiole elongation relative to leaf blade growth under ML. Future work should examine this ratio at different light intensities, as petioles and leaf blades are known to have different responses to light. For example, petioles are known to elongate faster under low light while leaf blades grow more slowly [69,87].

Conclusion

In this paper, we have introduced an adaptable and low-maintenance platform for affordable, advanced image-based phenotyping. A key goal was to ensure accessibility to the research community. In this regard, PS-Plant can be considered a powerful, alternative solution to 3D systems based on laser scanning and light-field camera technologies [29,36], which is particularly well suited for setup in low-income or developing countries. Our system exploits the richer data provided by PS-Plant with a combination of traditional image processing and machine learning techniques to extract rosette and leaf-level measurements in an automated manner. Here, we have demonstrated that PS-Plant is able to accurately monitor several growth traits and diurnal rhythms of different phenotypes of Arabidopsis plants produced in response to varied environments. This provides credibility that future work with PS-Plant will produce robust data for a wide variety of mutant phenotypes. Additionally, the concomitant quantification of overall growth, leaf traits and circadian rhythms can facilitate a better

understanding of the relationships among environment, plant yield and internal molecular networks. Previous work has also highlighted that PS can capture high-resolution 3D surface details of leaf surface structures, such as leaf curvature and trichomes, which could be used to investigate dynamic changes in leaf development [43]. Research in plant phenotyping needs to focus on increasing accessibility and instituting effective data standards and management practices to assist with improving plant productivity and genetic gain [88,89]. To help accelerate the latter, we have provided the PS imaging data set from this study for community access (Supplementary Information S5). Future work with PS-Plant will focus on improvements in leaf tracking [90], integration with spectral information [91], and incorporation of a low-cost depth camera to combine the high resolution of PS with a lower resolution depth map to characterise whole plants with more complex architectures.

Materials and Methods

Plant materials

Arabidopsis (Arabidopsis thaliana (L.) Heynh. Col-0) wild-type seeds were stratified for 2-3 days at 4 °C. Each seed was placed in a square pot (50 mm) containing F2+S compost (Levington) covered in acrylic black felt fabric with a central hole (5 mm) and germinated at 22 ºC under white light (150 µmol photons m^{-2} s^{-1} at the plant level) in 12 : 12 hr light : dark for 10 d in a Percival growth cabinet (SE- 41AR2). For the plant area validation experiment, the plants were kept in this cabinet for 22 DAG. For imaging with PS-Plant, the seedlings were transferred to a Snijders growth cabinet (Microclima MC1000).

PS-Plant hardware

PS-Plant consists of a machine vision NIR monochrome camera (Grasshopper3 GS3-U3-41C6NIR-C, FLIR Systems Inc., Canada) with a 16 mm fixed focal length lens (Kowa 1"SC

LM25SC, Kowa Company Limited, Japan) with a NIR filter attached (LP920, MidOpt, Illinois, USA), four or eight NIR LEDs (PowerStar IR 940 nm, Intelligent LED Solutions, UK), and an in-house designed LED controller that allows rapid switching of LEDs using an Arduino platform (MKRZero, Arduino, Italy). The camera and LEDs were fixed on a square acrylic sheet (44 × 44 cm) and positioned at a height of 40 cm above the imaging plants (Fig. 1B, C). The camera was positioned centrally in the sheet and the LEDs were positioned around the camera at 45° angle increments. The LEDs were tilted at a 30° angle to illuminate the area under the camera field of view (Fig. 1B). The base of the rig was painted matt black to limit the introduction of specularities from the background. A PC laptop (K501UQ-DM050T, AsusTek Computer Inc., Taiwan) was used to control LED illuminations, and acquire, store and process images using GUI software written in Python. Details on rig assembly and the LED controller design are outlined in Supplementary Information S2.

Leaf movement rhythm analysis

The leaf movement rhythm analysis was performed using the mean inclination angles (whole rosette or individual leaf blade) as an input for BioDare2 beta (https://biodare2.ed.ac.uk/). The data was treated with baseline detrending prior to period, phase and amplitude estimations, which was done using the MFourFit algorithm [77].

AVAILABILITY OF SUPPORTING DATA

The data set supporting the results of this article is available in an Edinburgh DataShare repository https://datashare.is.ed.ac.uk/handle/10283/3200 and outlined in Supplementary Information S5. This data set represents approximately 0.4% of the 50,625 images captured during the “matrix” growth experiment (see Fig. 4A).

ADDITIONAL FILES

Supplementary Information S1. Overview of 2D image data processing captured using PS-Plant.

Supplementary Information S2. Overview of the PS-Plant hardware.

Supplementary Information S3. Formulas.

Supplementary Information S4. Area estimation errors.

Supplementary Information S5. PS-Plant data set description.

Supplementary Figure S1. Rosette and individual leaf growth analysis.

Supplementary Figure S2. Rosette compactness for plants grown in different conditions.

Supplementary Figure S3. Mean rosette surface inclinations for all growth conditions separated by light treatment.

Supplementary Figure S4. Estimated leaf inclination of leaf 1 in medium light and 27°C.

Supplementary Data S1. Interactive 3D model of the PS-Plant system. The model is provided as an .stl file (Rich Media 1.stl), an online link to zoomable, colour version can be found here: https://sketchfab.com/models/eaccbca9ffa946d6891719bca640c14e.

Supplementary Data S2. Comparison of Arabidopsis growth from 2D and 3D data. The graph (top) includes standard deviation of PRA data for three plants growing under conditions outlined in Fig. 3. Examples of plant growth are shown below for 2D [albedo; bottom left (see Supp. Info. 1 for details)] and surface normal map data (bottom right).

Supplementary Data S3. Arabidopsis plants grow and move differently under different light and temperature conditions. Examples of surface normal models for plants of the same age under each growth conditions tested (see Fig. 4) are shown from 11 to 24 DAG.

Supplementary Data S4. Automated tracking of individual Arabidopsis leaves. Example of leaf label tracking following rosette segmentation of a ML-MT plant shown from 15 to 18 DAG. Note that leaves retained the same colour after tracking (right).

Supplementary Data S5. Using PS-Plant for automated tracking of individual Arabidopsis leaf movement in 3D. Four videos illustrate leaf blade tracking of leaves 1 to 4, respectively, for a plant grown in ML-MT from 15 to 18 DAG. Each video shows a trail of leaf blade centroid movement (red dots) on an albedo 2D video (top left). Blue dots illustrate leaf blade movement on 2D x-y (bottom left) and y-z projections (bottom right), and a 3D x-y-z graph (top right).

DECLERATIONS

List of abbreviations

2D: two dimensional; 3D: three dimensional; ANOVA: analysis of variance; AGV: automated ground vehicles; COCO: common objects in context database; DAG: days after germination; FBD: foreground-background dice score; GUI: graphical user interface; HL: high light; HSD: Tukey's honest significant difference test; HT: high temperature; LED: light emitting diode; LiDAR: distance measurement method using pulsed laser light; LL: low light; LT: low temperature; MAE: mean absolute error; ML: medium light; MRE: mean relative error; MT: medium light; NIR: near-infrared; NN; neural network; P_B: leaf base point, or intersection point between leaf blade and petiole; PC: personal computer; P_O: rosette origin point; PRA: projected rosette area; PS: photometric stereo; P_T: leaf tip point; R-CNN: short for Mask R-CNN NN architecture; RER: relative expansion rate; RGB: red, green and blue channels, or a colour image; RNN: short for end-to-end instance segmentation with recurrent attention NN architecture; SAR: shade avoidance response; SBD: symmetric best dice score; SD: standard deviation; SN: surface normal; ToF: time of flight.

Consent for publication

This study abides by UK guidelines and legislation for plant science research.

Competing interests

The authors declare that they have no competing interests.

Funding

This work was supported by the UK Biotechnology and Biological Sciences Research Council grants BB/N02334X/1, BB/M025551/1 and BB/N005147/1. GB was funded by the University of the West of England (UWE) Partnership Fund.

Authors' contributions

GB, MFH and IJH designed the hardware and software of PS-Plant system including the image processing pipeline. AM, KJH and LCTS designed the plant experimental setup. GB and LCTS performed and analysed the validation experiments. LCTS performed and analysed plant growth experiments. GB designed the study for NN model generation for leaf segmentation. AM, LCTS and GB wrote the manuscript, with assistance from all authors. AM, LNS and MLS supervised the project.

Acknowledgements

We thank Jackie Aim and Stewart Cromar (University of Edinburgh) for assistance with the process of 3D model designs.

REFERENCES

1. Long SP, Marshall-Colon A, Zhu XG. Meeting the global food demand of the future by engineering crop photosynthesis and yield potential. Cell. 2015;161:56–66.

2. Meinke H. Agricultural impacts: Europe’s diminishing bread basket. Nat Clim Chang.

2014;4:541–2.

3. Chew YH, Wenden B, Flis A, Mengin V, Taylor J, Davey CL, et al. Multiscale digital Arabidopsis predicts individual organ and whole-organism growth. Proc Natl Acad Sci. 2015;112:E2556.

4. Elliott J, Deryng D, Müller C, Frieler K, Konzmann M, Gerten D, et al. Constraints and potentials of future irrigation water availability on agricultural production under climate change. Proc Natl Acad Sci. 2014;111:3239–44.

5. Cang FA, Wilson AA, Wiens JJ. Climate change is projected to outpace rates of niche change in grasses. Biol Lett. 2016;12:20160368.

6. Liang X-Z, Wu Y, Chambers RG, Schmoldt DL, Gao W, Liu C, et al. Determining climate effects on US total agricultural productivity. Proc Natl Acad Sci. 2017;114:2285–92.

7. Ahmad J, Sun J, Smith L, Smith M. Improving photometric stereo through per-pixel light vector calculation. Br Mach Vis Conf. 2013;1–12.

8. Furbank RT, Tester M. Phenomics - technologies to relieve the phenotyping bottleneck. Trends Plant Sci. 2011;16:635–44.

9. Dobrescu A, Scorza LCT, Tsaftaris SA, McCormick AJ. A “Do-It-Yourself” phenotyping system: Measuring growth and morphology throughout the diel cycle in rosette shaped plants. Plant Methods. 2017;13:1–12.

10. Shakoor N, Lee S, Mockler TC. High throughput phenotyping to accelerate crop breeding and monitoring of diseases in the field. Curr Opin Plant Biol. 2017;38:184–92.

11. Tardieu F, Cabrera-Bosquet L, Pridmore T, Bennett M. Plant Phenomics, From Sensors to Knowledge. Curr Biol. 2017;27:770–83.

12. Araus JL, Kefauver SC. Breeding to adapt agriculture to climate change: affordable phenotyping solutions. Curr Opin Plant Biol. 2018;1–11.

13. Sharma RC. Selection for biomass yield in wheat. Euphytica. 1993;70:35–42.

14. Richards RA. Selectable traits to increase crop photosynthesis and yield of grain crops. J Exp Bot. 2000;51:447–58.

15. Arora VK, Singh CB, Sidhu AS, Thind SS. Irrigation, tillage and mulching effects on soybean yield and water productivity in relation to soil texture. Agric Water Manag. 2011;98:563–8.

16. Zhang H, Flottmann S. Seed yield of canola (Brassica napus L .) is determined primarily by biomass in a high- yielding environment. Crop Pasture Sci. 2016;67:369–80.

17. Zhang H, Flottmann S. Genotypic variation in the accumulation of water-soluble carbohydrate in canola and its potential contribution to seed yield in different environments. F Crop Res. 2016;196:124–33.

18. Vázquez-Arellano M, Griepentrog HW, Reiser D, Paraforos DS. 3-D imaging systems for agricultural applications—a review. Sensors (Switzerland). 2016;16.

19. Green JM, Appel H, Rehrig EM, Harnsomburana J, Chang JF, Balint-Kurti P, et al. PhenoPhyte: A flexible affordable method to quantify 2D phenotypes from imagery. Plant Methods. 2012;8:1–12.

20. Dhondt S, Gonzalez N, Blomme J, De Milde L, Van Daele T, Van Akoleyen D, et al. High-resolution time-resolved imaging of in vitro Arabidopsis rosette growth. Plant J. 2014;80:172–84.

21. Minervini M, Giuffrida M V., Perata P, Tsaftaris SA. Phenotiki: an open software and hardware platform for affordable and easy image-based phenotyping of rosette-shaped plants. Plant J. 2017;90:204–16.

22. Chen JM, Black TA. Defining leaf area index for non- flat leaves. Plant Cell Environ. 1992;15:421–9.

23. Bianco G, Gallo A, Bruno F, Muzzupappa M. A comparative analysis between active and passive techniques for underwater 3D reconstruction of close-range objects. Sensors

(Switzerland). 2013;13:11007–31.

24. Pound MP, French AP, Fozard JA, Murchie EH, Pridmore TP. A patch-based approach to 3D plant shoot phenotyping. Mach Vis Appl. 2016;27:767–79.

25. Pound MP, French AP, Murchie EH, Pridmore TP. Automated Recovery of Three-Dimensional Models of Plant Shoots from Multiple Color Images. Plant Physiol. 2014;166:1688–98.

26. Biskup B, Scharr H, Schurr U, Rascher U. A stereo imaging system for measuring structural parameters of plant canopies. Plant, Cell Environ. 2007;30:1299–308.

27. Burgess AJ, Retkute R, Pound MP, Mayes S, Murchie EH. Image-based 3D canopy reconstruction to determine potential productivity in complex multi-species crop systems. Ann Bot. 2017;119:517–32.

28. Jay S, Rabatel G, Hadoux X, Moura D, Gorretta N. In-field crop row phenotyping from 3D modeling performed using Structure from Motion. Comput Electron Agric. 2015;110:70–7.

29. Apelt F, Breuer D, Nikoloski Z, Stitt M, Kragler F. Phytotyping^{4D}: A light-field imaging system for non-invasive and accurate monitoring of spatio-temporal plant growth. Plant J. 2015;82:693–706.

30. Gibbs J, Pound M, French A, Wells D, Murchie E, Pridmore T. Plant Phenotyping: An Active Vision Cell for Three-Dimensional Plant Shoot Reconstruction. Plant Physiol. 2018;1–26.

31. Tippetts B, Lee DJ, Lillywhite K, Archibald J. Review of stereo vision algorithms and their suitability for resource-limited systems. J Real-Time Image Process. 2016;11:5–25.

32. Xiong X, Yu L, Yang W, Liu M, Jiang N, Wu D, et al. A high-throughput stereo-imaging system for quantifying rape leaf traits during the seedling stage. Plant Methods. 2017;13:1–17.

33. Apelt F, Breuer D, Olas JJ, Annunziata MG, Flis A, Nikoloski Z, et al. Circadian, Carbon, and Light Control of Expansion Growth and Leaf Movement. Plant Physiol. 2017;174:1949–68.

34. Nguyen TT, Slaughter DC, Max N, Maloof JN, Sinha N. Structured light-based 3D reconstruction system for plants. Sensors (Switzerland). 2015;15:18587–612.

35. Paulus S, Schumann H, Kuhlmann H, Léon J. High-precision laser scanning system for capturing 3D plant architecture and analysing growth ofcereal plants. Biosyst Eng. 2014;121:1–11.

36. Dornbusch T, Michaud O, Xenarios I, Fankhauser C. Differentially Phased Leaf Growth and Movements in Arabidopsis Depend on Coordinated Circadian and Light Regulation. Plant Cell. 2014;26:3911–21.

37. Dornbusch T, Lorrain Ś, Kuznetsov D, Fortier A, Liechti R, Xenarios I, et al. Measuring the diurnal pattern of leaf hyponasty and growth in Arabidopsis a novel phenotyping approach using laser scanning. Funct Plant Biol. 2012;39:860–9.

38. Paulus S, Eichert T, Goldbach HE, Kuhlmann H. Limits of active laser triangulation as an instrument for high precision plant imaging. Sensors (Switzerland). 2014;14:2489–509.

39. Herrero-Huerta M, Lindenbergh R, Gard W. Leaf Movements of Indoor Plants Monitored by Terrestrial LiDAR. Front Plant Sci. 2018;9:189.

40. Thapa S, Zhu F, Walia H, Yu H, Ge Y. A novel LiDAR-Based instrument for high-throughput, 3D measurement of morphological traits in maize and sorghum. Sensors (Switzerland). 2018;18:1–14.

41. Woodham RJ. Photometric Method For Determining Surface Orientation From Multiple Images. Opt Eng. 1980;19:139–44.

42. Smith LN, Zhang W, Hansen MF, Hales IJ, Smith ML. Innovative 3D and 2D machine vision methods for analysis of plants and crops in the field. Comput Ind. 2018;97:122–31.

43. Zhang W, Hansen MF, Smith M, Smith L, Grieve B. Photometric stereo for three-dimensional leaf venation extraction. Comput Ind. 2018;98:56–67.

44. Singh A, Ganapathysubramanian B, Singh AK, Sarkar S. Machine Learning for High-Throughput Stress Phenotyping in Plants. Trends Plant Sci. 2016;21:110–24.

45. Pound MP, Atkinson JA, Townsend AJ, Wilson MH, Griffiths M, Jackson AS, et al. Deep machine learning provides state-of-the-art performance in image-based plant phenotyping. Gigascience. 2017;6:1–10.

46. Gonzalez N, Beemster GT, Inzé D. David and Goliath: what can the tiny weed Arabidopsis teach us to improve biomass production in crops? Curr Opin Plant Biol. 2009;12:157–64.

47. Scharr H, Minervini M, French AP, Klukas C, Kramer DM, Liu X, et al. Leaf segmentation in plant phenotyping: a collation study. Mach Vis Appl. 2016;27:585–606.

48. Aich S, Stavness I. Leaf counting with deep convolutional and deconvolutional networks. Proc - 2017 IEEE Int Conf Comput Vis Work ICCVW 2017. 2017;22–9.

49. Ren M, Zemel RS. End-to-End Instance Segmentation with Recurrent Attention. IEEE Conf Comput Vis Pattern Recognit. 2016. p. 21–6.

50. Ubbens J, Cieslak M, Prusinkiewicz P, Stavness I. The use of plant models in deep learning: An application to leaf counting in rosette plants. Plant Methods. 2018;14:1–10.

51. Yang H, Shao L, Zheng F, Wang L, Song Z. Recent advances and trends in visual tracking: A review. Neurocomputing. 2011;74:3823–31.

52. Smeulders AWM, Chu DM, Cucchiara R, Calderara S, Dehghan A, Shah M. Visual tracking: An experimental survey. IEEE Trans Pattern Anal Mach Intell. 2014;36:1442–68.

53. Azhar F, Emrith K, Pollard S, Smith M, Adams G, Simske S. Testing the Validity of Lamberts Law for Micro-scale Photometric Stereo Applied to Paper Substrates. 2015;246–53.

54. Otsu N. A threshold selection method from gray-level histograms. IEEE Trans Syst Man

Cybern. 1979;9:62–6.

55. Jacquemoud S, Baret F. PROSPECT: A model of leaf optical properties spectra. Remote Sens Environ. 1990;34:75–91.

56. Chelle M. Could plant leaves be treated as Lambertian surfaces in dense crop canopies to estimate light absorption? Ecol Modell. 2006;198:219–28.

57. Karidas P, Challa KR, Nath U. The tarani mutation alters surface curvature in Arabidopsis leaves by perturbing the patterns of surface expansion and cell division. J Exp Bot. 2015;66:2107–22.

58. Sandalio LM, Rodríguez-Serrano M, Romero-Puertas MC. Leaf epinasty and auxin: A biochemical and molecular overview. Plant Sci. 2016;253:187–93.

59. Edwards KD, Millar AJ. Analysis of circadian leaf movement rhythms in Arabidopsis thaliana. Methods Mol Biol. 2007;362:103–13.

60. Mizoguchi T, Wheatley K, Hanzawa Y, Wright L, Mizoguchi M, Song H, et al. LHY and CCA1 Are Partially Redundant Genes Required to Maintain Circadian Rhythms in Arabidopsis. Dev Cell. 2002;2:629–41.

61. Doyle MR, Davis SJ, Bastow RM, McWatters HG, Kozma-Bognár L, Nagy F, et al. The ELF4 gene controls circadian rhythms and flowering time in Arabidopsis thaliana. Nature. 2002;419:74–7.

62. Yoo SK, Hong SM, Lee JS, Ahn JH. A genetic screen for leaf movement mutants identifies a potential role for AGAMOUS-LIKE 6 (AGL6) in circadian-clock control. Mol Cells. 2011;31:281–7.

63. Jansen M, Gilmer F, Biskup B, Nagel KA, Rascher U, Fischbach A, et al. Simultaneous phenotyping of leaf growth and chlorophyll fluorescence via GROWSCREENFLUORO allows detection of stress tolerance in Simultaneous phenotyping of leaf growth and chlorophyll fl uorescence via GROWSCREEN FLUORO allows detection of stress tolera.

Funct Plant Biol. 2009;36:902–14.

64. De Vylder J, Vandenbussche F, Hu Y, Philips W, Van Der Straeten D. Rosette Tracker: An Open Source Image Analysis Tool for Automatic Quantification of Genotype Effects. Plant Physiol. 2012;160:1149–59.

65. Casal JJ. Shade Avoidance [Internet]. Arab. Book/American Soc. Plant Biol. 2012.

66. Pierik R, De Wit M. Shade avoidance: Phytochrome signalling and other aboveground neighbour detection cues. J Exp Bot. 2014;65:2815–24.

67. Gommers CMM, Keuskamp DH, Buti S, van Veen H, Koevoets IT, Reinen E, et al. Molecular Profiles of Contrasting Shade Response Strategies in Wild Plants: Differential Control of Immunity and Shoot Elongation. Plant Cell. 2017;29:331–44.

68. Quint M, Delker C, Franklin KA, Wigge PA, Halliday KJ, Van Zanten M. Molecular and genetic control of plant thermomorphogenesis. Nat Plants. 2016;2:1–9.

69. de Wit M, Ljung K, Fankhauser C. Contrasting growth responses in lamina and petiole during neighbor detection depend on differential auxin responsiveness rather than different auxin levels. New Phytol. 2015;208:198–209.

70. Berry J, Bjorkman O. Photosynthetic Response and Adaptation to Temperature in Higher Plants. Annu Rev Plant Physiol. 1980;31:491–543.

71. Bunce JA. Acclimation of photosynthesis to temperature in eight cool and warm climate herbaceous C3species: Temperature dependence of parameters of a biochemical photosynthesis model. Photosynth Res. 2000;63:59–67.

72. Yamori W, von Caemmerer S. Effect of Rubisco Activase Deficiency on the Temperature Response of CO2 Assimilation Rate and Rubisco Activation State: Insights from Transgenic Tobacco with Reduced Amounts of Rubisco Activase. Plant Physiol. 2009;151:2073–82.

73. Wiese A, Christ MM, Virnich O, Schurr U, Walter A. Spatio-temporal leaf growth patterns of Arabidopsis thaliana and evidence for sugar control of the diel leaf growth cycle.

New Phytol. 2007;174:752–61.

74. Smith AM, Stitt M. Coordination of carbon supply and plant growth. Plant, Cell Environ. 2007;30:1126–49.

75. Pyl E-T, Piques M, Ivakov A, Schulze W, Ishihara H, Stitt M, et al. Metabolism and Growth in *Arabidopsis* Depend on the Daytime Temperature but Are Temperature-Compensated against Cool Nights. Plant Cell. 2012;24:2443–69.

76. McClung CR. Plant Circadian Rhythms. Plant Cell Online. 2006;18:792–803.

77. Zielinski T, Moore AM, Troup E, Halliday KJ, Millar AJ. Strengths and limitations of period estimation methods for circadian data. PLoS One. 2014;9:1–26.

78. He K, Gkioxari G, Dollar P, Girshick R. Mask R-CNN. 2017 IEEE Int Conf Comput Vis. 2017. p. 2980–8.

79. Lin TY, Maire M, Belongie S, Hays J, Perona P, Ramanan D, et al. Microsoft COCO: Common objects in context. Lect Notes Comput Sci (including Subser Lect Notes Artif Intell Lect Notes Bioinformatics). 2014;740–55.

80. Henriques JF, Caseiro R, Martins P, Batista J. High-speed tracking with kernelized correlation filters. IEEE Trans Pattern Anal Mach Intell. 2015;37:583–96.

81. Lucas BD, Kanade T. An Iterative Image Registration Technique with an Application to Stereo Vision. Imaging Underst Work. 1981. p. 121–30.

82. Babenko B, Yang M-H, Belongie S. Visual Tracking with Online Multiple Instance Learning. Comput Vis Pattern Recognit. 2009;983–90.

83. Allan D, Caswell T, Keim N, van der Wel C. trackpy: Trackpy v0.3.2 [Internet]. Zenodo. 2016. Available from: http://doi.org/10.5281/zenodo.60550

84. Bours R, Muthuraman M, Bouwmeester H, van der Krol A. OSCILLATOR: A system for analysis of diurnal leaf growth using infrared photography combined with wavelet transformation. Plant Methods. 2012;8:1–12.

85. van Zanten M, Voesenek LACJ, Peeters AJM, Millenaar FF. Hormone- and Light-Mediated Regulation of Heat-Induced Differential Petiole Growth in Arabidopsis. Plant Physiol. 2009;151:1446–58.

86. Crawford AJ, McLachlan DH, Hetherington AM, Franklin KA. High temperature exposure increases plant cooling capacity. Curr Biol. 2012;22:R396–7.

87. Kozuka T, Horiguchi G, Kim GT, Ohgishi M, Sakai T, Tsukaya H. The different growth responses of the Arabidopsis thaliana leaf blade and the petiole during shade avoidance are regulated by photoreceptors and sugar. Plant Cell Physiol. 2005;46:213–23.

88. Coppens F, Wuyts N, Inzé D, Dhondt S. Unlocking the potential of plant phenotyping data through integration and data-driven approaches. Curr Opin Syst Biol. 2017;4:58–63.

89. Araus JL, Kefauver SC, Zaman-Allah M, Olsen MS, Cairns JE. Translating High-Throughput Phenotyping into Genetic Gain. Trends Plant Sci. 2018;23:451–66.

90. Valmadre J, Bertinetto L, Henriques JF, Vedaldi A, Torr PHS. End-to-end representation learning for Correlation Filter based tracking. 2017 IEEE Conf Comput Vis Pattern Recognit. 2017. p. 5000–8.

91. Li L, Zhang Q, Huang D. A review of imaging techniques for plant phenotyping. Sensors. 2014;14:20078–111.

FIGURE LEGENDS

Figure 1. Capturing dynamic plant growth traits using photometric stereo imaging. (**A**) PS comprises a circular arrangement of NIR LEDs with a central camera positioned above the plant(s). Red-dashed lines show the direction of light vectors. (**B, C**) Assembled PS-Plant system shown from side and top views. Each LED is attached to a dedicated heatsink and angled at 30º using a custom 3D printed bracket to minimise the light distribution across the field of view. Both the camera and light sources are stationary.

Figure 2. Evaluating the accuracy of PS-Plant with 2D and 3D data. (**A**, **B**) The estimated area and inclination angle of a flat, matte object (600 mm^2) from 0º to 45º at 5º intervals. Each data point represents the average of thirty randomly selected regional patches of varying size (35-600 mm^2). (**C, D**) The area of three similarly sized Arabidopsis whole rosettes (750 ± 13.5 mm^2) and leaf inclination angles were estimated from 0-45º at 5º intervals. The dashed-black lines indicate ground truth measurements. Error bars represent ±SD of the means.

Figure 3. Data outputs of PS-Plant for Arabidopsis. (**A**) Surface normal map (top) rendered for a wild-type Arabidopsis rosette used to derive models for surface inclination (middle) and convexity (bottom). (**B**) Projected rosette area estimates captured for wild-type plants under standard growth conditions (22 °C, 150 µmol photons m^{-2} s^{-1}, 12 : 12 h light : dark) for 2D and 3D data from the mean ± SE values of 13 biological replicates. (**C**) Percentage difference between 2D and 3D estimations. (**D**) Estimated rosette mean inclination angles across the rosette surface. (**E-H**) Circularity, compactness, diameter and perimeter estimates derived from 2D data.

Figure 4. PS-Plant shows that Arabidopsis plants grown under different conditions show differences in growth architecture. (**A**) Wild-type Arabidopsis plants (24 DAG) following growth under nine different light and temperature conditions. (**B**) Estimated 3D projected rosette area growth of rosettes grown under the different environments. (**C-E**) Estimated 3D projected area, fresh weights and leaf count for rosettes at 24 DAG. (**F-H**) The average relative expansion rate (RER) during light and dark periods for each growth condition (calculated from 15-18 DAG with a 4 hr sliding window). Values represent the mean ± SE values of at least three biological replicates. Asterisks indicate significant differences between light and dark

values for each condition based on Student's t-test ($p < 0.05$). The colour legends in **A** are applicable to **B**, and **F-H**.

Figure 5. Arabidopsis plants grown under different conditions show differences in circadian movement. (**A-C**) The relative rosette surface inclination (i.e. rosette surface inclination following baseline detrending and alignment to the mean) for plants grown in high, medium and low light from 15-18 DAG (see Supplementary Figure S3 for full data sets). (**D**) Period, phase and amplitude calculated by the MFourFit method [77] using data from 11-24 DAG. Values are the mean ±SD of measurements made on at least three biological replicates. Values within each column followed by different letters are significantly different from each other and values followed by the same letter are not ($P < 0.05$) as determined by ANOVA followed by Tukey's HSD tests.

Figure 6. Automated segmentation of individual Arabidopsis leaves using PS-Plant data. Examples are shown based on the Mask R-CNN architecture for plants grown in ML at three different temperatures. (**A**) Composite input images are composed of surface normals in x, y directions and albedo data. (**B**) Manually labelled images (ground truth) used for training. (**C**) Mask R-CNN outputs images showing automated leaf segmentation. For ground truth images and Mask-RCNN outputs each leaf was assigned a unique arbitrary colour.

Figure 7. Automated tracking of leaf labels from segmented Arabidopsis rosettes. (**A**) Three consecutive frames for labelled leaves produced using the trained Mask R-CNN architecture (as in Fig. 6). (**B**) Tracked leaves retained the same colour after applying label tracking (see Rich Media 4). The particle filter allowed calibration of a variety of parameters, including span (the velocity of 'span + 1' recent frames), search radius (the furthest distance

(in pixels) an object may travel between frames), frame memory (the maximum number of frames a seen/tracked object that is absent will be remembered) and filter (the minimum number of frames an object must be seen/tracked to be included). The following particle filter settings produced the best results: span (10), search radius (30), frame memory (3) and filter (100). (**C**) Example of leaf tracking using leaf centroid locations. Each coloured line represents the movement of the centroid location of an individual leaf from 11 to 24 DAG.

Figure 8. Analyses of growth and movement for individual leaves. (**A**) Key landmarks for leaf analysis: rosette origin (P_o), leaf base/leaf blade and petiole intersection point (P_B) and leaf tip (P_T). Data are shown from plants grown in ML at three different temperatures [17 °C (LT) 22 °C (MT) or 27 °C (HT)]. (**B, C**) Leaf blade area and mean surface inclination of a maturing leaf (leaf 1) and an immature leaf (leaf 4) from 15 to 18 DAG. Error bars represent the mean ± SE of three separate leaves. (**D**) Period, phase and amplitude values of the leaf blade from immature leaves (leaves 3 and 4; n = 6 leaves). Letters above the error bars indicate significant differences within each data type ($p < 0.05$) as determined by ANOVA followed by Tukey's HSD tests. Data sets with the same letter are not significantly different. (**E**) The ratio of leaf blade: petiole length for leaves 1 to 4 (L1 to L4). Values represent the mean ratio over 24 hr (17-18 DAG) for three separate leaves. Letters indicate significant differences ($p < 0.05$) within each leaf data set for different temperatures (i.e. L1, L2, L3 and L4).

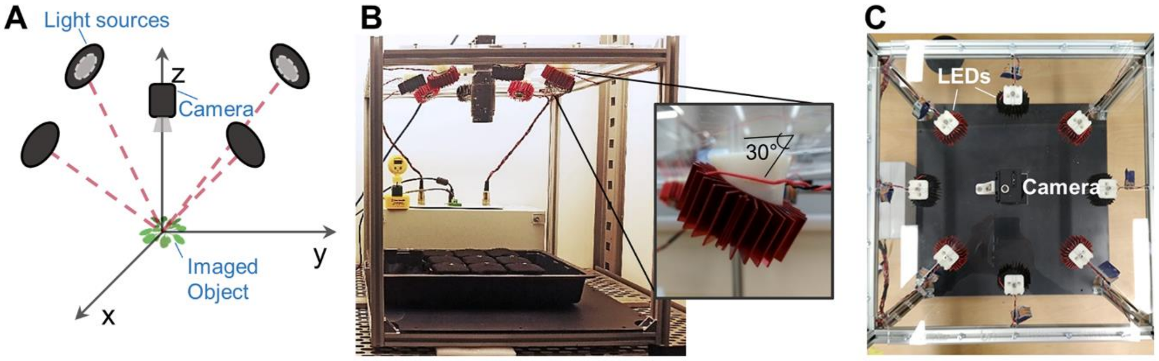

Figure 1.

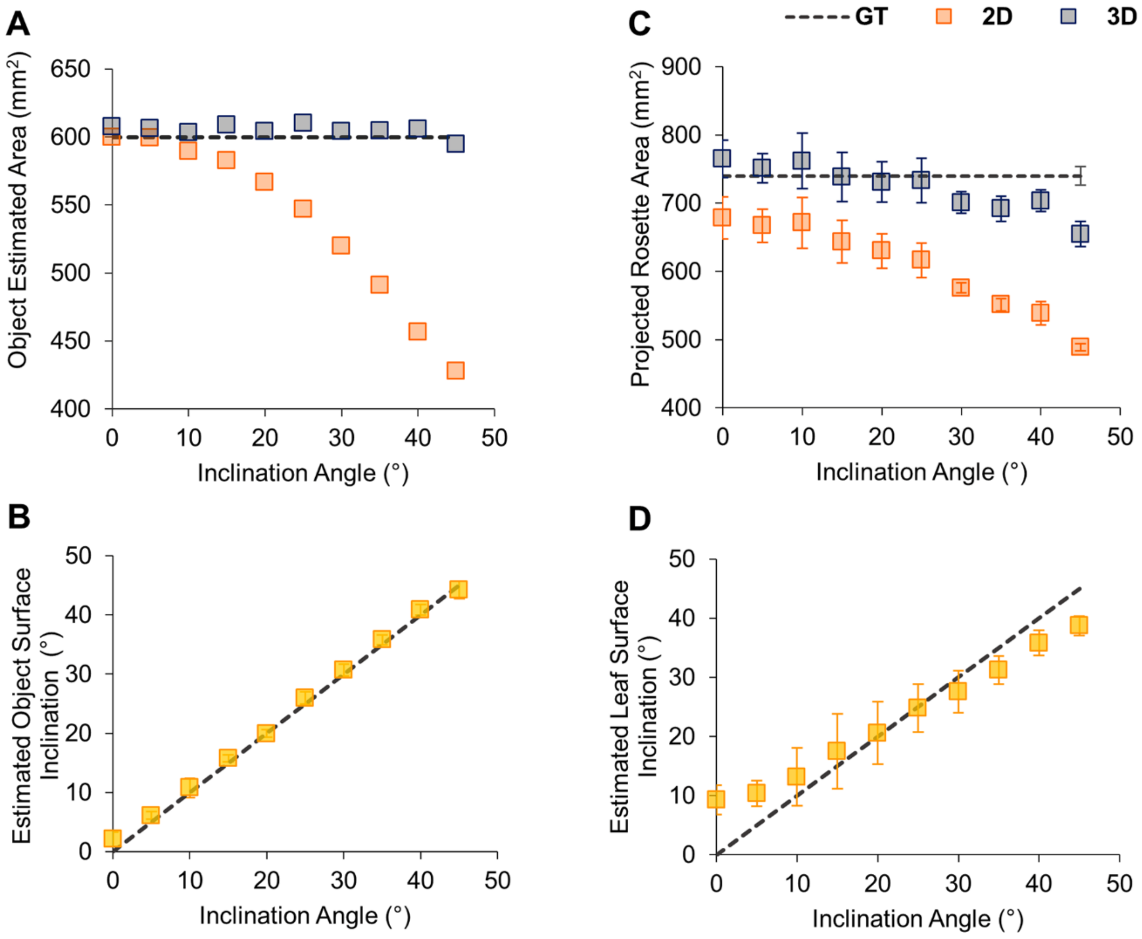
A
Object Estimated Area (mm²)
650
600
550
500
450
400
0
10
20
30
40
50
Inclination Angle (°)
B
Estimated Object Surface Inclination (°)
C
GT
2D
3D
Projected Rosette Area (mm²)
900
800
700
600
500
400
D
Estimated Leaf Surface Inclination (°)

Figure 2.

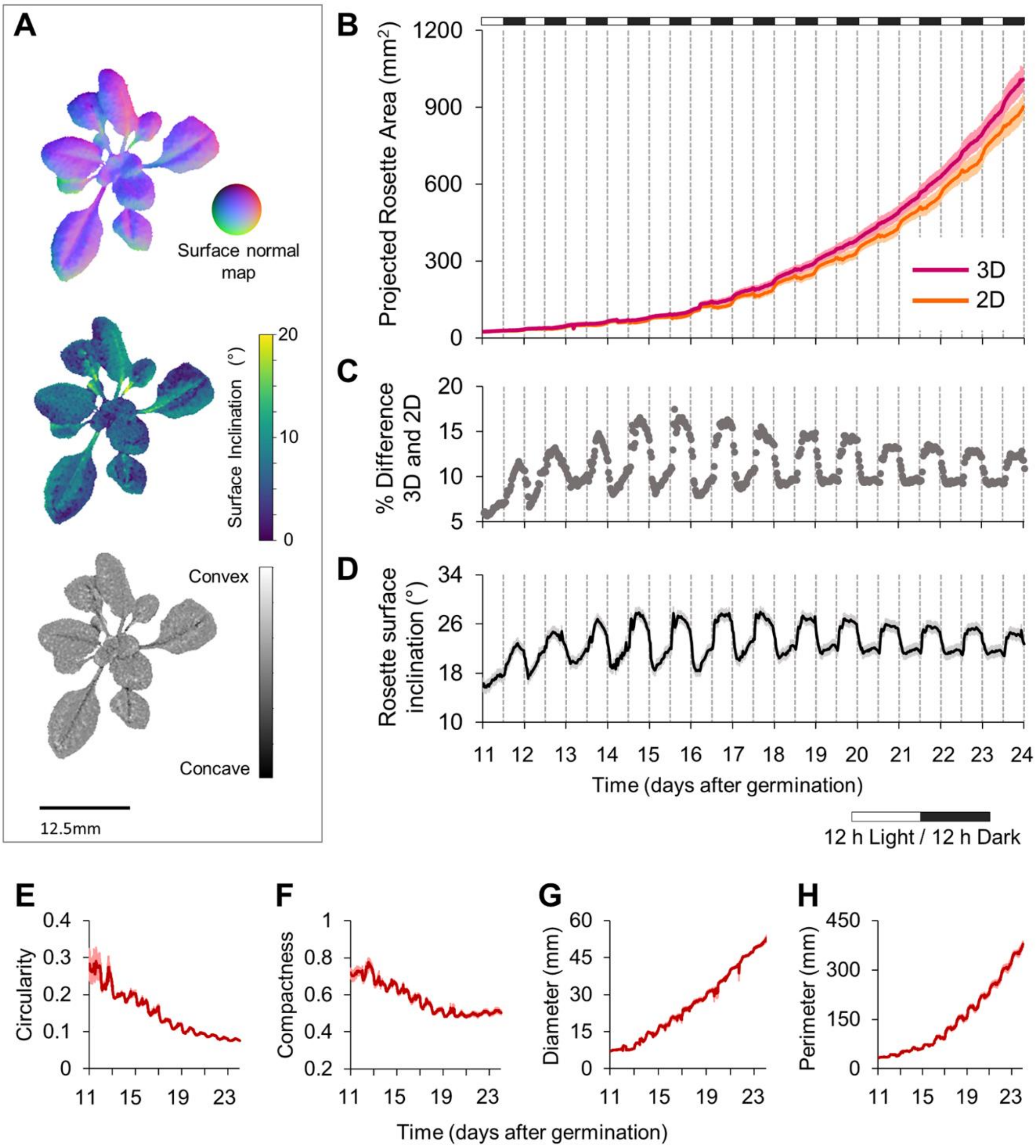
A
Surface normal map
Surface Inclination (°)
20
10
0
Convex
Concave
12.5mm
B
Projected Rosette Area (mm²)
1200
900
600
300
0
3D
2D
C
% Difference 3D and 2D
20
15
10
5
D
Rosette surface inclination (°)
34
26
18
10
11 12 13 14 15 16 17 18 19 20 21 22 23 24
Time (days after germination)
12 h Light / 12 h Dark
E
Circularity
0.4
0.3
0.2
0.1
0
F
Compactness
1
0.8
0.6
0.4
0.2
G
Diameter (mm)
60
45
30
15
0
H
Perimeter (mm)
450
300
150
0
11 15 19 23
Time (days after germination)

Figure 3.

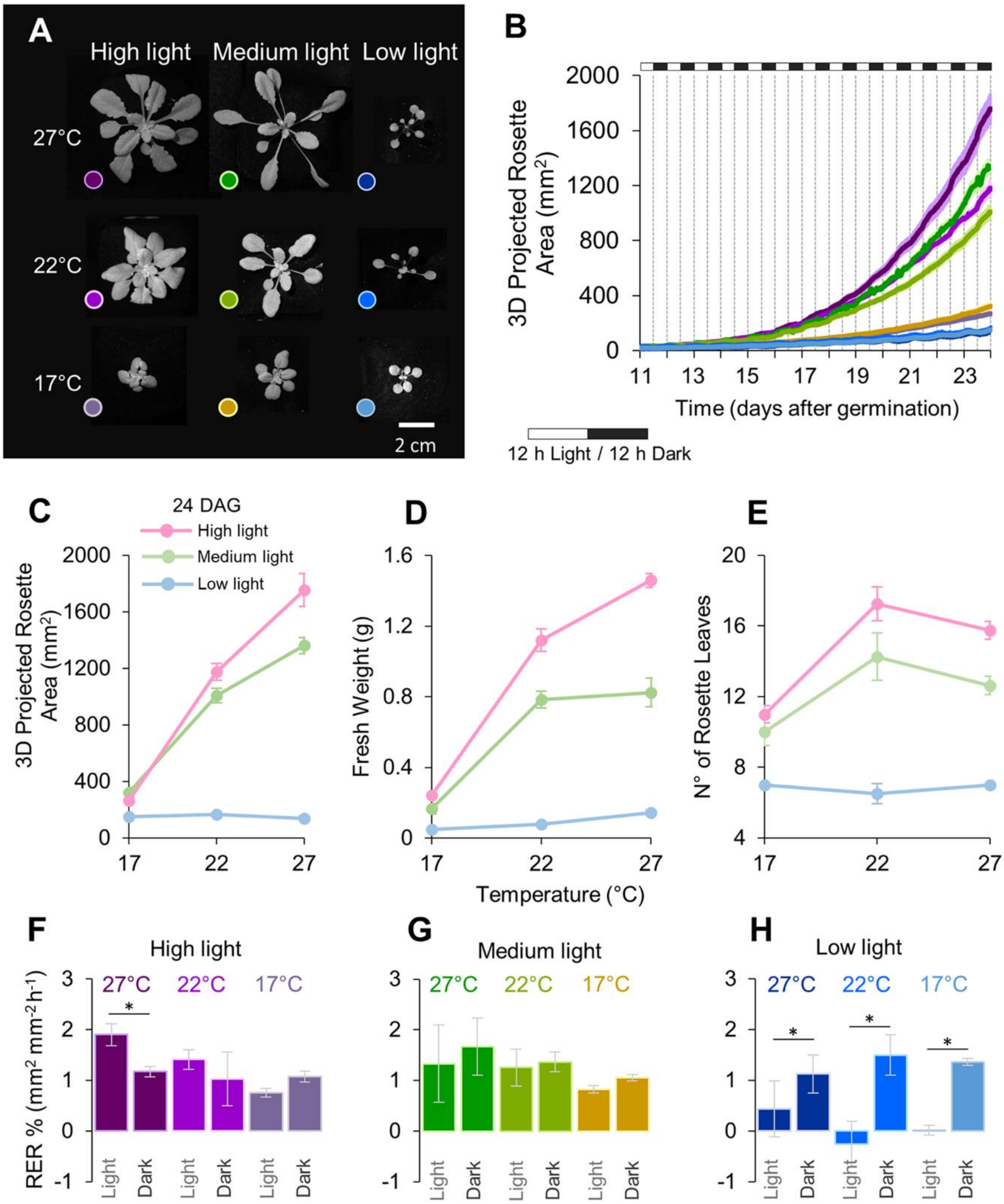
A
High light
Medium light
Low light
27°C
22°C
17°C
2 cm
B
3D Projected Rosette Area (mm2)
2000
1600
1200
800
400
0
11
13
15
17
19
21
23
Time (days after germination)
12 h Light / 12 h Dark
C
24 DAG
High light
Medium light
Low light
3D Projected Rosette Area (mm2)
D
Fresh Weight (g)
E
N° of Rosette Leaves
Temperature (°C)
F
High light
RER % (mm2 mm-2 h-1)
27°C
22°C
17°C
Light
Dark
G
Medium light
H
Low light

Figure 4.

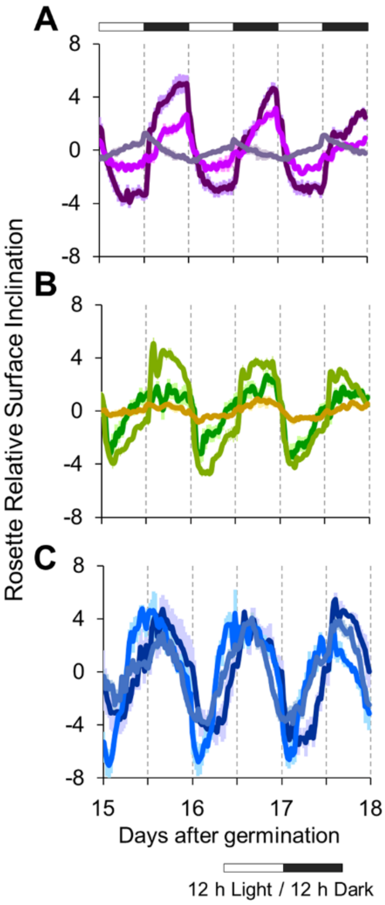

D

Treatment		Period (h)	Phase (h)	Amplitude (°)
High Light	27 °C	24.1 ± 0.2 ^{a}	21.9 ± 1.0 ^{ab}	2.7 ± 0.2 ^{b}
	22 °C	24.0 ± 0.1 ^{a}	23.1 ± 0.8 ^{a}	1.6 ± 0.5 ^{c}
	17 °C	24.5 ± 0.4 ^{a}	10.5 ± 2.4 ^{f}	1.0 ± 0.4 ^{cd}
Medium Light	27 °C	24.1 ± 0.1 ^{a}	17.9 ± 2.0 ^{cd}	2.6 ± 0.3 ^{b}
	22 °C	24.1 ± 0.1 ^{a}	18.9 ± 1.2 ^{bc}	3.2 ± 0.6 ^{ab}
	17 °C	24.3 ± 0.4 ^{a}	15.1 ± 3.6 ^{de}	0.5 ± 0.1 ^{d}
Low Light	27 °C	24.1 ± 0.1 ^{a}	15.4 ± 1.3 ^{cde}	3.4 ± 0.7 ^{ab}
	22 °C	24.3 ± 0.2 ^{a}	11.9 ± 2.2 ^{ef}	3.7 ± 0.3 ^{a}
	17 °C	24.1 ± 0.1 ^{a}	15.0 ± 0.8 ^{de}	2.8 ± 0.6 ^{ab}

Figure 5.

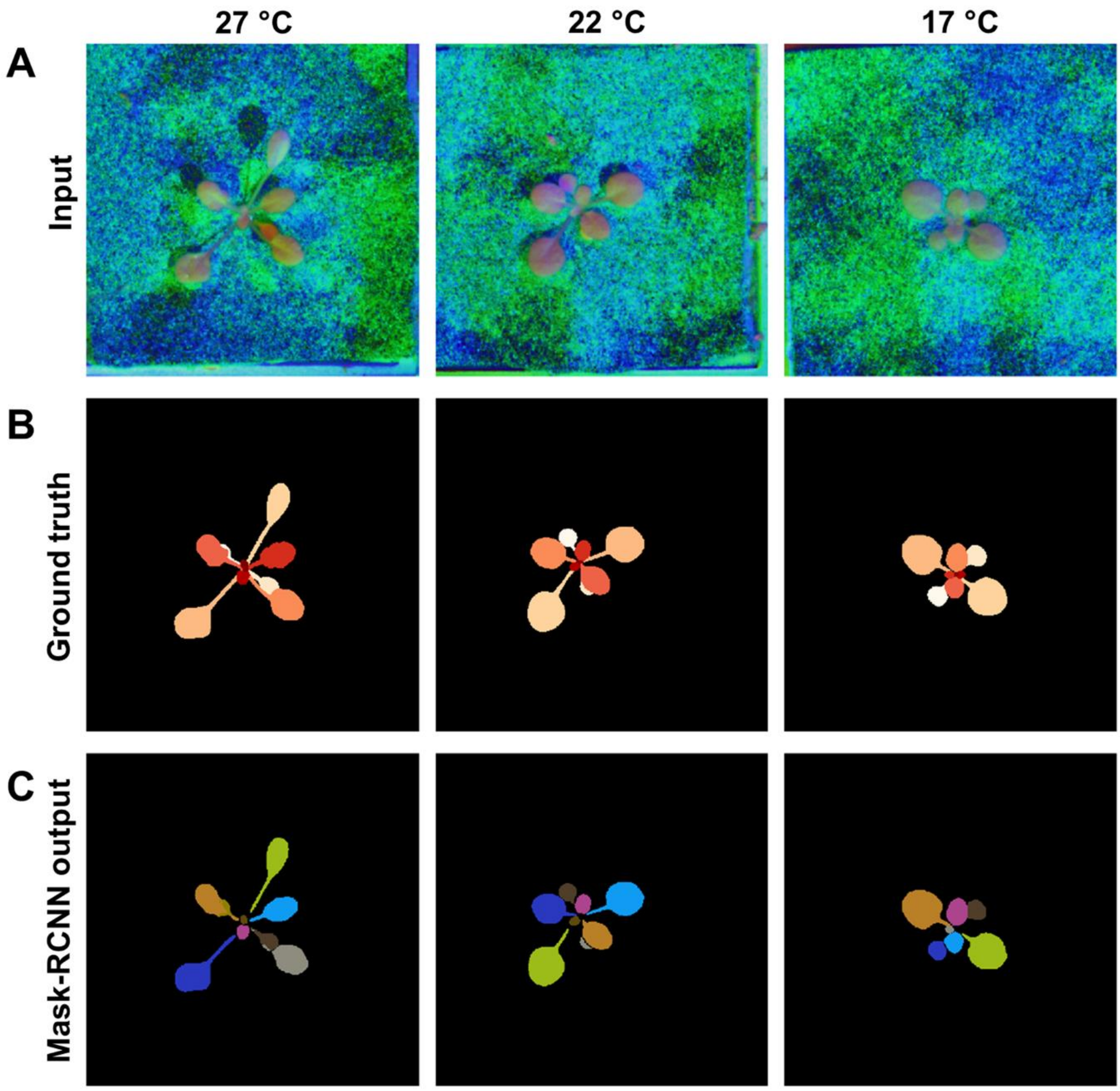

Figure 6.

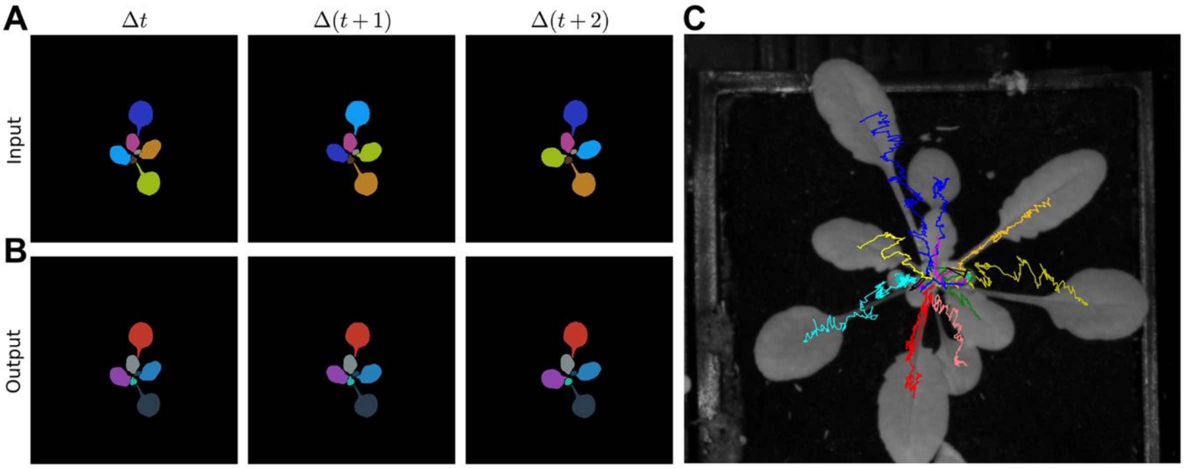

Figure 7.

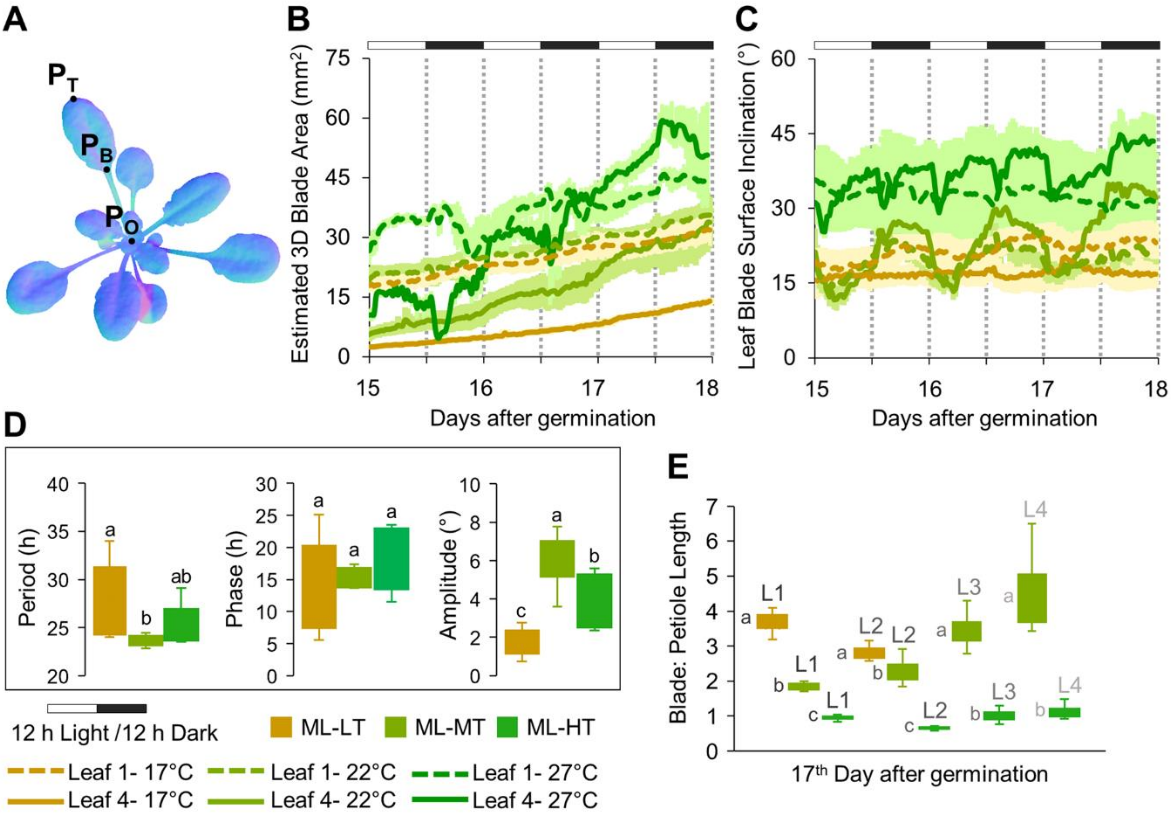
A
P_T
P_B
P_O
B
Estimated 3D Blade Area (mm²)
75
60
45
30
15
0
15
16
17
18
Days after germination
C
Leaf Blade Surface Inclination (°)
60
45
30
15
0
15
16
17
18
Days after germination
D
Period (h)
40
35
30
25
20
a
b
ab
Phase (h)
30
25
20
15
10
5
0
a
a
a
Amplitude (°)
10
8
6
4
2
0
c
a
b
12 h Light /12 h Dark
ML-LT
ML-MT
ML-HT
Leaf 1- 17°C
Leaf 1- 22°C
Leaf 1- 27°C
Leaf 4- 17°C
Leaf 4- 22°C
Leaf 4- 27°C
E
Blade: Petiole Length
7
6
5
4
3
2
1
0
L1
L1
L1
L2
L2
L2
L3
L3
L4
L4
a
b
c
a
b
c
a
b
a
b
17th Day after germination

Figure 8.

Supplementary Data S1

Click here to access/download
Supplementary Material
Supplementary Data S1.stl

Supplementary Data S2

Click here to access/download
Supplementary Material
Supplementary Data S2.mp4

Supplementary Data S3

Click here to access/download
Supplementary Material
Supplementary Data S3.mp4

Click here to access/download
Supplementary Material
Supplementary Data S4.mp4

Supplementary Data S5A

Click here to access/download
Supplementary Material
Supplementary Data S5A - Leaf 1.mp4

Click here to access/download
Supplementary Material
Supplementary Data S5B - Leaf 2.mp4

Click here to access/download
Supplementary Material
Supplementary Data S5C - Leaf 3.mp4

Click here to access/download
Supplementary Material
Supplementary Data S5D - Leaf 4.mp4

Click here to access/download
Supplementary Material
Supplementary Information S1-S5, Figures S1-S4.pdf
