# Supplementary material for: A photometric stereo-based 3D imaging system using computer vision and deep learning for tracking plant growth
Source: Gigascience. 2019 May 25;8(5):giz056. doi: 10.1093/gigascience/giz056 (PMC6534809; doi:10.1093/gigascience/giz056)
Supplement: giz056_Supplemental_Files [file giz056_supplemental_files.zip › Supplementary Info S1-S5, Fig S1-S4 190415.docx]

**Supplementary Information S1.** Overview of 2D image data processing captured using PS-Plant.

*Photometric stereo*

The photometric stereo (PS) principle is a technique that captures a matrix of images by imaging an object under controlled, varied illumination from at least three differing lighting conditions (Fig. 1) [1]. The obtained images are then used to determine both the surface normal and albedo at each pixel in the image:

$I=\varrho\cdot N\cdot L$ (1)

$\varrho=\left| L^{-1}\cdot I \right|$ (2)

N = $\frac{\varrho\cdot N}{\varrho}$ (3)

where I is the image matrix, $\varrho$ is albedo, N is the surface normal map and L is a matrix of the direction vectors of the light-sources. The obtained dense surface normal map is a map of the same resolution as the image captured of the scene, where each pixel has a vector that is perpendicular to that pixel when projected onto the object surface, called a normal (Fig. 1A). The normal for each pixel in the image allows the overall orientation of the object to be determined. Due to the abundance of information, it is common practice to present this data in a colour coded figure (e.g. Fig. 1B) where the x, y and z components are represented in the red (R), green (G) and blue (B) channels, respectively. The acquired albedo indicates how well the surface reflects light and provides data of the imaging surface (it is not affected by the illumination direction or intensity). Pixels in an albedo image hold a value in the range from 0 (black) to 1 (white) (Fig. 1C).

| 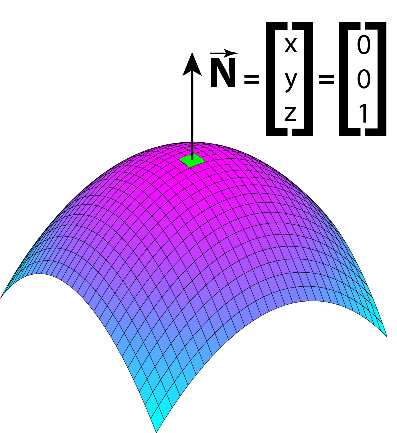  **A B C** | **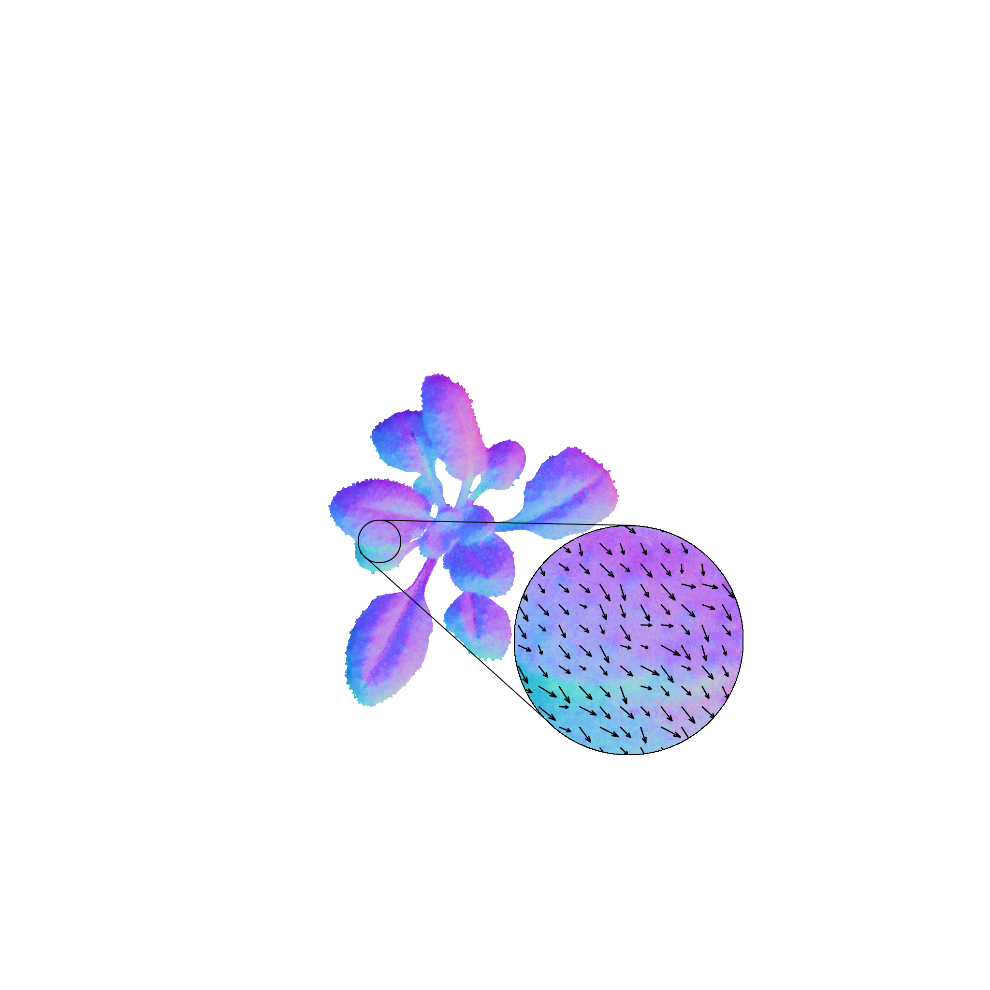** | **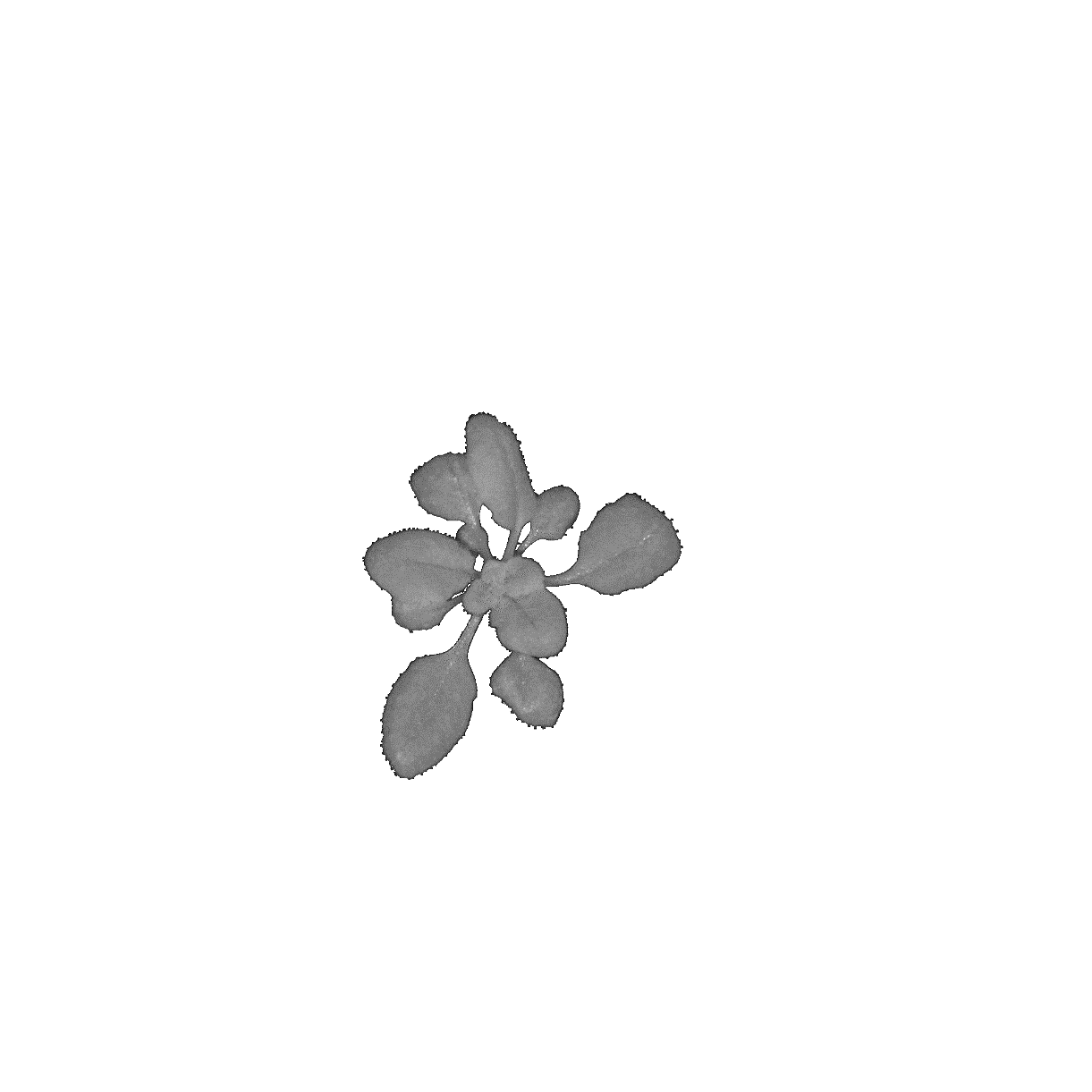** |
| --- | --- | --- |

**Figure 1.** **Surface normal information capture by the Photometric Stereo approach.** (A) Normal ($\vec{N}$) representation on a surface. (B) Colour coded normal map of a wild-type Arabidopsis rosette and a zoomed-in normal map of a leaf surface (the normal is shown on every tenth pixel in both x and y directions) where arrow lengths indicate the magnitude along the z-axis. (C) Albedo image of an Arabidopsis rosette.

*Light field compensation and adaptive light source vectors to improve the accuracy of photometric stereo*

PS assumes that illumination is a point source. A point source is a mathematical model of a light source whereby the distance from the light to the object tends to infinity, providing an even collimated illumination across the imaging area. However, point sources are not feasible in practice as the light intensity of light sources changes with distance from the object according to the inverse-square law:

$intensity\propto\frac{1}{distance^{2}}$ (4)

Without correction this can lead to inaccurate 3D data with PS, as shown for a flat sheet of paper in Fig. 2A-C. As pixels captured within an image differ in distance from the light sources, larger errors can occur for normal estimations and 3D reconstructions the further the pixel is from the centre of the image. Therefore, to obtain better reconstruction results we bootstrapped PS calculations (Eq. 1-4) with a light field compensation model based on the inverse-square law (Fig. 2D-F) [2]. Traditional PS also assumes that the given light source vectors are applicable to every pixel in the image. However, this hold true only for pixels located in the centre of the surrounding light sources, while the accuracy of positional vectors deviates with an increasing distance from the centre. Accurate PS requires knowledge of the light source vector deviations of every pixel to solve equations (2) and (3). Thus, unique light source vectors for each pixel in the image were obtained to improve the reconstruction accuracy (Fig. 2G-I) [3].

| 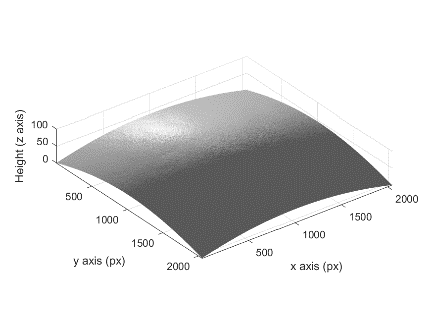  **A B C**  **D E F**  **G H I** | 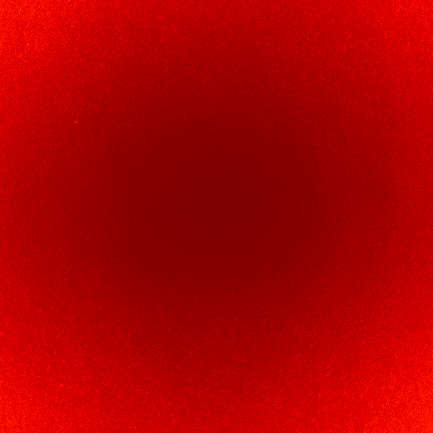 | 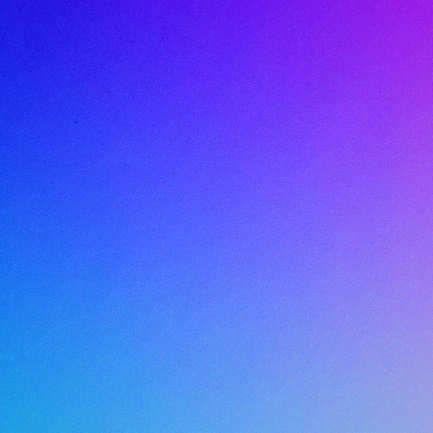 |
| --- | --- | --- |
| 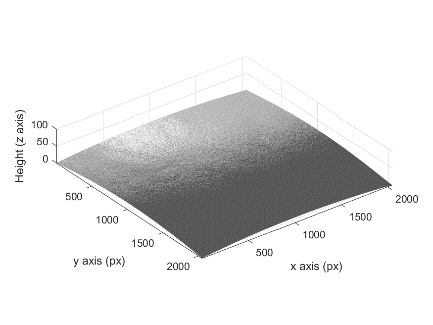 | 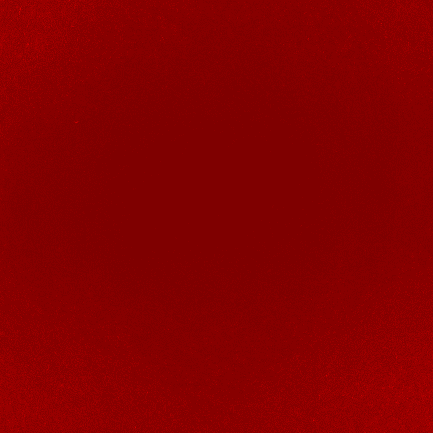 | 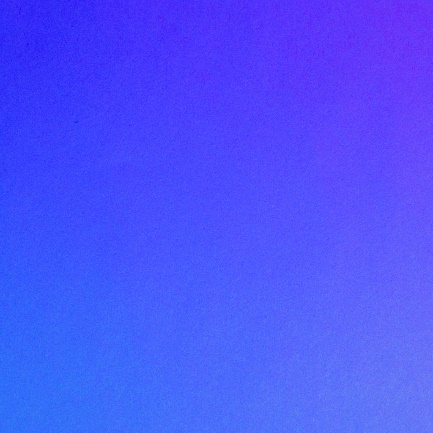 |
| 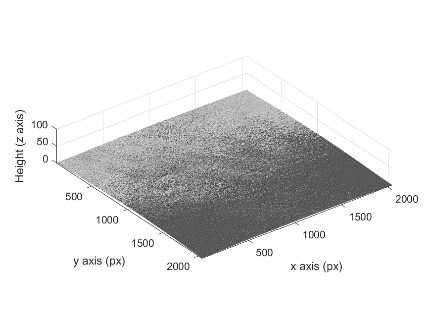 | 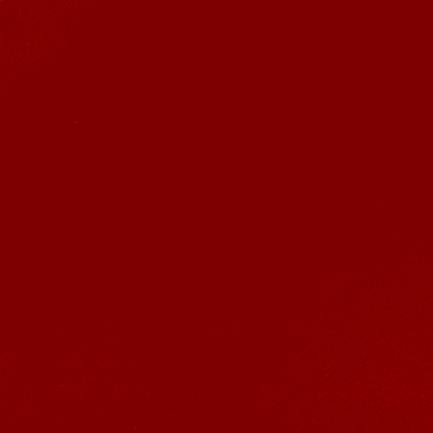 | 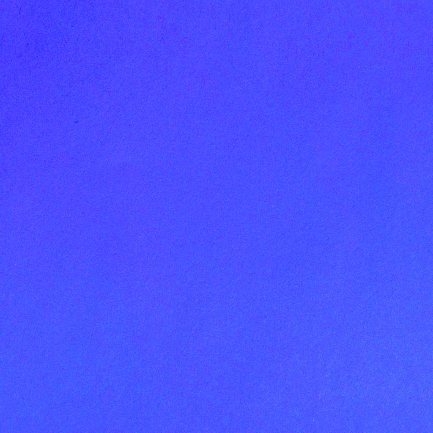 |

**Figure 2.** **Comparison of light field compensation and adaptive light source vectors to photometric stereo images of a flat sheet of paper**. Integrated 3D shape by utilising traditional PS formulation (A), with pixel orientations along the z-axis (B) and surface normal map (C). (D-F) Integrated 3D shape after applying the inverse-square law compensation algorithm. (G-I) Integrated 3D shape after applying the inverse-square law compensation and per-pixel light source vector estimation algorithms.

We then quantitatively compared the accuracy of traditional PS with inverse square law compensation or inverse square law compensation with additional per-pixel light source vectors (Fig. 3) by calculating the angle deviation from the ground truth and the estimated normal vectors as dot products:

$\theta= \cos^{-1} \frac{u\cdot v}{\left\| u \right\|\left\| v \right\|}$ (5)

where $u$ and $v$ are two vectors and $\theta$ is angle between the vectors. It was assumed that the imaging surface was flat, thus, the ground truth values for every pixel in the image is normal to the camera (pointing along the z axis) (Fig. 1A).

The obtained angle deviation for every pixel in images from each of the three approaches was then used to find the mean angle deviation. Traditional PS had the largest deviation of 0.346 radian (approx. $19.82^{\circ}$) (Fig. 3A). Applying the inverse square law compensation reduced the deviation to 0.239 radian (approx. 13.69$^{\circ}$) (Fig. 3B), while the best results were obtained using a combination of inverse square law compensation and per-pixel light source vector estimation algorithms - 0.066 radian (approx. 3.79$^{\circ}$) (Fig. 3C). Overall, the latter resulted in an 80% reduction in error when compared to traditional PS results.

| 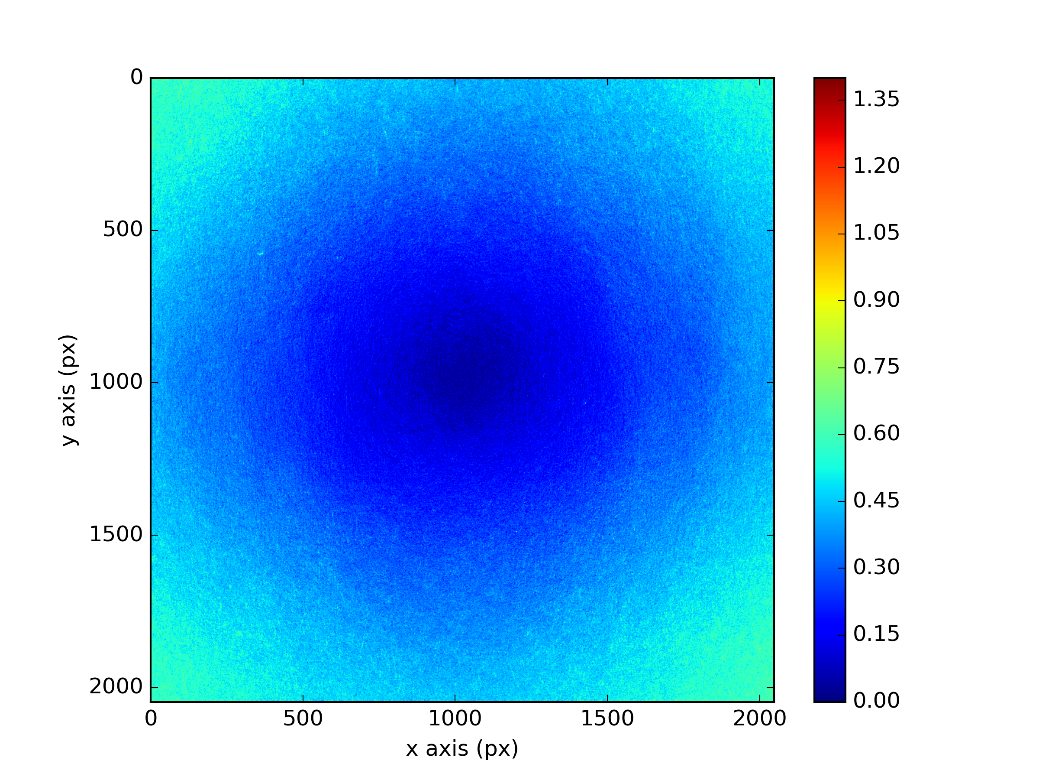  **A** |
| --- |
| 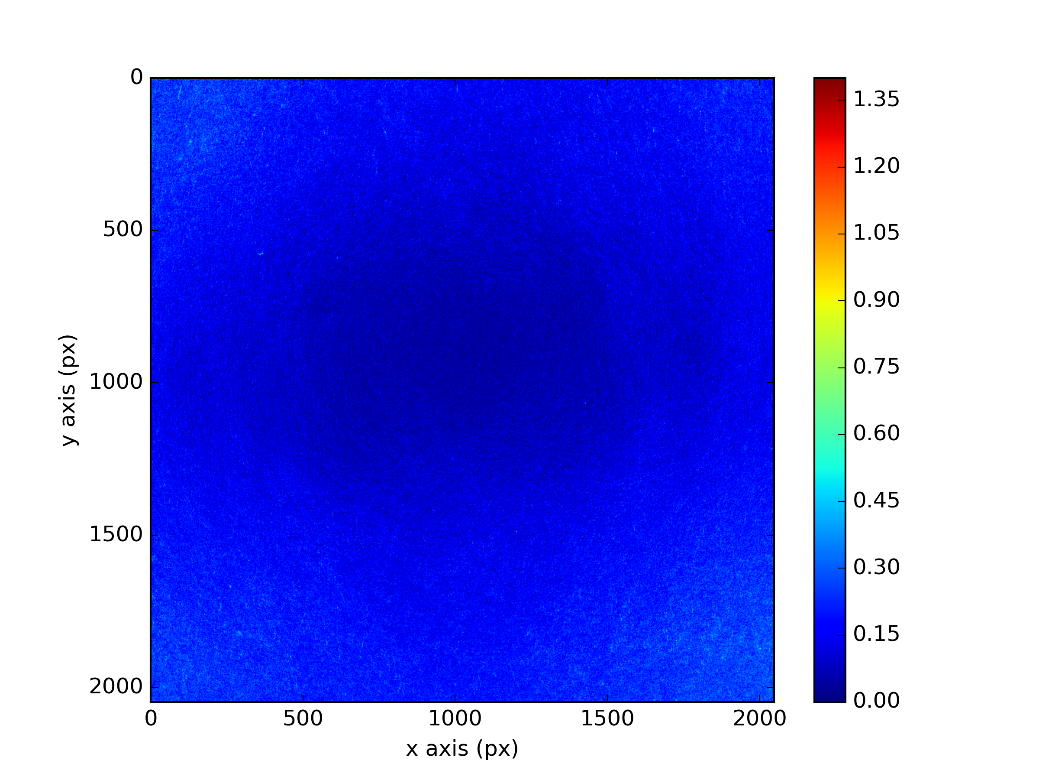  **B**  **C** |
| 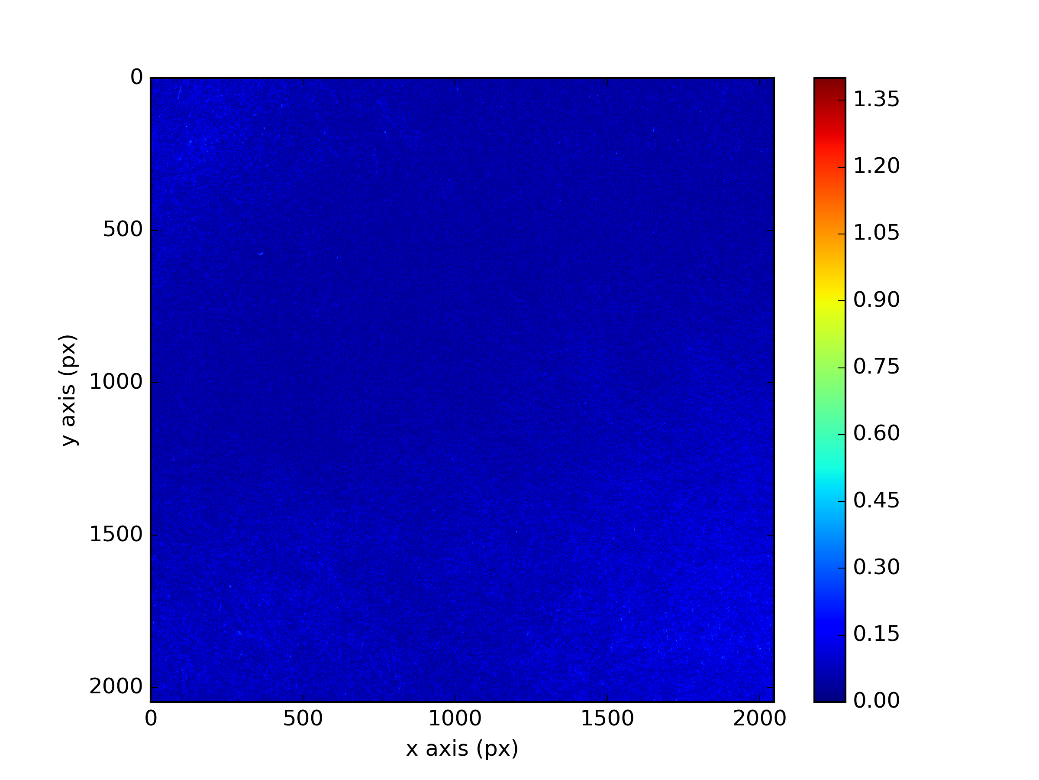 |

**Figure 3.** **Angle deviation from ground truth for photometric stereo images of a flat sheet of paper.** (A) Traditional photometric stereo in the absence of light field compensation and per-pixel light source vectors. (B) Application of the inverse-square law compensation algorithm. (C) Application of the inverse-square law compensation and per-pixel light source vector estimation algorithms. The colour bar on the right shows the deviation error in radians.

*3D surface integration*

Following light field compensation and per-pixel light source vector estimation, a more accurate surface normal map was obtained and used to reconstruct the underlying 3D shape of the object. The normal map has three components, $N_{x}, N_{y}$ and $N_{z}$, indicating which direction each pixel is pointing (Fig. 4). Using surface normal orientations at a given pixel, the surface gradient components can be calculated [4]:

$p=-\frac{N_{x}}{N_{z}}$ ; $q=-\frac{N_{y}}{N_{z}}$ (7)

The obtained gradients ($p, q$) are components of a surface gradient vector and are perpendicular to the normal of a surface/pixel and tangent to the surface. The gradients were supplied to the Frankot-Chellappa integration algorithm to reconstruct the underlying 3D shape of the object [5], which was used as a reference to confirm the accuracy of the acquired data.

| 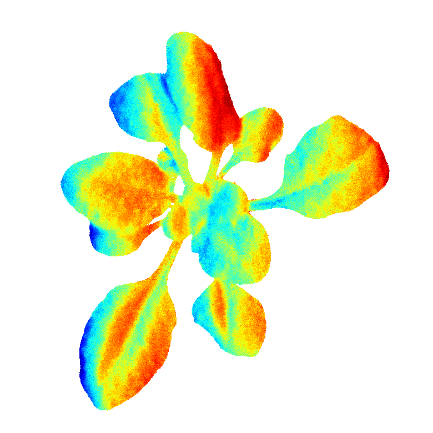  **x y z** | 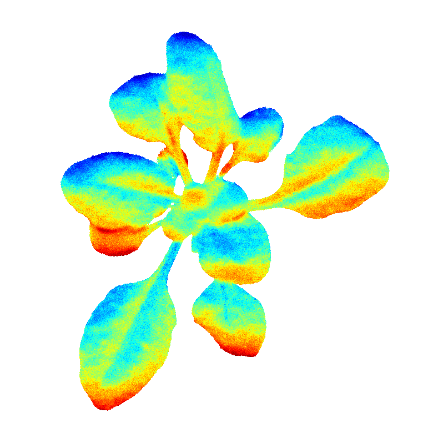 | 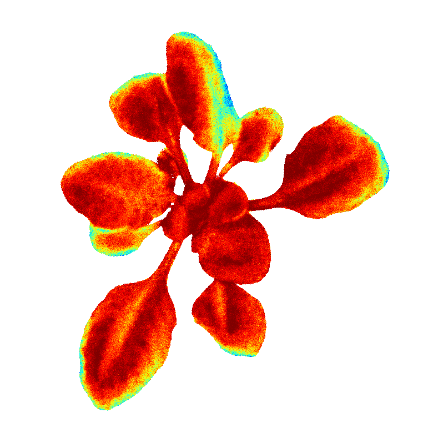 |
| --- | --- | --- |

**Figure 4.** **Normal orientations in x, y and z directions for an Arabidopsis rosette image.**

*Surface curvature*

The Gaussian and the mean curvature are the common methods to estimate the surface curvature at a point on a surface, but are not indicative of local surface shape [6]. The maximum ($k_{1}$) and the minimum ($k_{2}$) of the surface curvature at a given point are called principal curvatures, which define the bending of a regular surface in a 3D Euclidean space [8]. As the Gaussian ($K$) and the mean ($H$) curvatures can be expressed in terms of principal curvatures:

$K=k_{1}k_{2}$; $H=\frac{1}{2}(k_{1}+k_{2})$ (8)

the maximum ($k_{1}$) and the minimum ($k_{2}$) principal curvatures can be found:

$k_{1}=H+\sqrt{H^{2}-K}$; $k_{2}= H-\sqrt{H^{2}-K}$ (9)

The obtained principal curvatures can be used to obtain the surface shape index and curvedness that define the measurement of local shape [6]. The shape index ($s$) is a single valued measure of local curvature within a range of [-1, 1], while curvedness ($c$) is the magnitude of a local curvature in the region within a range of [0, 1]. Both ($s$) and ($c$) can be calculated [6]:

$s= \frac{2}{\pi}{tan}^{-1}\frac{k_{2}+k_{1}}{k_{2}-k_{1}}$; $c=\sqrt{\frac{k_{1}^{2}+k_{2}^{2}}{2}}$ (10)

As described in the previous sub-section ‘*3D surface integration’*, the surface normal map obtained by PS can be expressed as surface gradients $p$ and $q$. Therefore, the obtained surface gradients can be used to derive the principal curvatures $k_{1}$ and $k_{2}$ as described in [9]. The computed shape index and curvedness of an Arabidopsis rosette are shown in Fig. 5.

**A B**


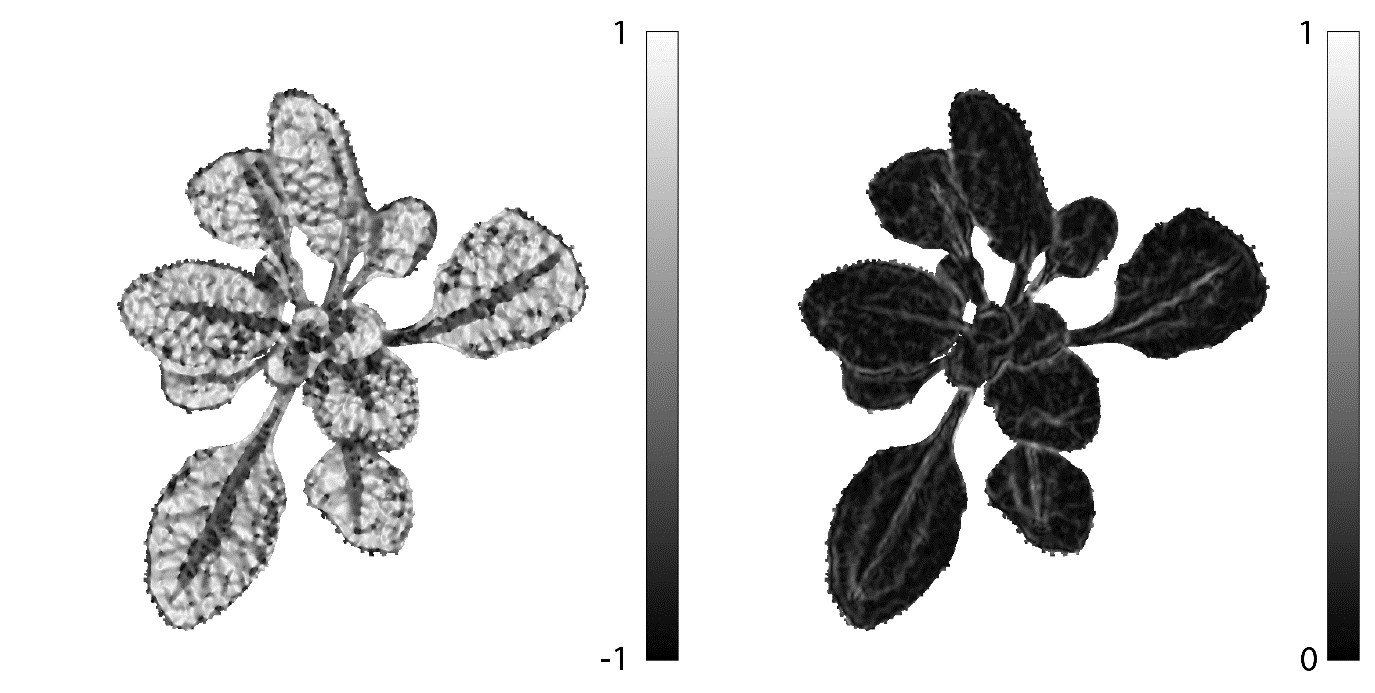


**Figure 5. Surface curvature measures for Arabidopsis rosette.** (A) Surface shape index that defines local curvature [-1, 1] and (B) surface curvedness that defines the local curvature in the region [0, 1]. A Gaussian blur (kernel size of 15 with standard deviation along X and Y-axis of 0) was applied to the PS-obtained surface normal map before the shape index and curvedness were computed.

*Lambert’s cosine law*

An assumption of the original PS method is that the imaging surface must exhibit Lambertian properties - ideally diffuse to reflect the light proportionally in all directions. According to the Lambert’s cosine law, the reflected light intensity depends on the incident light intensity and the cosine angle between the incident light and viewing direction:

$I_{\theta}=I* \cos\theta$ (8)

To our best knowledge, there is no empirical evidence that leaves of *Arabidopsis thaliana* reflect light according to the Lambert’s cosine law. Consequently, we investigated this in a simple experiment with a mature Arabidopsis leaf (harvested 20 days after germination) slightly cut on the sides to reduce the leaf curvature tension and secured on a matt black sheet of paper using masking tape. A camera was positioned 40 cm above the leaf. A NIR illumination source (940 nm) was placed 45 cm away at different incident angles ranging from 10 to 75º at 5-degree intervals, where 90º was above the camera and 0º was parallel to the imaging surface (Fig. 6A,B). Three patches on the leaf surface of random location and size (540 to 825 px) were investigated. The leaf was imaged at different illumination incident angles and the mean pixel intensity value was measured for each patch to obtain z-score values. A z-score is a standard score that indicates how many standard deviations an element is from the mean:

$z= \frac{x-\mu}{\sigma}$ (9)

where $z$ is a standard score, $x$ is element value, $\mu$ is the mean of the investigating values and $\sigma$ is the standard deviation. Z-scores allowed us to plot and evaluate the curve of different experiments despite the differences in light intensity (Fig. 6C). The same experiment was performed on a surface that approximates the Lambertian properties (a diffuse tile purchased from Spectralon Diffuse Reflectance Targets, Labsphere Inc., New Hampshire, United States) as a control to show that the experimental set up could accurately measure the reflectance of an object (Fig. 6D). The obtained results were compared to Lambertian reflectance values derived from (8) by substituting known angle ($\theta$) information to determine the value of the fitted regression line. The average *R*^2^ values for the three patches compared to (8) were 0.998 ±0.001, while the ideal surface was 0.999. Thus, we concluded that Arabidopsis leaf reflectance under 940 nm illumination approximated very closely to Lambert’s cosine law.

| 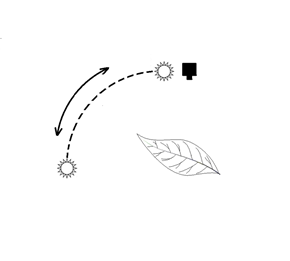 | 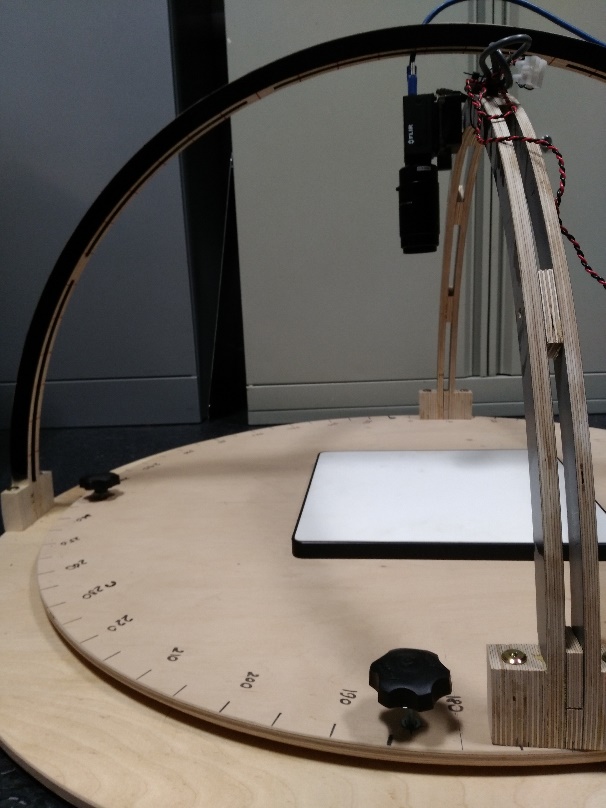  **A B**  **C D** |
| --- | --- |
| 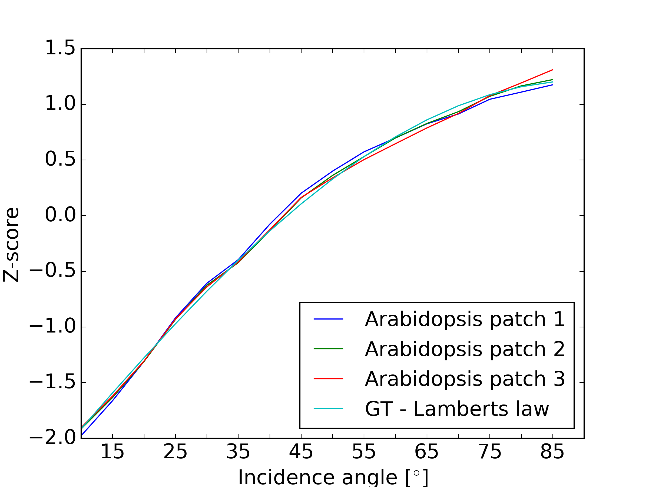  *R*^2^ values:  Patch 1 = 0.997  Patch 2 = 0.999  Patch 3 = 0.998 | 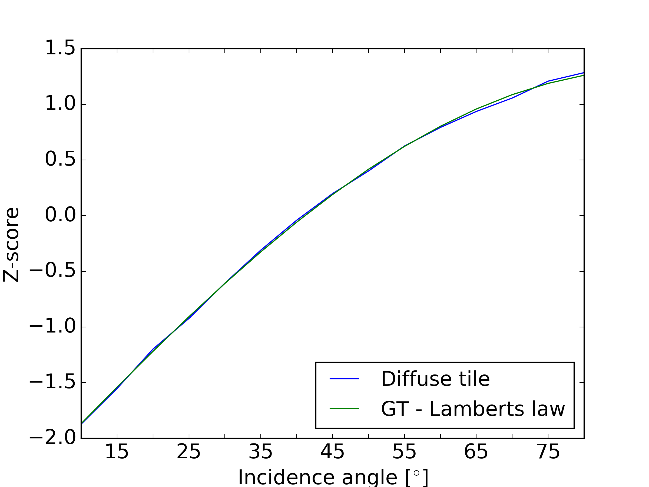  *R*^2^ value:  Diffuse tile = 0.999 |

**Figure 6. Arabidopsis leaves follow**

**hile pefecte perfect results e as follows tical rface ensity. inclinatin
 hile pefecte perfect results e as follows tical rface ensity. inclinatin Lambert’s cosine law.** (A) Conceptual schematic of the movement of the NIR light source during the experiment. (B) The rig used to image the object (the diffuse tile is shown), secure the camera above the object and slide the illumination source to change the incidence angle (as in (A)). (C) Reflectance results of three random patches of an Arabidopsis leaf compared to the ground truth obtained from Eq. 8. Coefficient of determination (*R*^2^) values are shown for the three patches. (D) Reflectance results of the diffuse tile.

**References**

1. Woodham RJ. Photometric Method For Determining Surface Orientation From Multiple Images. Opt. Eng. 1980;19:139–144.

2. Iwahori Y, Sugie H, Ishii N. Reconstructing shape from shading images under point light source illumination. Proc. Int. Conf. Pattern Recognit. 1990;83–87.

3. Ahmad J, Sun J, Smith L, Smith M. Improving photometric stereo through per-pixel light vector calculation. Br. Mach. Vis. Conf. 2013;1–12.

4. Argyriou V, Petrou M. Photometric Stereo: An Overview. Elsevier Inc.; 2009.

5. Frankot RT, Chellappa R. Method for Enforcing Integrability in Shape From Shading Algorithms. IEEE Trans. Pattern Anal. Mach. Intell. 1988;10:439–451.

6. Koenderink JJ, van Doorn AJ. Surface shape and curvature scales. Image Vis. Comput. 1992;10:557–564.

7. Tanaka HT, Ikeda M, Chiaki H. Curvature-based face surface recognition using spherical correlation. Principal directions for curved object recognition. Proc. - 3rd IEEE Int. Conf. Autom. Face Gesture Recognit. 1998. p. 372–377.

8. Zhang W, Hansen MF, Smith M, Smith L, Grieve B. Photometric stereo for three-dimensional leaf venation extraction. Comput. Ind. Elsevier B.V.; 2018;98:56–67.

9. Woodham RJ. Determining Surface Curvature with Photometric Stereo. Int. Conf. Robot. Autom. 1989. p. 36–42.

10. Ren M, Zemel RS. End-to-End Instance Segmentation with Recurrent Attention. 2016;

11. Scharr H, Minervini M, French AP, Klukas C, Kramer DM, Liu X, et al. Leaf segmentation in plant phenotyping: a collation study. Mach. Vis. Appl. Springer Berlin Heidelberg; 2016;27:585–606.

12. Giuffrida MV, Scharr H, Tsaftaris SA. ARIGAN: Synthetic Arabidopsis Plants using Generative Adversarial Network. Proc. 2017 IEEE Int. Conf. Comput. Vis. Work. 2017. p. 22–29.

**Supplementary Information S2.** Overview of the PS-Plant hardware.

*Rig design*

The PS-Plant rig consisted of 15 aluminium profiles of two different lengths (400 (13) and 190 (2) mm) (20x20 Aluminium Profile, KJN Automation Ltd, United Kingdom) that were secured with angle brackets (20x20 Bracket with Fixings, KJN Automation Ltd, United Kingdom) and two acrylic plates (440 x 440 x 5, Perspex Distribution Ltd, United Kingdom) (Fig. 1; 2). Both camera and LEDs were secured to the top acrylic plate using custom made holders (Fig. 3; 4).


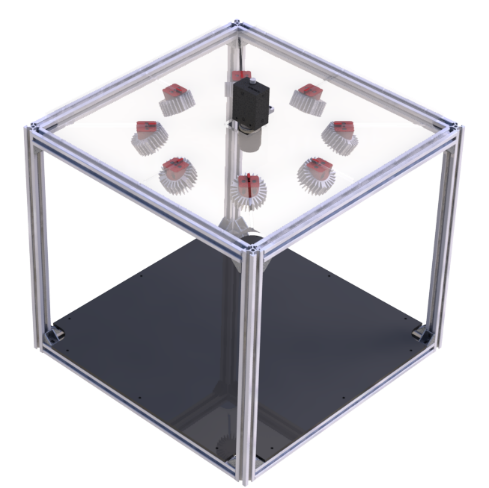


**Figure 1. 3D model of the PS-Plant system.** An online link can be found here: <https://sketchfab.com/models/eaccbca9ffa946d6891719bca640c14e>.

**Figure 2. Schematic design of PS-Plant system.** All measurements are in millimetres (mm).

**Figure 3. Schematic design of the camera holder.** All measurements are in millimetres (mm).

**Figure 4. Schematic design of the LED holder.** All measurements are in millimetres (mm).

*LED controller*

A light controller was specifically designed for this project to allow rapid LED switching rates and consistent illumination at an affordable cost (Fig. 5). The controller size was 100 x 82 x 27 mm (length x width x height) and consisted of 8 LED drivers (LDU2430S1000, XP Power, Singapore), an Arduino microcontroller (MKRZero, Arduino, Italy), three terminal blocks and 16 resistors (eight of each $6.8 kΩ, 18 kΩ$). The Arduino microcontroller allowed an easy interface between a PC and PS-Plant lighting system that controlled the light status. Resistors were chosen for two reasons: 1) to reduce the Arduino voltage from 3.3V to the acceptable input range of LED drivers (0 to 1.25 V) and 2) to provide the LEDs with the correct current value (750 mA). Two terminal blocks with eight outputs were used to connect the printed circuit board (PCB) to the LEDs, while the terminal block with two outputs was used for supplying the power to the LEDs (1.1 A, 24 V DC Power supply, Mean Well, Taiwan). Both the schematics and the PCB design were developed using Eagle software (Autodesk, California, United States) and are provided below (Fig. 6; 7).


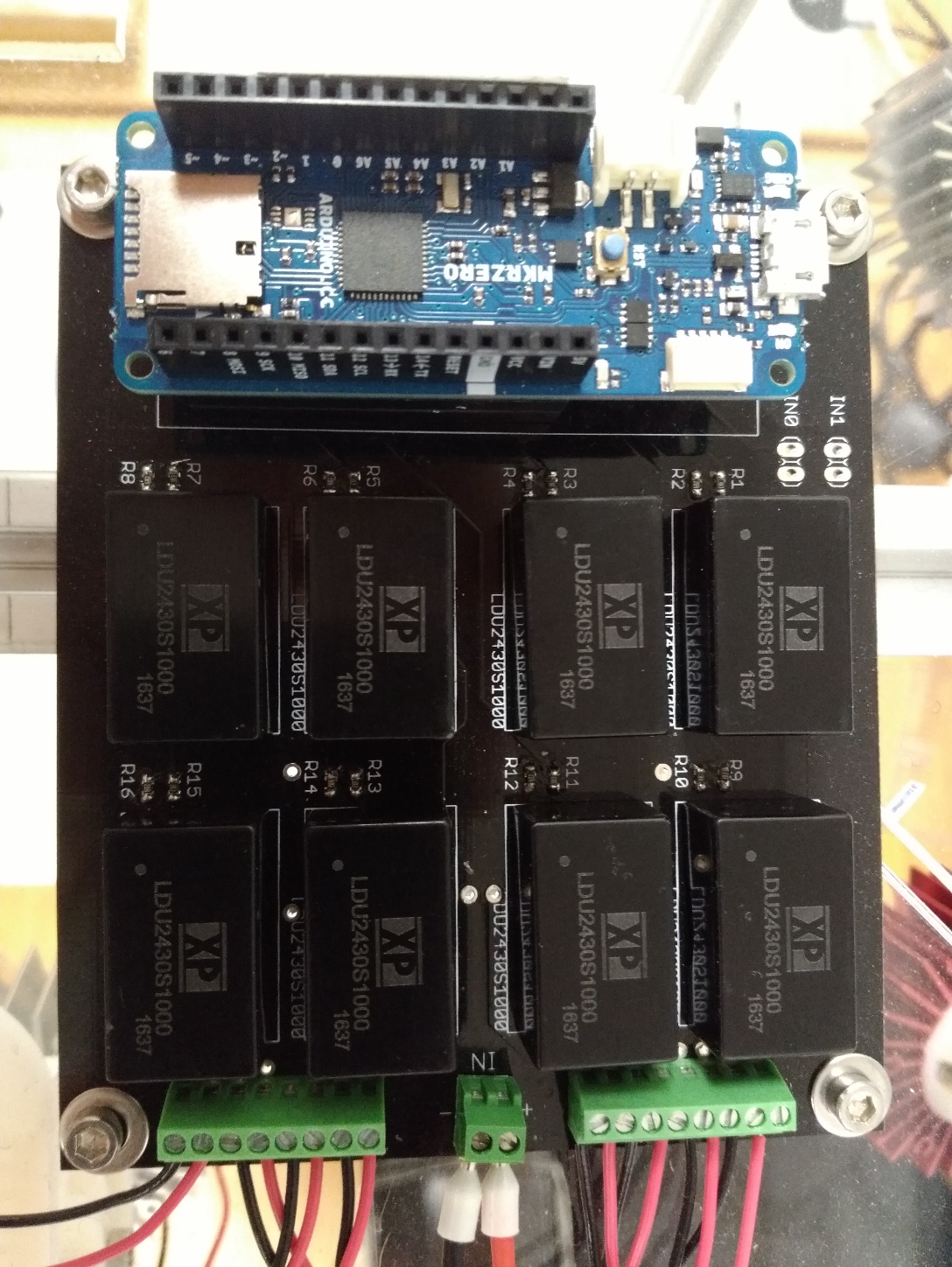


**Figure 5. A fully assembled and operational LED driver board.**


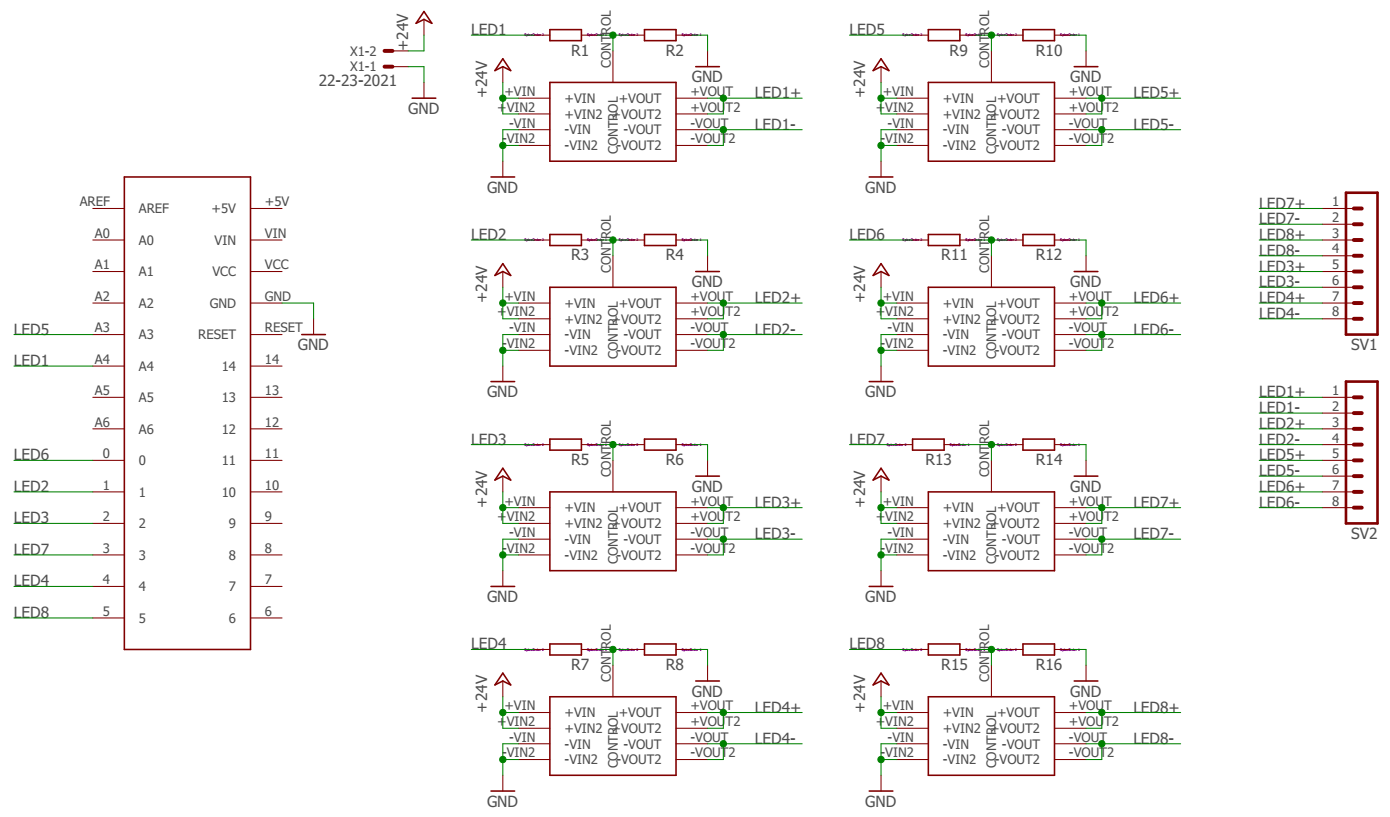


**Figure 6. Schematic of the LED controller.**


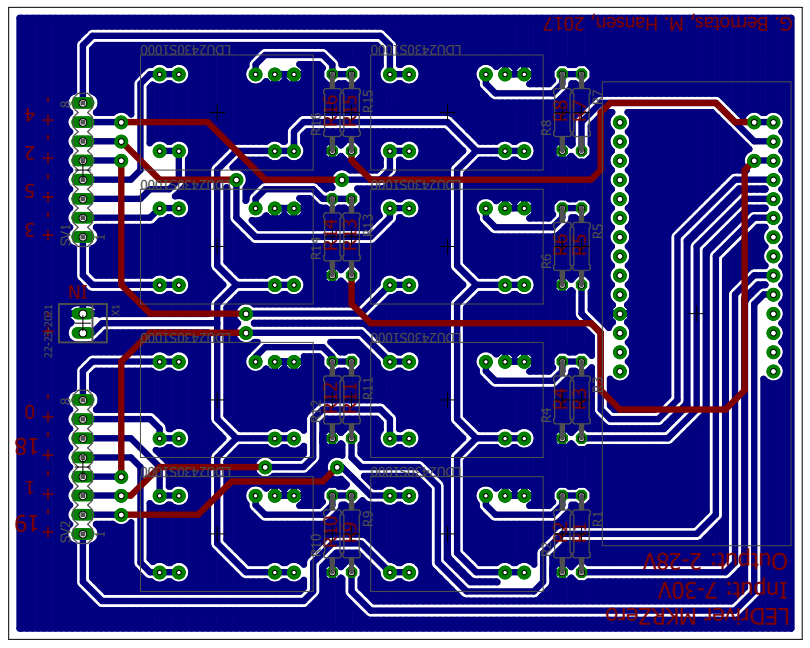


**Figure 7.** **PCB design of the LED controller.**

**Supplementary Information S3.** Formulas.

**Mean Absolute Error (MAE):**

$MAE=\frac{1}{N}\sum_{n=1}^{N} \left| \phi_{actual}\left( n \right)-\phi_{measured}\left( n \right) \right|$ (1)

where n iterates over all samples.

**Mean Relative Error (MRE):**

$MRE=\frac{1}{N}\sum_{n=1}^{N} \mid\frac{A_{actual}-A_{measured}}{A_{actual}}\mid$ (2)

where n iterates over all samples.

**Symmetric Best Dice (SBD) score as in (Scharr, 2016):**

$BD(L^{a}, L^{b})=\frac{1}{M}\sum_{i=1}^{M} \max_{1\leq j\leq N} \frac{2\left| L_{i}^{a}\cap L_{j}^{b} \right|}{\left| L_{i}^{a} \right|+\left| L_{j}^{b} \right|}$ (3)

$SBD\left( L^{ar},L^{gt} \right)=\min_{} \left\{ BD\left( L^{ar}, L^{gt} \right),BD\left( L^{gt}, L^{ar} \right) \right\}$ (4)

where BD is Best Dice, $L^{a}$ and $L^{b}$ are leaf segmentations and $L_{i}^{a}, L_{i}^{b}$ are single object segmentations.

**Foreground-Background Dice (FBD) score as in (Scharr, 2016):**

$FBD= \frac{2\left| P^{gt}\cap P^{ar} \right|}{\left| P^{gt} \right|+\left| P^{ar} \right|}$ (5)

where $P^{gt}$ is the ground-truth of rosette segmentation (union of the whole plant leaves) and $P^{ar}$ is the estimated binary mask of a rosette.

**Rosette compactness:**

$P_{comp}=\frac{A_{rosette}}{A_{convex hull}}$ (6)

where $P_{comp}$ is the rosette compactness, $A_{rosette}$ is the area of rosette and $A_{convex hull}$ is the area of rosette convex hull.

**Rosette circularity (or stockiness):**

$P_{circ}= \frac{4*\pi*A_{rosette}}{P_{rosette}^{2}}$ (7)

where $P_{circ}$ is the rosette circularity, $A_{rosette}$ is the area of rosette and $P_{rosette}$ is the perimeter of a rosette.

**Relative Expansion Rate (RER):**

$\boldsymbol{RER=}\frac{\boldsymbol{ln(}\boldsymbol{A}_{\boldsymbol{t}_{\boldsymbol{2}}}\boldsymbol{-}\boldsymbol{A}_{\boldsymbol{t}_{\boldsymbol{1}}}\boldsymbol{)}}{\boldsymbol{t}_{\boldsymbol{2}}\boldsymbol{-}\boldsymbol{t}_{\boldsymbol{1}}}$ **(8)**

**3D area estimation:**

$A_{3D estimated}= \frac{A_{2D estimated}}{cos\theta}$ (9)

where $A_{3D estimated}$ is the estimated 3D object area, $A_{2D estimated}$ is the projected 2D area, and $\theta$ is the object inclination angle.

**Supplementary Information S4.** Area estimation errors.

*Leaf occlusions and self-occlusions*

One of the major challenges in computer vision is occlusions, as only the visible parts of an object can measured. Plant structure is complex and tends to involve many overlapping leaves (Fig. 1, green circles). Furthermore, leaves can be positioned at oblique angles to the imaging sensor, which limits accurate quantification using top-down camera approaches. This is often called self-occlusion as some of the leaf parts are occluded by the same leaf (Fig. 1, yellow circle). We artificially tilted Arabidopsis rosettes (0 - 45°) to measure the robustness of PS-Plant system for area estimation at different inclination angles. This eventually induced (self-)occlusions within the plant canopy, leading to inaccurate area measurements at higher inclination angles.

| 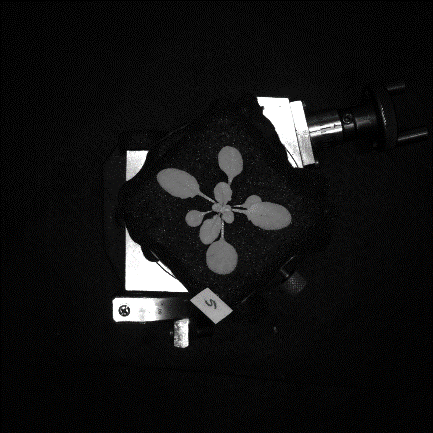  **0º 20º 45º** | 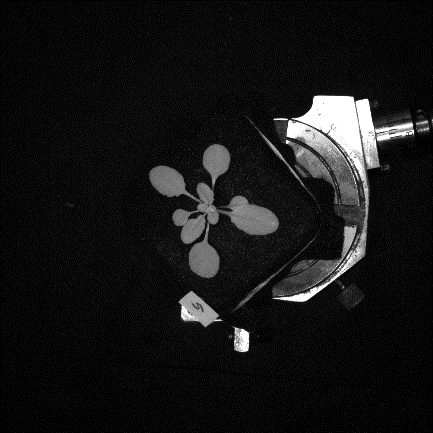 | 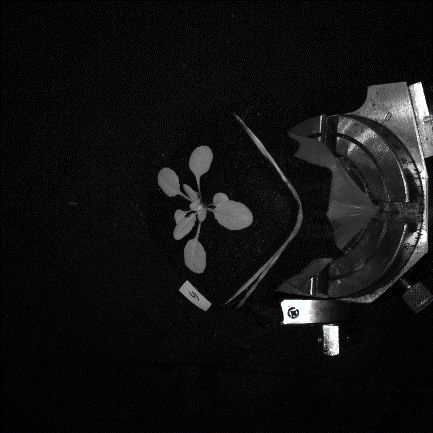 |
| --- | --- | --- |

**Figure 1. Arabidopsis rosettes at three different inclination angles.** Examples of leaf self-occlusion (yellow circle) and occlusion (green circles) are highlighted for a wild-type rosette (23 days after germination).

**Supplementary Information S5.** PS-Plant training data set description.

**Introduction**

Automated leaf segmentation is a challenging area in computer vision. Recent advances in machine learning approaches allowed to achieve better results than traditional image processing techniques [10,11]; however, training such systems often require large annotated datasets [12]. To contribute with annotated datasets and help to overcome this bottleneck in plant phenotyping research, here we provide a novel photometric stereo (PS) training data set **with annotated leaf masks** (<https://datashare.is.ed.ac.uk/handle/10283/3280> - see ‘PS-Plant training data set – metadata.csv’ for training data set details). This data set forms part of work done in the BBSRC Tools and Resources Development project BB/N02334X/1.

**Data description:**

*Plant material and growth specifications*

The data set comprises of 21 *Arabidopsis thaliana* (L. Heynh. Col-0, wild type) plants grown in a growth cabinet at 22^o^C under 150 µmol photons m^-2^ s^-1^ in 12 : 12 hr light : dark cycles. The data set represents plants at varying time intervals (12 to 48 hours) from 11 and 24 days after germination.

*Images description*

Raw data:

Depending on the PS-Plant (Fig. **1A**) rig used [we had two rigs with either four or eight near-infrared (NIR) light emitting diodes (LEDs)], each capture session consisted of four or eight differently illuminated images of a plant tray (named im0 to im3/im7) and a further image capturing ambient light in the scene (named imAmbient). The raw data can be found in the folder “PS-Plant Data”. Each session was saved with the acquisition day (year, month and day) and time (hour, minute and seconds) stamps and the capture mode as follows:

‘*YYYY-MM-DD_hh-mm-ss_MODE*’;

The capture mode refers to the PS-Plant rig from which the images were obtained. For the four NIR LEDs rig, the mode is NIR, and for the eight NIR LEDs, the mode is VISNIR.

All directories in “PS-Plant Data” have a *PSConfig.properties* file – a copy of the capturing PS system configuration and processing information. The properties file included light source direction vectors (x, y and z), data storage and temporary processing locations, light controller communication port, region of interest (in pixels), camera lens focal length and options for data processing. The obtained raw data was processed using PS calculations written in Python and resulted in the generation of a *SNZShadowImAndAlbedo_adaptiveLS.npz* file for every directory. This file contained integrated height information [5], surface normal map, albedo and shadow images.

Processed images:

Each Arabidopsis plant from the raw data was individually cropped by specifying the rosette centroid location and the size of the region of interest. All crops have the same resolution (650 x 560 pixels). The cropped images were named as the parent directory with a ‘*_X*’ suffix, where *X* is the plant number in the tray. The images provided are:

- Annotated image layers: Individual leaf labels for each plant that were manually generated using Adobe Photoshop CS6 (Adobe Systems, CA, USA). Every leaf mask was stored in a separate layer in the format of ‘*Leaf No*’. If the leaf was not visible due to occlusions, an empty layer was included with a title of the missing leaf number. Annotated image layers were stored in a ‘*psd’* file format.
- Ground truth (leaf labels) images: .png images generated from the .psd files (Fig. **1B**).
- Albedo images (Fig. **1C**).
- Surface normal map images (Fig. **1D**).
- Grayscale images: mean of various illumination directions (Fig. **1E**).
- Shadow images (Fig. **1F**).
- Composite images: normals in x and y directions, and albedo (Fig. **1G**).
- Foreground_Background images: a foreground (white) and background (black) image mask (Fig. **1H**).


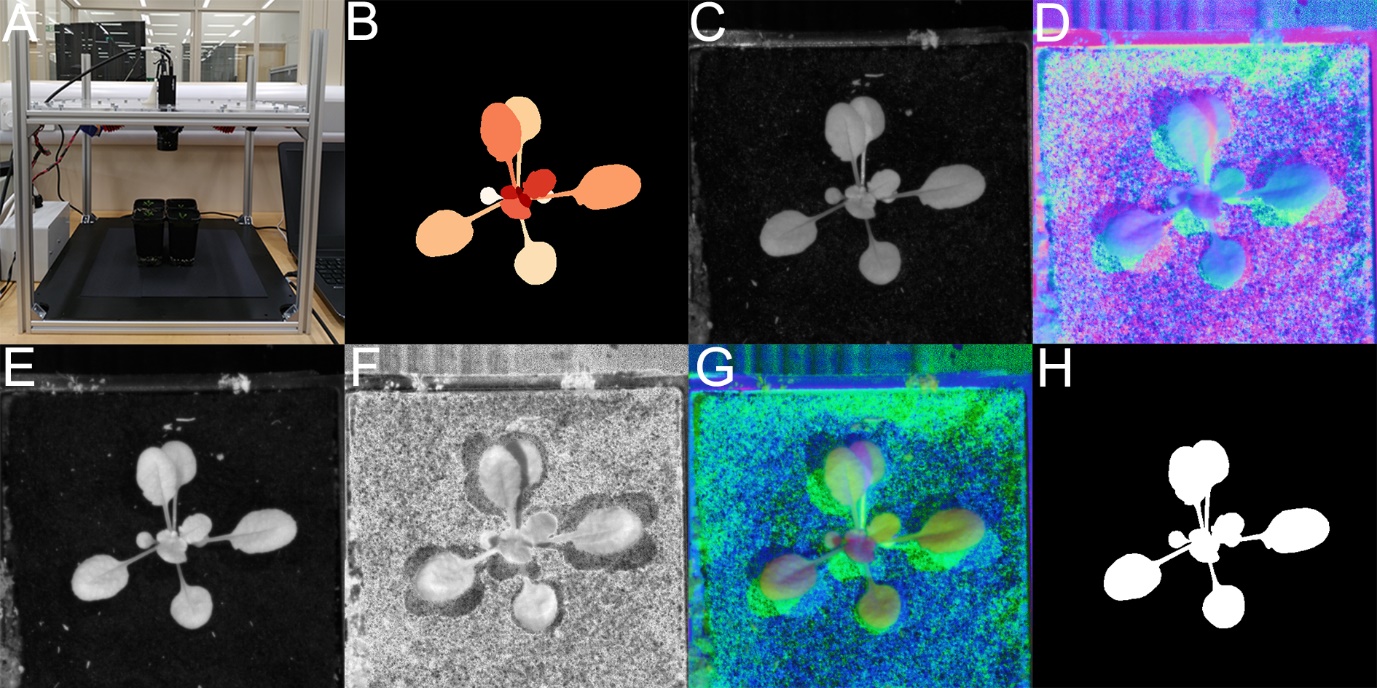


**Figure 1.** PS-Plant rig (A) and examples of the different types of images provided in the PS-Plant data set (B-H).

**References**

1. Woodham RJ. Photometric Method For Determining Surface Orientation From Multiple Images. Opt. Eng. 1980;19:139–44.

2. Iwahori Y, Sugie H, Ishii N. Reconstructing shape from shading images under point light source illumination. Proc. Int. Conf. Pattern Recognit. 1990;83–7.

3. Ahmad J, Sun J, Smith L, Smith M. Improving photometric stereo through per-pixel light vector calculation. Br. Mach. Vis. Conf. 2013;1–12.

4. Argyriou V, Petrou M. Photometric Stereo: An Overview. Elsevier Inc.; 2009.

5. Frankot RT, Chellappa R. Method for Enforcing Integrability in Shape From Shading Algorithms. IEEE Trans. Pattern Anal. Mach. Intell. 1988;10:439–51.

6. Koenderink JJ, van Doorn AJ. Surface shape and curvature scales. Image Vis. Comput. 1992;10:557–64.

7. Tanaka HT, Ikeda M, Chiaki H. Curvature-based face surface recognition using spherical correlation. Principal directions for curved object recognition. Proc. - 3rd IEEE Int. Conf. Autom. Face Gesture Recognit. 1998. p. 372–7.

8. Zhang W, Hansen MF, Smith M, Smith L, Grieve B. Photometric stereo for three-dimensional leaf venation extraction. Comput. Ind. Elsevier B.V.; 2018;98:56–67.

9. Woodham RJ. Determining Surface Curvature with Photometric Stereo. Int. Conf. Robot. Autom. 1989. p. 36–42.

10. Ren M, Zemel RS. End-to-End Instance Segmentation with Recurrent Attention. 2016;

11. Scharr H, Minervini M, French AP, Klukas C, Kramer DM, Liu X, et al. Leaf segmentation in plant phenotyping: a collation study. Mach. Vis. Appl. Springer Berlin Heidelberg; 2016;27:585–606.

12. Giuffrida MV, Scharr H, Tsaftaris SA. ARIGAN: Synthetic Arabidopsis Plants using Generative Adversarial Network. Proc. 2017 IEEE Int. Conf. Comput. Vis. Work. 2017. p. 22–9.

**
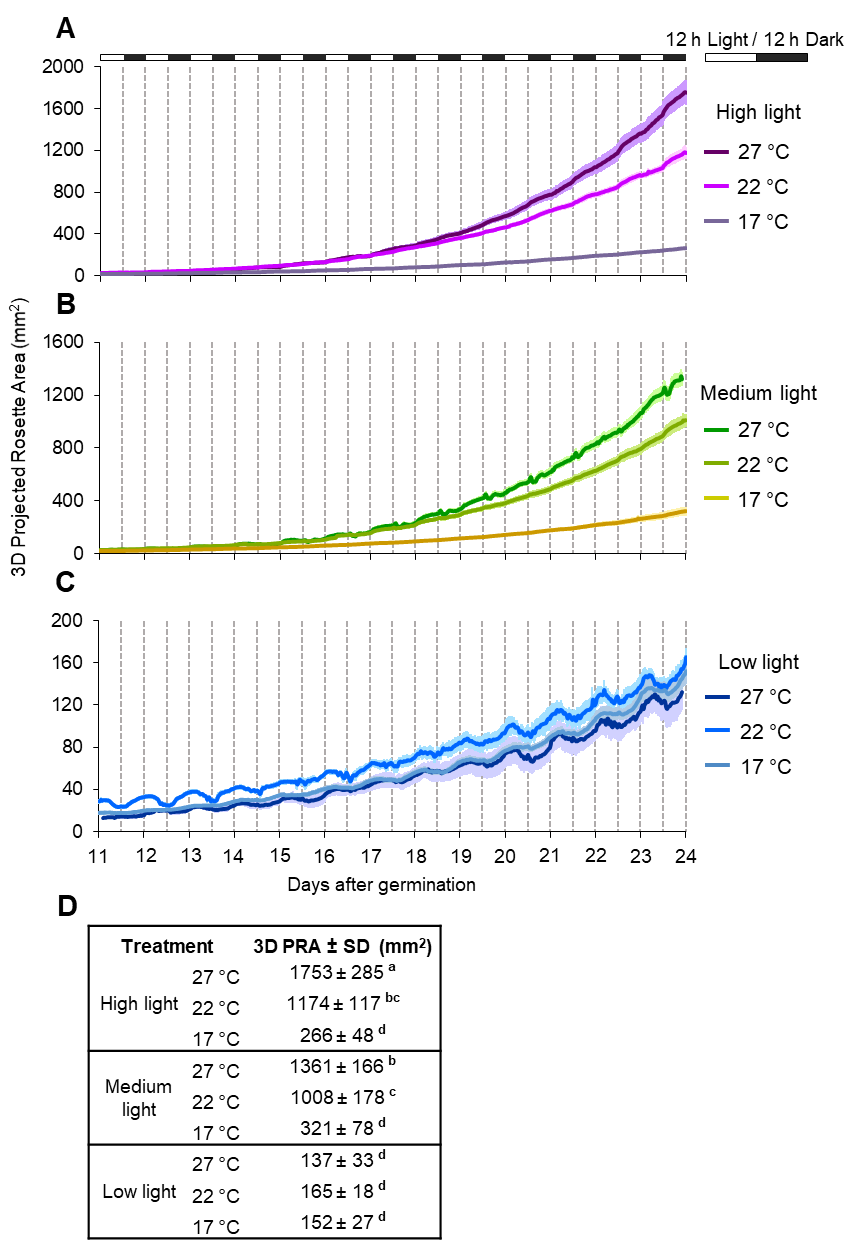
**

**Supplementary Figure S1.** Rosette and individual leaf growth analysis. The mean projected rosette area (PRA) from 3D data for plants in all conditions, separated by light treatment (A-C). Values represent the mean ± SD values of at least three biological replicates. Significant differences in PRA are shown for different treatments for plants at 24 days after germination as determined by ANOVA (p<0.05) followed by Tukey’s HSD tests (D).

**
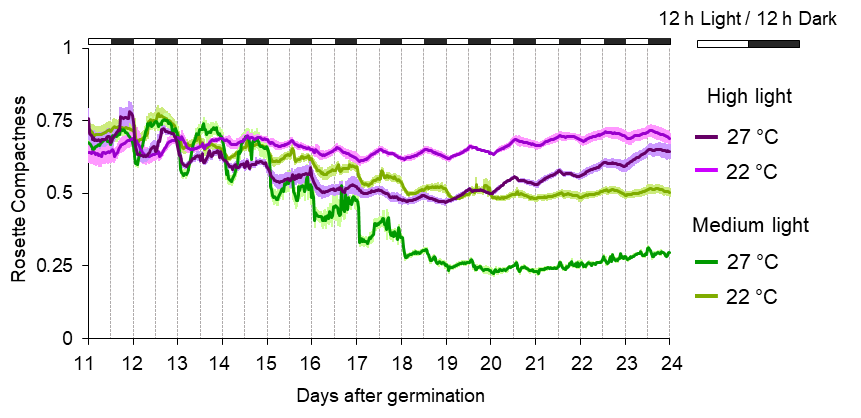
**

**Supplementary Figure S2.** Rosette compactness for plants grown in different conditions. Values represent the mean ± SD values of three biological replicates.


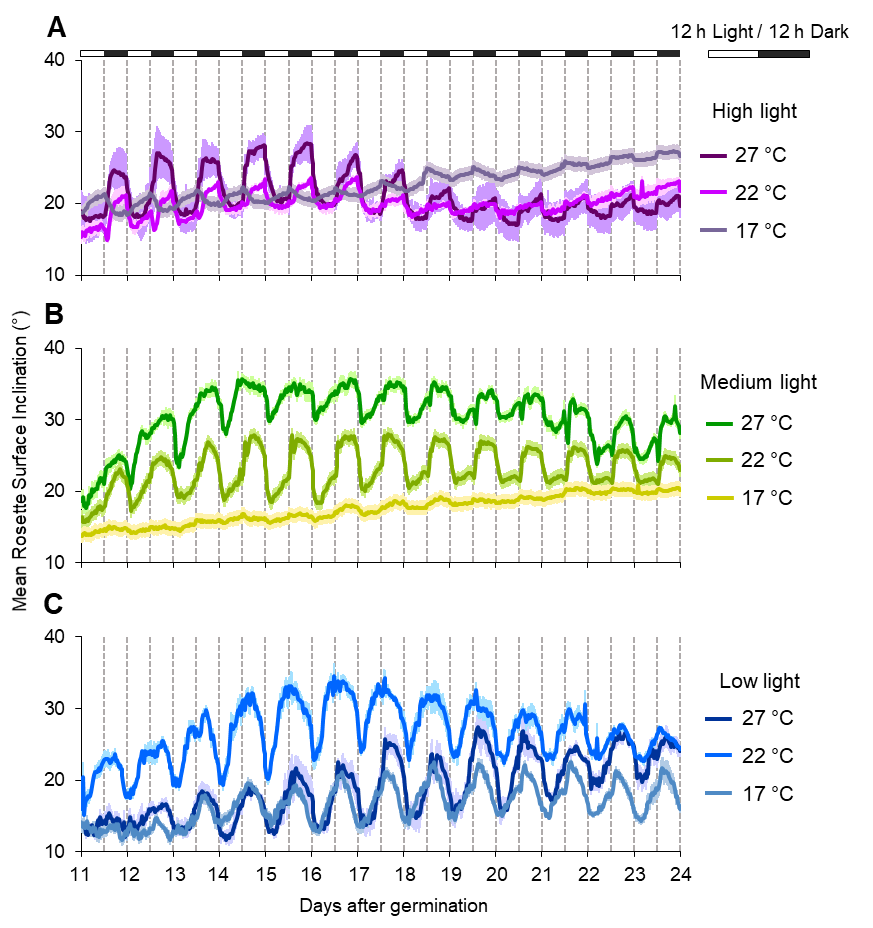


**Supplementary Figure S3.** Mean rosette surface inclinations for all growth conditions separated by light treatment (A-C). Values represent the mean ± SD values of at least three biological replicates.


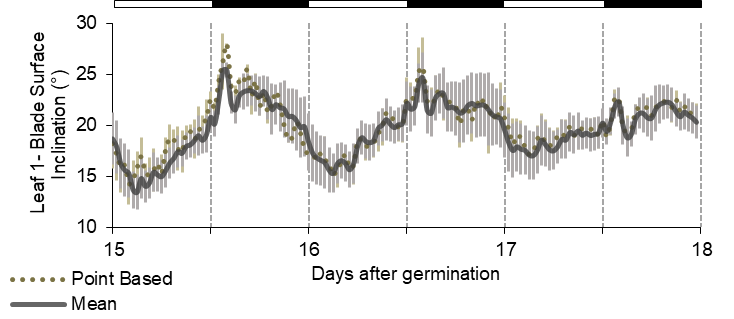


**Supplementary Figure S4.** Estimated leaf inclination of leaf 1 in medium light and 27^o^C (ML-MT) using the mean surface inclination of the leaf blade the blade mean surface inclination or a point-based approach. Error bars represent the ±SD of three separate leaves.
