# Supplementary material for: A photometric stereo-based 3D imaging system using computer vision and deep learning for tracking plant growth
Source: Gigascience. 2019 May 25;8(5):giz056. doi: 10.1093/gigascience/giz056 (PMC6534809; doi:10.1093/gigascience/giz056)
Supplement: giz056_Supplemental_Files [file giz056_supplemental_files.zip › Supplementary Information S6 - PS-Plant protocol 190415.docx]

**Supplementary Information S6.** PS-Plant protocol

The following protocol describes how to image the model plant *Arabidopsis thaliana* using PS-Plant. The protocol consists of four sections: 1) plant preparation, 2) PS-Plant software installation, 3) PS-Plant hardware set up, and 4) PS-Plant software use. For the latter, we have designed a suite of easy-to-use Graphical User Interface (GUI) tools for data acquisition. Together these form an end-to-end analysis framework to obtain dynamic Arabidopsis growth and movement data at the rosette- and leaf-level (Fig. 1). All software was developed in Python. The GUIs were implemented using the cross-platform GUI toolkit Qt [1] and linked to Python software with PyQt binding [2]. The developed PS-Plant software has seven steps that are outlined in Section 4: (4.1) PS data acquisition, (4.2) adaptive light source generation, (4.3) raw PS data processing, (4.4) rosette and (4.5) individual leaf mask generation, and (4.6) leaf instance tracking. The generation of compiled results (i.e. plant trait data) can be done at steps 4.4 and 4.6 to obtain rosette- and leaf-level data, respectively. Most PCs with onboard GPUs can be used to acquire and process PS data (steps 4.1-4.4 and 4.6). We recommend a PC with at least an Intel Core i3 equivalent CPU, and 4 GB of RAM. A GPU with a minimum Compute Unified Device Architecture (CUDA) capability of 3.5 (<https://bit.ly/1Jnzfz8>) is required for the generation of leaf masks (step 4.5) using the Leaf Segmentation GUI.


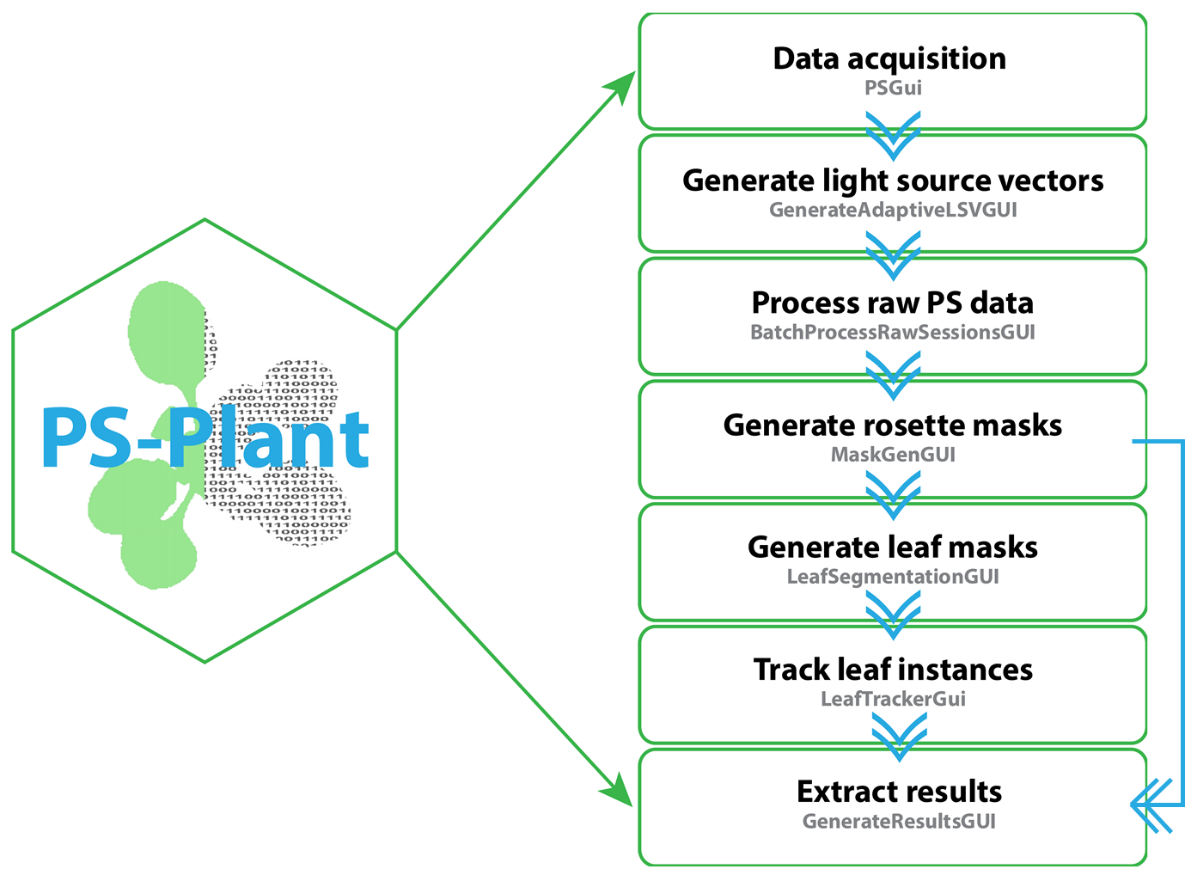


**Figure 1. A high-level workflow representation of PS-Plant software.**

*1. Plant preparation*

The camera on the PS-Plant rig has a 17 x 17 cm field of view, which allows for imaging of up to nine plants placed in 5 x 5 x 5 cm (length x width x height) pots (Fig. 2A). Sow the appropriate number of seeds for your experiment:

- Arabidopsis seeds require stratification to promote uniform germination. Place seeds in a tray of moist compost, cover the tray with a transparent plastic lid to maintain high humidity and place at 4 °C in darkness for three days.
- Transfer the tray with the lid to a growth cabinet with the desired day length, temperature and illumination for seven days to allow seedlings to grow.
- Fill individual pots (as above) with compost and cover the soil with a squared piece of black acrylic felt with a central hole (4.5 - 5 mm) made using a leather hole punch. Reflections from the pot edge may cause noise during measurements. To avoid this, the pot edges can be painted black with matt black acrylic paint, or they can be covered with the acrylic felt fabric and held with a rubber band on the sides (Fig. 2A).
- Transfer one seedling to each prepared pot. Carefully place the seedling in the soil in the central hole. Cover the pots with a transparent plastic lid for one day to allow the seedlings to acclimate. If imaging nine plants, it is good idea to transplant at least 14 seedlings.

* As an alternative strategy, individual seeds can be sown in a prepared pot using a pipette or a toothpick. Seeds can then be stratified in the pot.

*
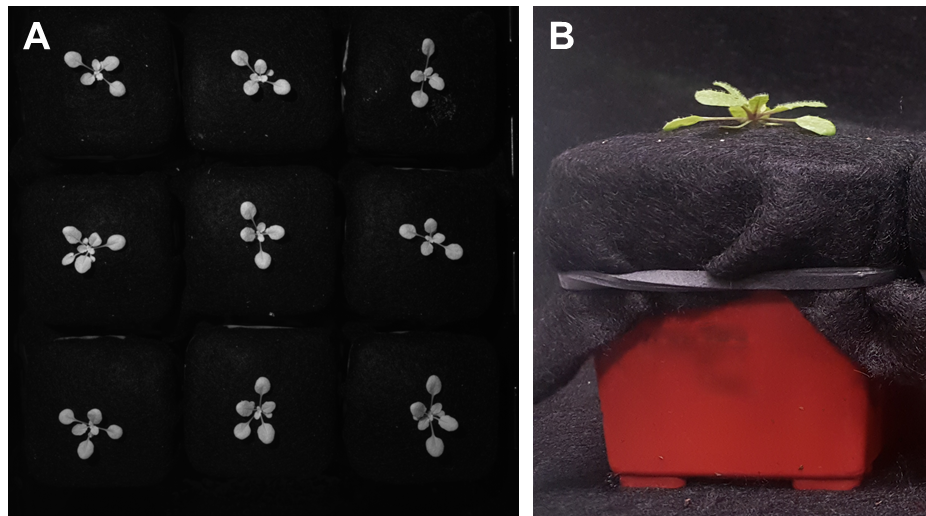
*

**Figure 2. Arabidopsis seedling examples for imaging with PS-Plant.** (A) Nine pots viewed from the top-down camera set-up of the PS-Plant system. (B) An example of an Arabidopsis plant growing in a pot covered with black acrylic felt fabric held with a rubber band.

*2. PS-Plant software installation*

The PS-Plant software RRID number is **SCR_017032**. All PS-Plant software packages are available for download (<https://bit.ly/2EFOk0O>). Installation instructions are outlined below and in the video file ‘Supplementary Data S6 – PS-Plant setup.mp4’ (available on YouTube: <https://youtu.be/7q6ZnJvhx6I>). The computer used to control the PS-Plant hardware must be set up with these software packages and their dependencies. The software installation is as follows:

1. Download the PS-Plant Python packages using ‘Download ZIP’ or using a control client such as TortoiseGit ([https://tortoisegit.org](https://www.google.com/url?sa=t&rct=j&q=&esrc=s&source=web&cd=1&ved=2ahUKEwjRzevWx-jgAhVHQRUIHdHOCB0QFjAAegQIABAC&url=https%3A%2F%2Ftortoisegit.org%2F&usg=AOvVaw3djnsuWm5fluye3fE8-oM6)).
2. Extract the downloaded package.
3. If needed, download and install Python interpreter software of your choice. In this protocol we have used Pyzo (<https://pyzo.org>) and Miniconda (<https://bit.ly/2TBOSy7>).
4. Create a virtual environment for the PS-Plant system by typing in the Anaconda prompt. This can be found in the start menu (**Note:** you may have to run the Anaconda prompt as an administrator):

*conda create –n PS-Plant python=3.5*

1. Create a new shell configuration in Pyzo for PS-Plant software that will use the python executable created by Anaconda. Navigate to ‘Shells’ 🡪 ‘Edit shell configurations’ 🡪 ‘Add config’. You can use any name, for example, ‘PS-Plant_py3.5’. On ‘exe’, select the Python executable file (.exe) from the drop-down list that aligns with the environment name created in (4) above (e.g. c:\ miniconda3\python.exe [v3.5.3, conda]).
2. Start the new PS-Plant shell and install pip (package management system) available dependencies outlined in the ‘Requirements.txt’ by typing:

*pip install -r Requirements.txt*

1. Download pip unavailable packages for camera control (https://bit.ly/2TfaOPd):
   - FlyCapture SDK
   - PyCapture2

If the PS-Plant system used is the same as described in the main text, then ‘Grasshopper3’ should be selected from ‘Product Families’ and ‘GS3-U3-41C6NIR-C’ should be chosen from Camera Models’ (Fig. 3). The operating system installed was ‘Windows 10 64bit’.


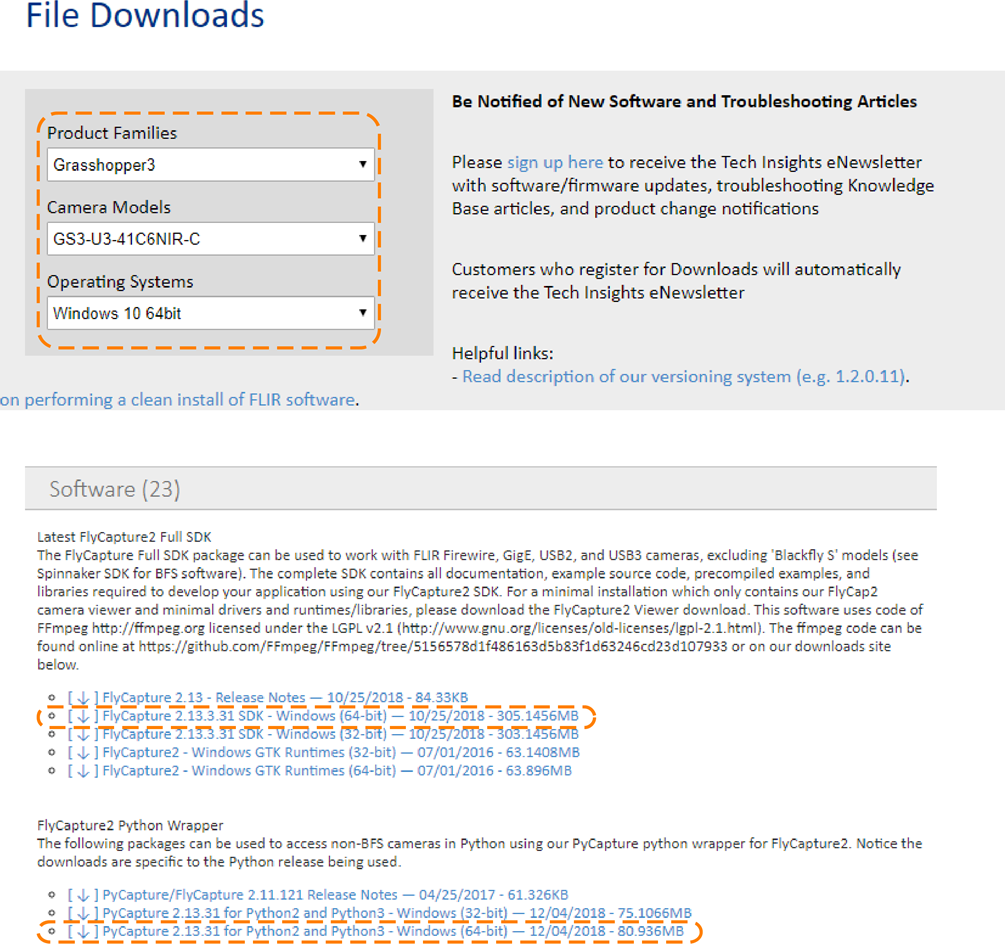


**Figure 3. Downloading FLIR camera software.** After selecting the appropriate entries for Product Families, Camera Models and Operating Systems, select FlyCapture and PyCapture for download from the ‘Software’ drop-down list.

1. Install FlyCapture SDK using the ‘Complete’ option with the recommended installation parameters, except make sure to select the ‘I will use USB cameras’ option (default recommended parameters suggest that USB cameras will not be used). This will install the required camera drivers.
2. Install PyCapture2 with the recommended installation parameters, except at the Python installation step you should provide the path to the created virtual environment from (4) above, which should be ‘…/Miniconda3/envs/PS-Plant’.
3. Download and install Arduino IDE (<https://bit.ly/1R2xniM>) with the recommended installation parameters.
4. Open the installed Arduino IDE and install MKRZero compatible drivers by navigating to ‘*Arduino IDE* 🡪 Tools 🡪 Board 🡪 Boards Manager 🡪 Arduino SAMD Boards (32-bits ARM Cortex-M0+)’ (Fig. 4).

**A B**


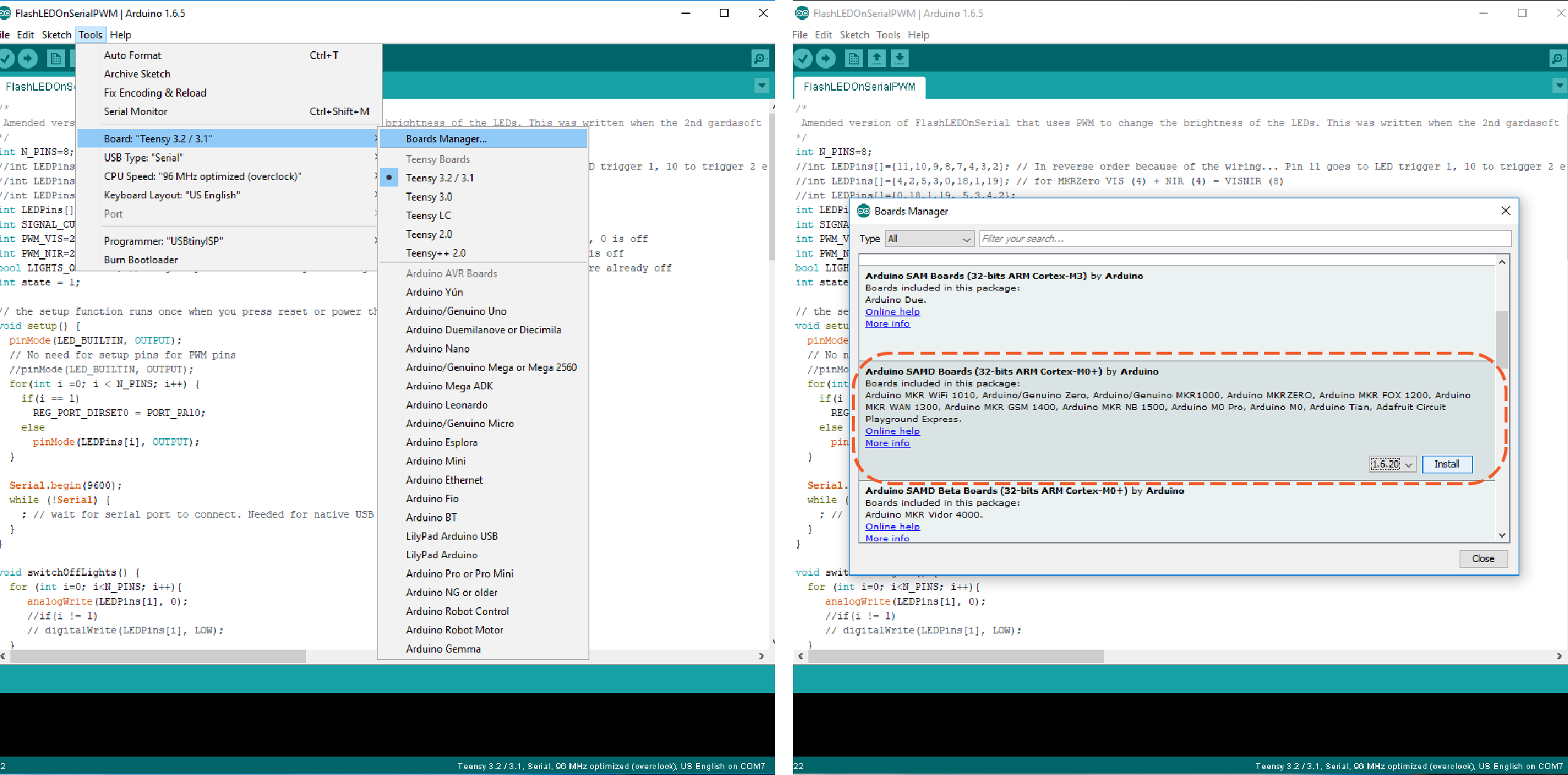


**Figure 4. Arduino IDE and installation of MKRZero drivers.** (A) Navigate to ‘Boards Manager’ and (B) install Arduino SAMD Boards (32-bits ARM Cortex-M0+) drivers.

1. We have provided a test data set for analysis that can be downloaded from the Edinburgh DataShare repository (<https://datashare.is.ed.ac.uk/handle/10283/3279)>. Download and extract ‘Plant test data set.zip’ into a new directory. The data set is comprised of Arabidopsis plants (11-13 days after germination) grown at 25 °C under 150 µmol photons m^-2^ s^-1^ of white light (for details see ‘PS-Plant test data set_metadata.csv’ in the .zip file).

*3. PS-Plant hardware setup*

Once the PS-Plant software is installed successfully, you are required to integrate PS-Plant hardware (outlined in Supplementary Information S2) with the installed software. Integration can be achieved as follows:

1. Connect the Grasshopper3 camera to one of the 3.0/3.1 USB ports available in the PC with the installed software.
2. Connect the USB cable of the bespoke LED controller (Arduino) to one of the USB ports available in the PC.
3. Open the installed FlyCapture software (Windows 🡪 FlyCap2), which should show the PS-Plant field of view. If the software does not show the field of view (it may say ‘streaming problem’), close the software and disconnect and reconnect the USB cables. If using a NIR filter, the field of view will be very dark (or appear completely black) - you will need to switch on the NIR lights to view objects in the field of view (e.g. plants) and adjust the focus.
4. Open the *Arduino IDE* and upload the provided Arduino code (.../PS-Plant-Framework/ Arduino/FlashLEDOnSerial) on the Arduino microcontroller available as part of the bespoke LED controller. There are a few methods to upload the provided code:

- Click on the right arrow icon (Fig. 5A).
- Press key combination ‘Ctrl + U’.
- Navigate to Sketch 🡪 Upload (Fig. 5A).

Open Arduino ‘Serial Monitor’ (top-right corner) and type in the number ‘8’ and click on ‘send’ (Fig. 5B). This should light two of the NIR LEDs, which will be used to focus and adjust the camera parameters.

**A B**


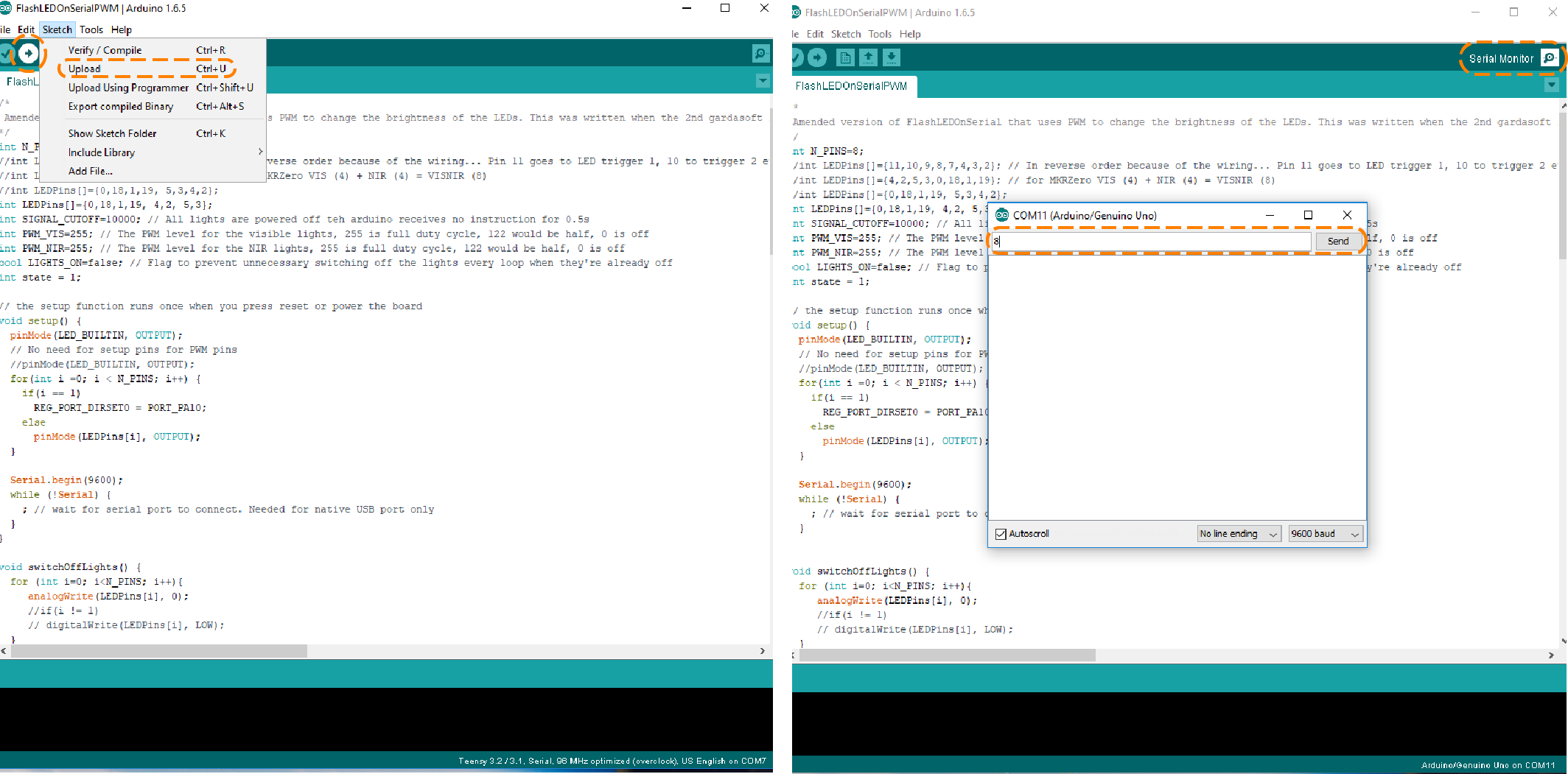


**Figure 5. Navigating Arduino IDE.** (A) The outlined Arduino upload options and (B) communication via **‘**Serial Monitor’.

1. Adjust the camera parameters in the FlyCapture SDK (we used a frame rate of 14, shutter speed of 50 ms) and camera lens parameters (we used focal length of f/5.6, aperture of 0.2) to obtain focused images of appropriate brightness (Fig. 6A, B). If appropriate parameters are chosen, the viewer should see the subtle details of the canopy surface when viewing an Arabidopsis rosette. We have found that the best performances are obtained when pixels belonging to the plants have intensity values in the range of [200, 230] (the camera’s upper limit is 255) (Fig. 6C). The pixel intensity values can be obtained from the FlyCapture SDK by hovering the cursor above the pixels of interest. The pixel coordinate pair (x, y) and intensity value will be displayed in the bottom-left corner. **Note:** these camera setting parameters are only suggestions and may need to be optimised for your imaging environment.


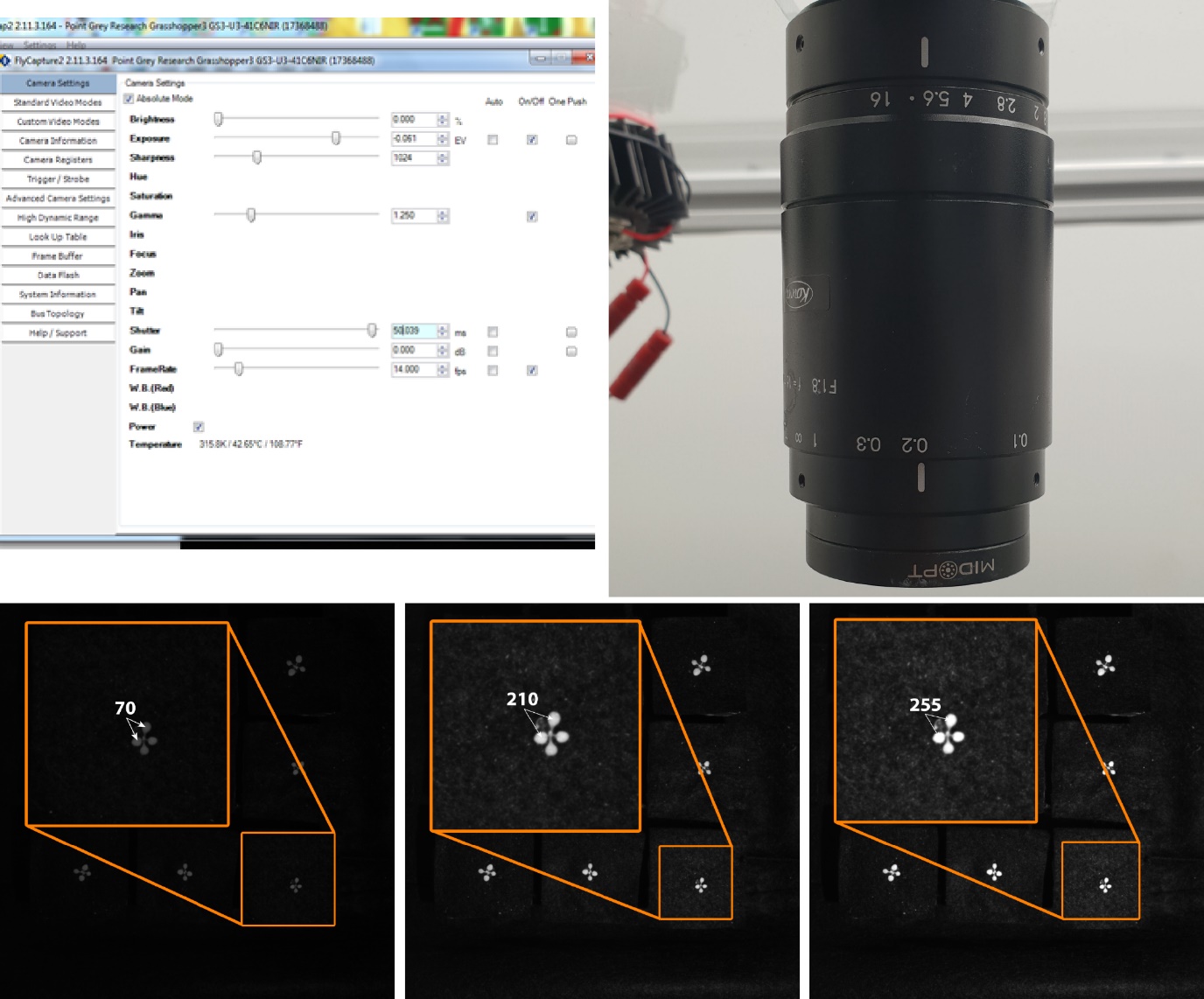


**A B**

**C**

**Figure 6. Setting up the PS-Plant camera system.** (A) Adjust the camera framerate and exposure time in the FlyCapture SDK and (B) the camera lens parameters to obtain (C) focused images with appropriate pixel intensity values. The images on the left and right indicate pixel values that are too low and too high, respectively.

**Too bright**

**Ideal**

**Too dark**

1. Open the Arduino IDE ‘Serial Monitor’ (top-right corner (magnifying glass icon)) and type the number ‘9’ to the Arduino. This should switch off the NIR LEDs. You can confirm that the LEDs are off if you are not able to see the plants in the camera field of view anymore.

*4. PS-Plant software use*

4.1.1 PS data acquisition

When both hardware and software has been successfully set up, the PS-Plant system can be used for data acquisition (i.e. image capture), processing and generating results. There are a few methods to open the PS-Plant GUI-based software packages. Open the provided script in Python interpreter (e.g. Pyzo) and do either of the following:

1. Press ‘F5’.
2. Press the key combination ‘Ctrl + Shift + E’.
3. Navigate to the top of the Pyzo panel 🡪 ‘Run file as script’.

The software package PSGUI is used for PS data acquisition (i.e. PS image capture). PSGUI can also be used for processing captured or previously captured PS images. For data acquisition, the user must first set up the PSConfig.properties file found in the downloaded PS-Plant directory (see Section 2 ‘PS-Plant software installation’, step 2). This file is the configuration used for PS data acquisition and contains:

1. Light source position vectors (x, y and z).
2. Data storage and temporary processing locations.
3. The light controller communication port.
4. Region of interest (in px).
5. Camera lens focal length.
6. Options for data processing; i.e. to show results, export 3D data, crop images, subtract ambient image or apply light compensation technique (0 for false (no), and 1 for true (yes)).

You can adjust these properties according to your PS-plant set up. Pay particular attention to points 2, 3 and 5. Save changes to the PSConfig.properties file. To identify the light controller communication port (3), open Arduino and go to: Tools 🡪 Port: ‘COM**X**’ (Arduino MKRZero).

After the PSConfig.properties file is set up, open PSGUI. Single instances of PS image data can be acquired in PSGUI by clicking the ‘Capture’ button (top left). The user also has a control of LED illumination arrangement, which is accessed via ‘Mode’ section:

- **VIS** – four vertical and horizontal LEDs.
- **NIR** – four diagonal LEDs.
- **VISNIR** – all eight LEDs.

After a successful data acquisition, a new directory titled with the current timestamp and acquisition illumination mode (e.g. VIS, NIR or VISNIR) will be created in the archive directory specified in the PSConfig.properties file (the default path is ‘D:\PSRigData\ArchivePath’). A copy of the PSConfig.properties file will be saved in each acquisition directory (Fig. 7). PSGUI will also display an integrated 3D [3] object surface in an interactive screen on the left side, while the surface normal directions in x, y and z and an albedo image are displayed on the right side (Fig. 8).


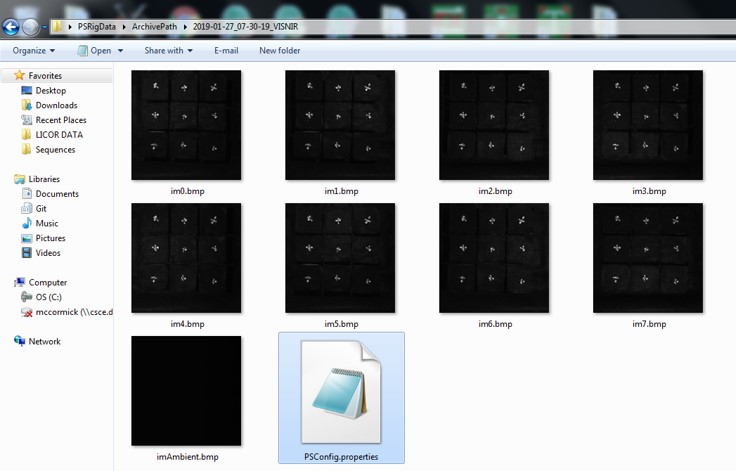


**Figure 7. Example of the directory contents for a single capture event following successful PS data acquisition.** PSGUI generates a new directory for each capture, which contains the PS images and a copy of the PSConfig.properties file.


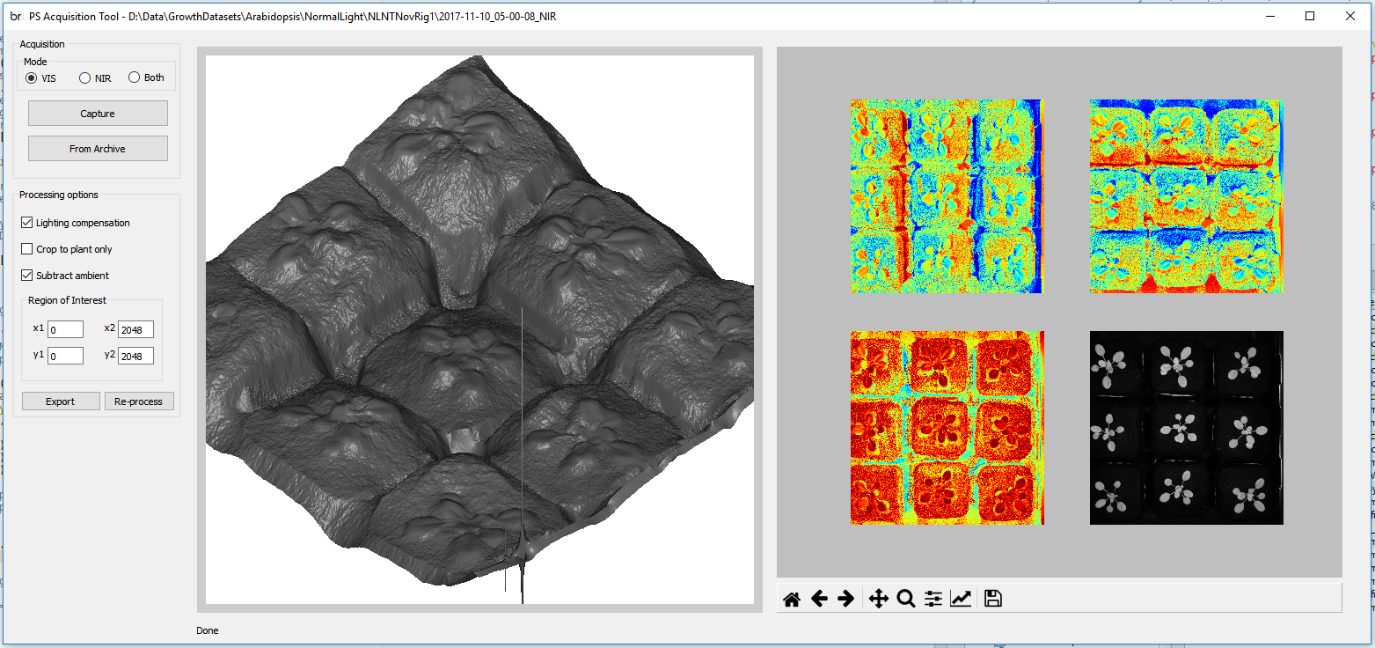


**Figure 8. Visualizing PS data in PSGUI.** PSGUI enables capturing and processing of new data, or processing of data acquired in a previous session (by clicking ‘From Archive’). The GUI displays an integrated 3D surface on the left. On the right the software shows the surface normal map directions in x (top left), y (top right) and z (bottom left), and the albedo (bottom right).

4.1.2 Automated PS data acquisition

Once you have tested that the PS-Plant system is acquiring data (as in 4.1.1), and that the acquired images represent your requirements (e.g. all plants are visible and clear with appropriate exposure levels), you are ready to set up the system to automate data capture over time (e.g. a growth experiment). Here you will use the Windows Task Scheduler (**Note:** specific for Windows - Linux and iOS users may explore scheduler alternatives, such as cron), (<https://bit.ly/2ILVC7j>) to set up recurring schedules for repeated acquisition:

1. Open the Windows Task Scheduler by typing ‘Task Scheduler’ in the start menu.
2. Select ‘Create Task...’ on the right-hand side and fill in as shown below:


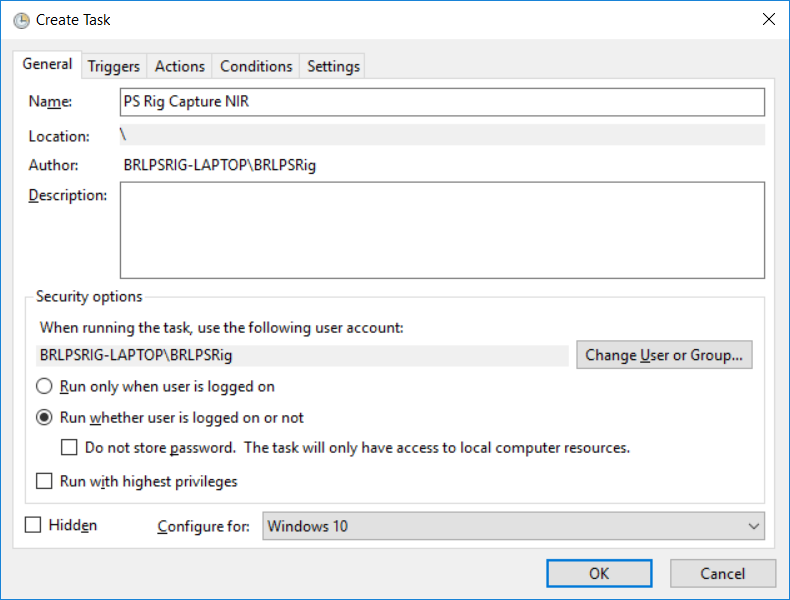


1. In the ‘Triggers’ tab (below), create a new trigger selecting ‘Daily’, set the start date and start time (as required), and leave ‘Recur every 1 day’. Set ‘Repeating task every’ as often as required and ‘for a duration of’ for the length of the experiment (below we have chosen every ‘1 hour’ for ‘1 day’):


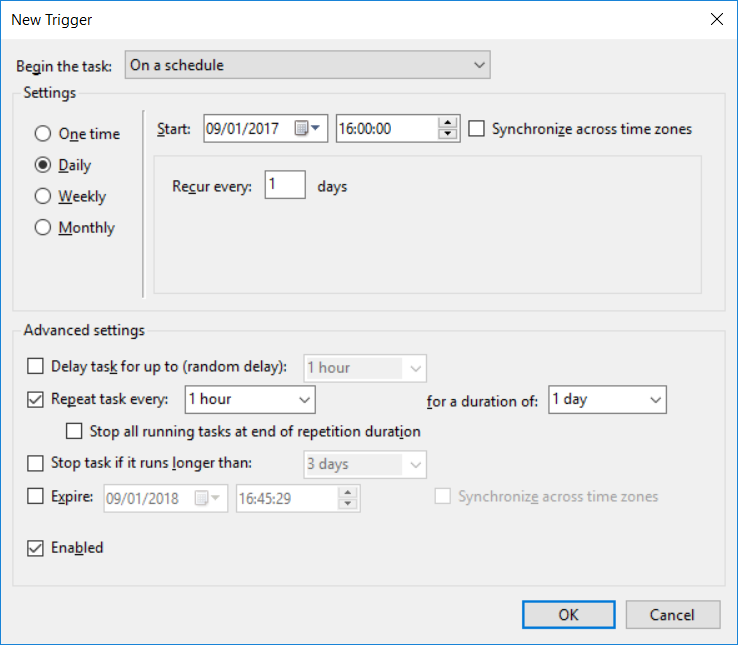


1. In the ‘Actions’ tab (below), create a new action and enter the following:

- **[Program/Script]** Enter the location of the Python executable in the created PS-Plant environment (see Section 2 ‘PS-Plant software installation’, step 4 [e.g. ‘…/Miniconda3/envs/PS-Plant/python.exe’]).
- **[Add arguments]** Enter *‘*AcquirePSImages.py’ followed by ‘VIS’, ‘NIR’ or ‘VISNIR’ to indicate the data capture mode (separated by a space).
- **[Start in]** Enter the directory path where the PS-Plant software and scripts are located (see Section 2 ‘PS-Plant software installation’, step 2).


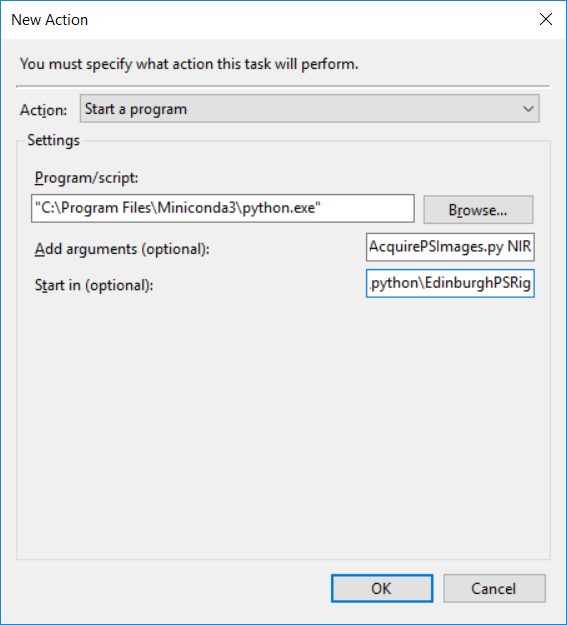


1. Finally, save the created task and enable it to run by right-clicking on the task and choosing ‘Enable’.

4.2 Generating adaptive light source vectors

Once you have acquired an image data set, you will need to generate an adaptive Light Source Vector (LSV) file. This step is used to correct for the PS assumption of the point light source; i.e. that the distance of the light source from the object tends to infinity (see Supplementary Information S1). This step does not need to be completed to progress towards trait data generation, but we recommend it to yield more accurate results. Generation of an LSV file is done only once for a data set. The same LSV file can be used for subsequent data sets as long as the PS-Plant rig setup does not change.

The software package GenerateAdaptiveLSVGUI allows users to generate the LSV file. Open and execute (e.g. you can press ‘F5’) GenerateAdaptiveLSVGUI on Pyzo and the GUI will open (Fig. 9). The parameters that need to be entered in this GUI are outlined in Table 1.


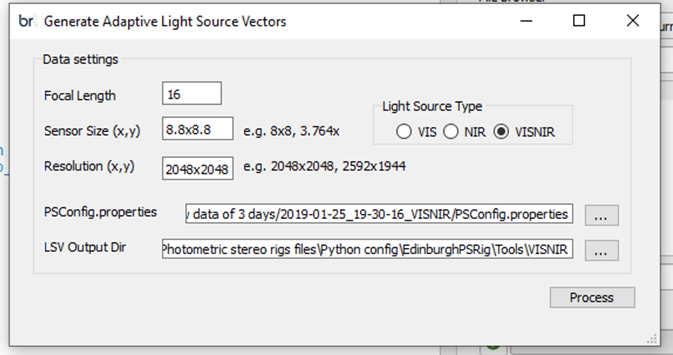


**Figure 9. Screen display of the GenerateAdaptiveLSVGUI for generating adaptive light source vectors.**

**Table 1. Parameters for** **GenerateAdaptiveLSVGUI.**

| Focal Length | The focal length of the camera lens in mm (e.g. 16 mm). |
| --- | --- |
| Sensor size | The sensor size of the camera in mm, specified as width x height (e.g. 8 x 8). |
| Resolution | The camera’s resolution in pixels, specified as width x height (e.g. 2048 x 2048). |
| PSConfig.properties | Select the PSConfig.properties file in any of the captured sessions to load the PS-Plant configuration settings (Fig. 7). |
| LSV Output Dir | Enter the directory where the LSV file will be saved. The naming format of the LSV file is:  AdaptiveLSV_r<resolution>_ss<sensor_size>_fl<focal_length>.npz |
| Light Source Type | Select which light source configuration type was used. |

**Note:** We provided LSV file ‘1.LSV’ in the test data set (i.e. ‘PS-Plant test data set\1.LSV’).

4.3 Raw PS data processing

Image data for each time point in a data set is saved in separate directories. To batch process the generation of PS outputs for all directories in a data set the software package BatchProcessRawSessionsGUI (Fig. 10) is used. This GUI generates a .npz file (named ‘SNZShadowImAndAlbedo.npz’) for every directory (Fig. 11), which includes processed PS data for all subsequent analyses (i,e. a surface normal map, height information, shadow and albedo images). The parameters that need to be entered in this GUI are outlined in Table 2. Here we refer to a single data acquisition directory as a ‘session’.


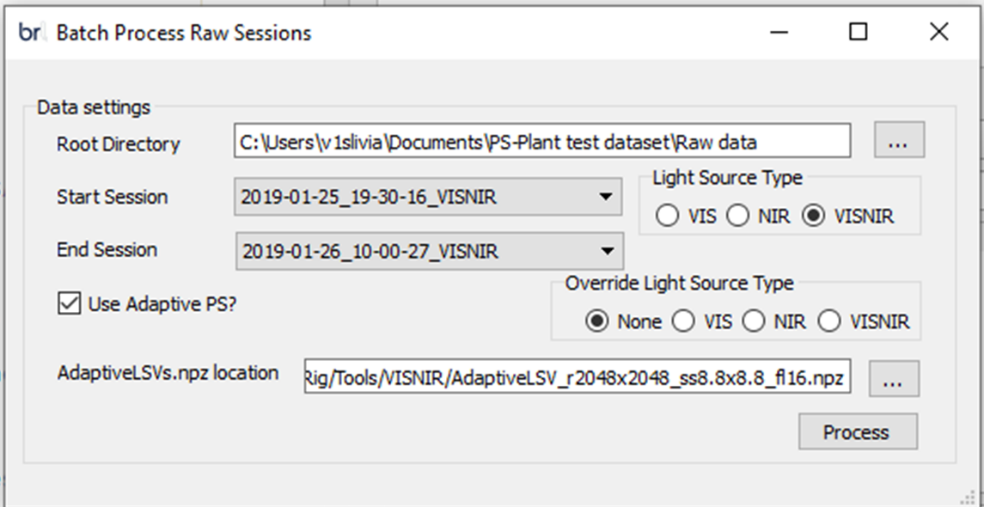


**Figure 10. Screen display of the BatchProcessRawSessionsGUI for processing PS data for every directory**

**Table 2. Parameters for BatchProcessRawSessionsGUI**

| Root Directory | The top-level data directory containing all the sessions (e.g. growth experiment data sets). |
| --- | --- |
| Start Session | Session to start processing from. |
| End Session | Session to stop processing with. |
| Light Source Type | VIS, NIR or VISNIR (select depending on data needed to process). **Note:** The drop-down lists for ‘Start Session’ and ‘End Session’ will update when this selection is altered. |
| Use Adaptive PS? | If selected, an adaptive light source vector (LSV) file will be used (we strongly recommend using this). |
| Override Light Source Type | Allows user to change the lighting source type of the data acquisitions. |
| AdaptiveLSV.npz location | This option will only be available if ‘Use Adaptive PS?’ is selected. Provide the location of the generated LSV file (Step 4.2). |


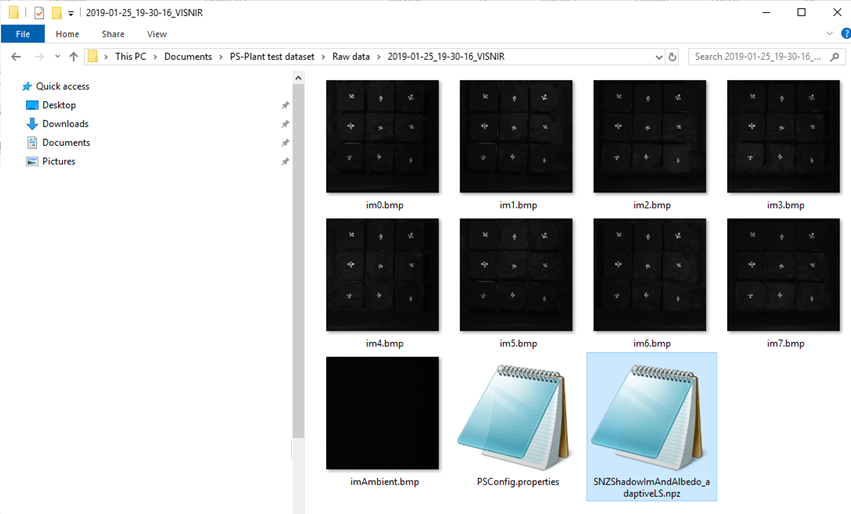


**Figure 11. Example of the directory contents for a single capture event after PS data processing with BatchProcessRawSessionsGUI.**

After data processing with BatchProcessRawSessionsGUI, each of the selected PS sessions will have a ‘SNZShadowImAndAlbedo.npz’ file containing the PS outputs. **Note:** the provided test data set (<https://datashare.is.ed.ac.uk/handle/10283/3279>) has already been processed so all directories in the ‘raw data’ folder contain a .npz file.

4.3.1 Generating a ‘regions of interest’ (ROI) file and identifying plant coordinates

The next step is to determine where your plants are in the image; i.e. the regions of interest (ROIs). The ROIs are defined as rectangular areas of a chosen size. The ROI file is a text file where each line will correspond to the coordinates of these areas for each plant. An example of the ROI file is given for the test data set (named ‘9plantsroi.txt’ in ‘PS-Plant test data set\3. ROI’) and the values can then be changed accordingly depending on the data set. The naming order of an image with nine plants is from top left to bottom right. The numbering starts at zero, so plants are labelled from 0 to 8 (Fig. 12A). Thus, the first line of ‘9plantsroi.txt’ corresponds to the coordinate of plant 0 and so on (Fig. 12B). Each line contains four numbers that correspond to:

- The top left coordinate x.
- The top left coordinate y.
- ROI width.
- ROI height.


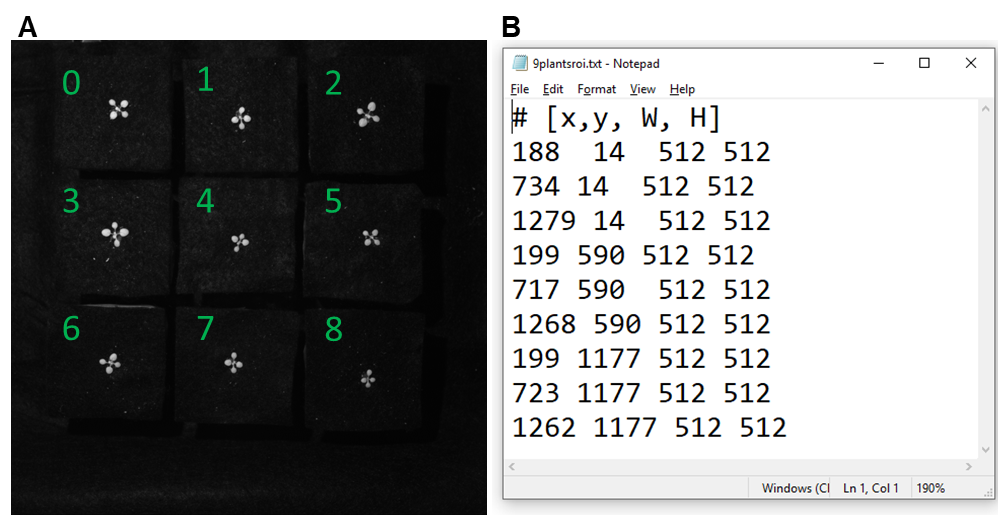


**Figure 12. The order of plants using PS-Plant software.** (A) When there are multiple plants in an image, the plants are numbered from top left to bottom right, starting from 0. (B) An example of an ROI file containing the coordinates for nine different plants.

The coordinates of plants in a given growth experiment can be obtained using the Python IDE (Integrated Development Environment) or FlyCapture SDK. For the Python IDE, copy the path for the captured session of the data set. We recommend using the last capture (i.e. the capture where the plants are largest to ensure that the whole plant will fit in the ROI. Paste the path to ‘GetRoi.py’ after ‘pth=’ (see below). Make sure to add an extra backslash after each backslash in the copied path.


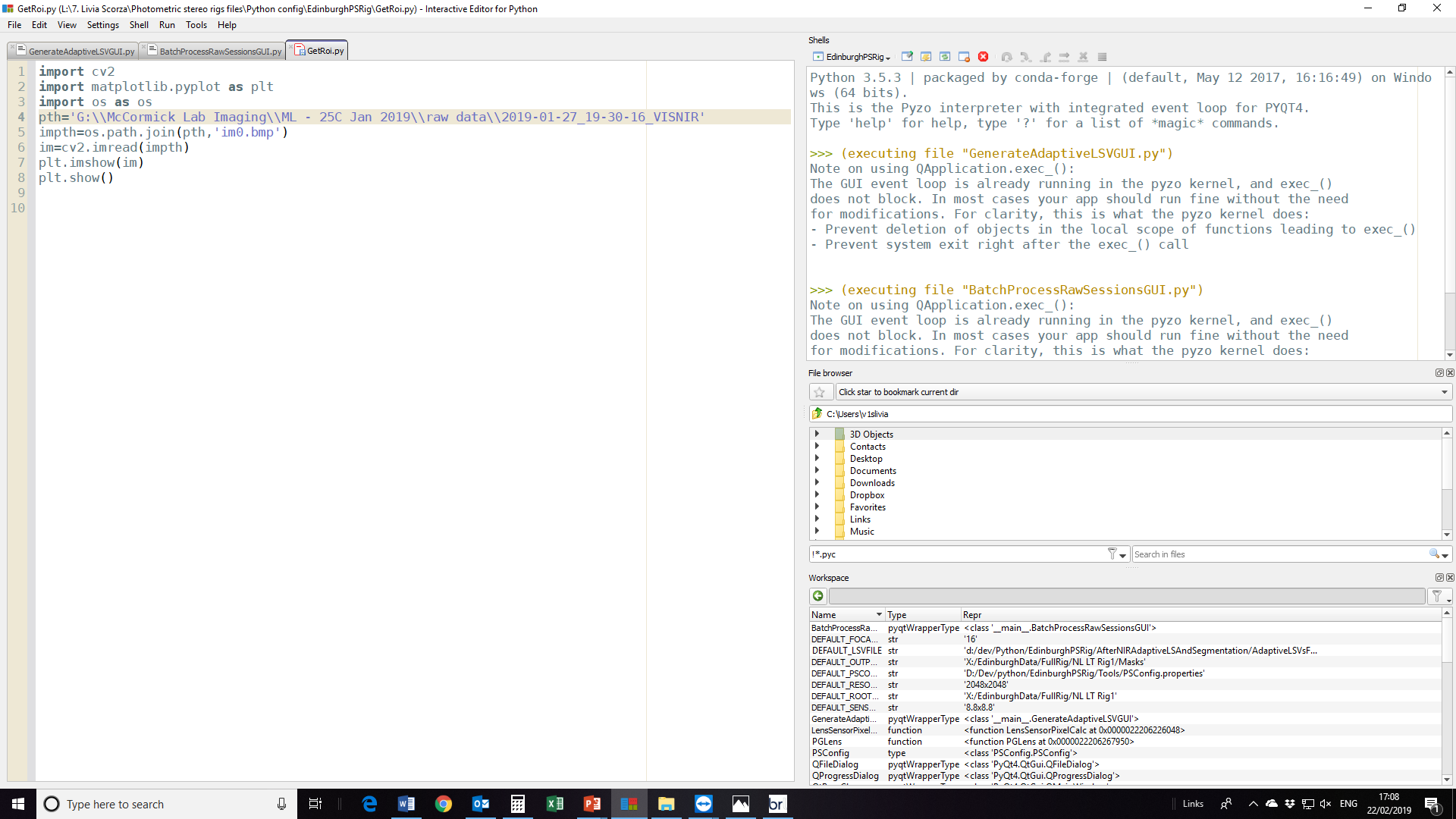


After running the script, the first image of the chosen captured session will open (i.e. ‘im0.bmp’ (Fig. 11)). Placing your cursor on the image will show the x and y coordinate pair values at the bottom-left of the image (see the blue circle in the image below). This will change according to the location of the cursor. Locate the top-left corner of the area of the first plant and copy the coordinate values to the ‘9plantsroi.txt’ file. Identify the lower-right ROI coordinate pair and use them to identify ROI width and height. In this example, a square ROI size of 512 x 512 px was chosen, so the first text file line, which corresponds to the plant 0, will be ‘188 14 512 512’ (Fig. 12B). This must be repeated for all other plants in the appropriate order (Fig. 12A, B). **Note:** the ROI filename does not have to be ‘9plantsroi.txt’ and can be changed without causing problems with subsequent analyses.


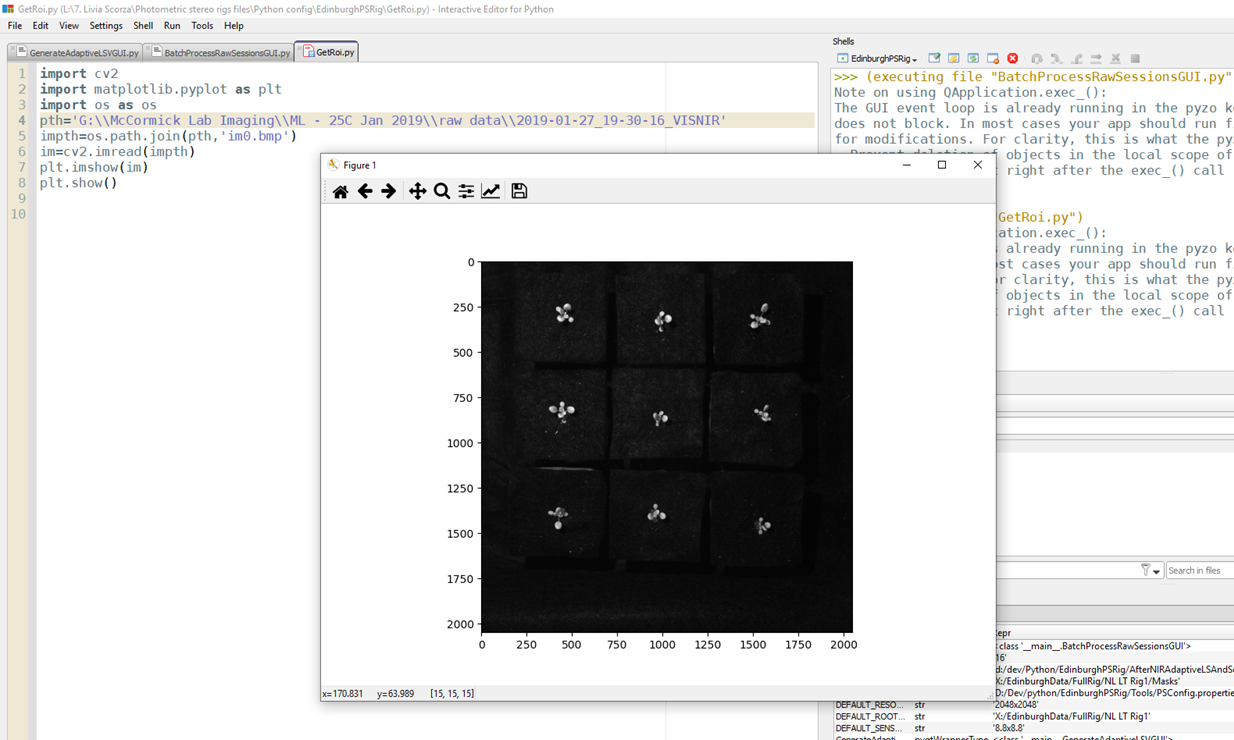


A similar procedure can be implemented using FlyCapture SDK. However, this method can only be used to determine ROI for the current (live) field of view. As above, placing the cursor on the image in FlyCapture SDK will show the x and y coordinate pair values at the bottom-left of the image. This will change according to the location of the cursor. Record the top-left ROI corner coordinate pair and identify the width and height of each ROI. **Note:** The provided ROI file (‘9plantsroi.txt’) already has the correct coordinates for the nine plants in the test data set.

4.4 Generating rosette masks

The rosette masks are binary (black background and white foreground) images that are generated using the MaskGenGUI software package. These masks will be used for rosette trait data generation and in subsequent data processing steps. The segmentation parameters in this GUI are adjustable (i.e. albedo threshold value, minimum object area and filter size). The effect of modifying these parameters can be seen using the ‘Preview’ button. With the optimized segmentation parameters, the GUI can be set to run for a set of directories with the same parameters and ROI. If necessary, erroneous masks can be edited afterwards using an external image editor (e.g. Adobe Photoshop, GIMP). MaskGenGUI can also create three separate directories containing the albedo, grayscale and composite (three-channel images: surface normal direction in x and y, and albedo) images for each plant. These images are cropped using the same ROI provided for the rosette mask generation and are useful for erroneous rosette mask editing, as they can be overlapped with the corresponding mask in an image editor to facilitate precise mask editing. The albedo, grayscale and composite images can also be used as an input for leaf segmentation step.

Here again we refer to a single data acquisition directory as a ‘session’. The MaskGenGUI is shown in Fig. 13. The parameters that need to be entered in this GUI are outlined in Table 3.

**
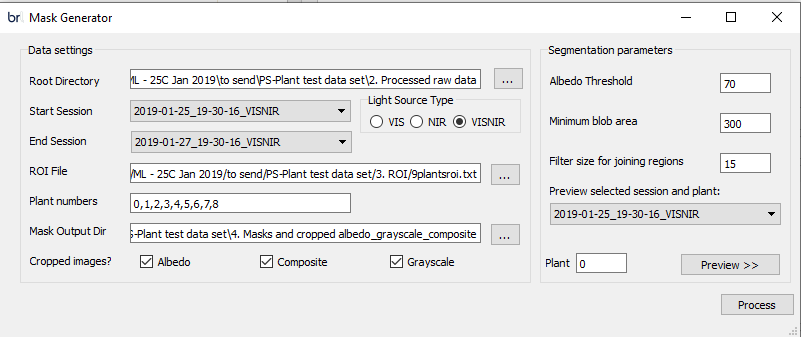
**

**Figure 13. Screen display of the MaskGenGUI used for generating a rosette mask and an albedo, grayscale, and composite image for each plant.**

**Table 3. Parameters for MaskGenGUI.**

| **Root Directory** | The top-level data directory containing all the sessions. |
| --- | --- |
| **Start Session** | Session to start processing from. |
| **End Session** | Session to stop processing with. |
| **ROI File** | The text file containing the ROIs for each plant. |
| **Plant numbers** | Comma separated list of plants (starting from 0). |
| **Mask Output Dir** | The directory to store created masks, albedo, grayscale and composite images. |
| **Cropped images?** | If the generation of cropped albedo, grayscale and composite images is desired, tick the corresponding boxes. |
| **Light Source Type** | VIS, NIR or VISNIR (select depending on data needed to process). The drop-down lists for *start* and *end* sessions will update when this selection is altered. |
| **Albedo Threshold** | Values below the threshold will be removed from the mask. |
| **Minimum blob area** | Specifies the minimum number of pixels of regions to retain, which is used to remove small particles. |
| **Filter size for joining regions** | Defines the disk size that will search to join the disconnected regions. The higher the value the larger the distance. |
| **Preview Selected session and plant** | Select the session and the plant number and click ‘Preview’ to see the effect of the selected parameters in a separate window |

The expected masks and cropped images for the test data set are in directory ‘PS-Plant test data set\4. Masks and cropped albedo_grayscale_composite’

4.4.1 Generating rosette results

The GenerateResultsGUI software package generates a comma separated values file (.csv) containing 2D and 3D trait data from the plants at the rosette and leaf levels. The parameters generate by this GUI are outlined in Table 4.

**Table 4. Trait data for rosette and leaves generated using the GenerateResultsGUI.**

| **Rosette** | **Leaf** |
| --- | --- |
| 2D area | Blade 2D area |
| 3D area | Blade 3D area |
| Perimeter | Mean blade elevation |
| Circularity | Median blade elevation |
| Compactness | Point-based elevation |
| Diameter | Blade length (2D) |
| Mean elevation | Blade width (2D) |
|  | Petiole length (2D) |
|  | Petiole width (2D) |
|  | Mean petiole elevation |
|  | Median petiole elevation |
|  | Petiole start coordinates |
|  | Blade intersection coordinates |
|  | Blade tip coordinates |
|  | Leaf centroid coordinates |

There are two modes of operation for GenerateResultsGUI that are automatically detected depending on the type of mask provided. A binary mask of the rosette will lead to rosette parameters being processed and saved, while a colored mask of leaf instances will produce leaf-based analysis as well as rosette. In this step we describe how to generate rosette data from the binary masks. The generation of data for individual leaves will be described in Step 4.6.1.

The GenerateResultsGUI is shown in Fig. 14. The parameters that need to be entered in this GUI are outlined in Table 5. Note that GenerateResultsGUI also enables the user to override some of the PS-Plant configurations found in the PSConfig.properties file. These include:

- Focal length.
- Sensor size.
- Camera height.

This is particularly useful to when using pots with different heights. The default camera height is set to 40 cm, which corresponds to the distance between the base of the rig and the top acrylic plate. In the test data set we used 5 cm tall pots and the camera sensor, which is used as the reference for the camera height, was positioned 0.5 cm below the acrylic top plate, so we set the height to 34.5 cm.


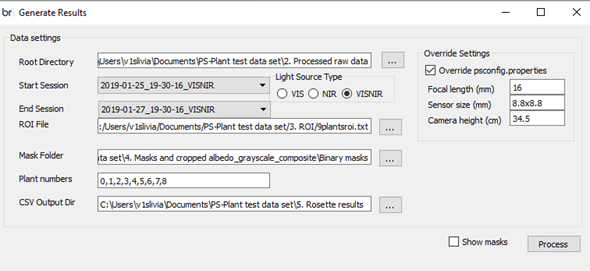


**Figure 14. Screen display of GenerateResultsGUI with parameters set to generate rosette data.**

**Table 5. Parameters of GenerateResultsGUI for generating rosette data.**

| Root Directory | The top-level data directory containing all the sessions |
| --- | --- |
| Start Session | Session to start processing from. |
| End Session | Session to stop processing with. |
| ROI File | The txt file containing the ROIs for each plant. |
| Mask Folder | The directory containing the binary masks. |
| Plant numbers | Comma separated list of plants (starting from 0). |
| CSV Output Dir | The directory where the rosette results will be saved. |
| Override settings | The software gets the default parameters from the PSConfig.properties file. If parameters need to be changed, click in this option and add in the correct parameters. |

The rosette results calculated for the test data set are in the directory ‘PS-Plant test data set\5.Rosette Results’.

4.5 Generating individual leaf masks

The software package LeafSegmentationGUI generates coloured masks with individual leaf instances. This GUI is based on the Mask R-CNN [4] instance segmentation neural network model, which can segment Arabidopsis leaves in the image using one of the three different image modalities generated by MaskGenGUI:

- Albedo.
- Grayscale.
- Composite (surface normal in x, y and albedo).

A pre-trained model ‘mask_rcnn_grayscale_testdata.h5’ is provided together with the test data set in the directory ‘PS-Plant test data set\Pre-trained Mask-RCNN model’. As stated at the beginning of the protocol, executing the software in this GUI requires a GPU with CUDA compute capability of at least 3.5 (please see <https://bit.ly/1Jnzfz8>). The LeafSegmentationGUI is shown in Fig. 15, while the parameters that need to be entered in this GUI are outlined in Table 6.


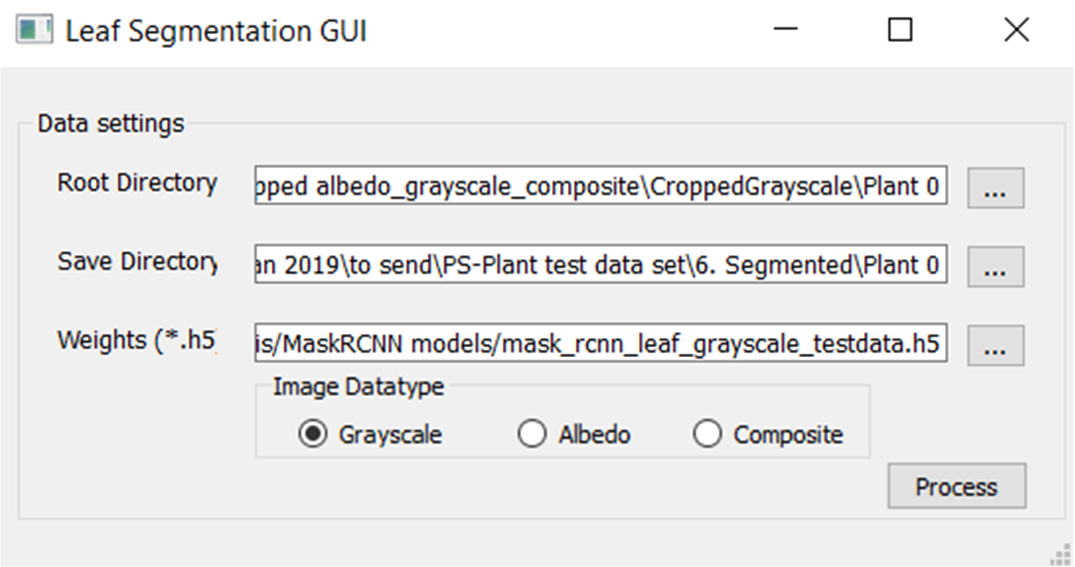


**Figure 15. Screen display of LeafSegmentationGUI with parameters set to generate leaf instances from grayscale images.**

**Table 6. Parameters for LeafSegmentationGUI.**

| **Root directory** | Directory containing the cropped images generated by MaskGenGUI. They can be albedo, grayscale or composite images. Our results showed that the Mask-RCNN performs better when using grayscale images. **Note:** this neural network model performs best with an image size of 512 x 512 pixels. |
| --- | --- |
| **Save directory** | Directory where the segmented images will be saved. |
| **Weights** | Path to the pre-trained Mask R-CNN model weights (located in ‘PS-Plant test data set\Pre-trained Mask-RCNN model\ mask_rcnn_grayscale_testdata.h5) |
| **Image datatype** | Choose the image type in the root directory. |

The leaf segmentation results calculated for the test data set are in the directory ‘PS-Plant test data set\6. Segmented’.

4.6 Leaf tracking

The LeafSegmentationGUI in step 4.5 produces individual masks for each leaf in a plant, but the leaves are labelled with random colors across the time series images. The software package LeafTrackerGUI uses a particle tracker [5] to track each individual leaf instance across a time series of images and provides the same label. The new labels for leaf instances are generated according to the four parameters of the particle tracker: ‘span’, ‘memory’, ‘max displacement’ and ‘min frames’ (Table 5). The parameters can be tuned according to each data set for optimization of the tracking process. LeafTrackerGUI also generates a ‘features.pkl’ file, which contains each leaf instance centroid location for every frame. The same .pkl file can be re-used for the generation of new labels if particle tracker parameters need to be adjusted. Re-using the features.pkl file provides faster data processing. **Note:** the generated .pkl file is only applicable to the data set from which it was generated.

The LeafTrackerGUI is shown in Fig. 16. The parameters that need to be entered in this GUI are outlined in Table 7.

*
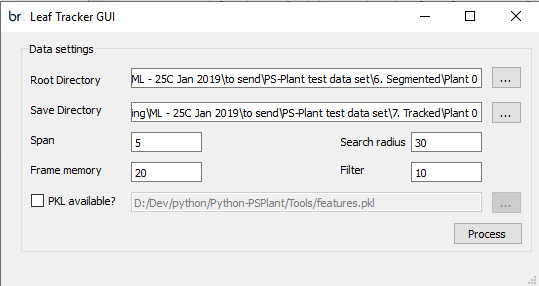
*

**Figure 16. Screen display of LeafTrackerGUI.**

**Table 7. Parameters of LeafTrackerGUI.**

| **Root directory** | The path to the directory with the masks of leaf instance segmentations. |
| --- | --- |
| **Save directory** | The path where the images with new labels are saved. |
| **Span** | Span is used to compute the velocity from [span + 1] frames. |
| **Frame memory** | The frame memory is the maximum number of frames a seen/tracked object that is absent will be remembered. |
| **Search radius** | This is the search radius, i.e., the furthest distance in pixels an object may travel between frames. |
| **Filter** | The minimum number of frames wherein an object must be seen/tracked to be considered an object. |
| **PKL file available?** | Select a ‘.pkl’ file if one is available after the initial run. |

The images generated with tracked leaves for the test data set using the parameters shown in Figure 16 are provided in the directory ‘PS-Plant test data set\7. Tracked’.

4.6.1 Generating individual leaf results

The software package GenerateResultsGUI is used to generate trait data for individual leaves, as in 4.4.1. However, here the data input will be the segmented and tracked leaf masks (Fig. 17 and Table 8).


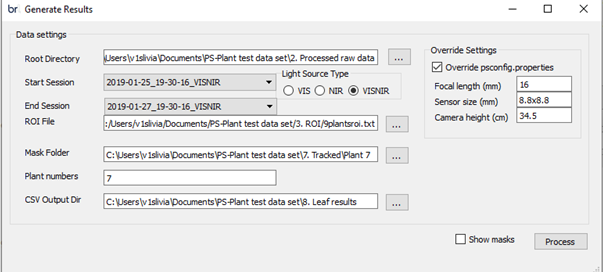


**Figure 17. Screen display of GenerateResultsGUI with parameters set to generate individual leaf data**

**Table 8. Parameters of GenerateResultsGUI for generating individual leaf data.**

| **Root Directory** | The top-level data directory containing all the sessions |
| --- | --- |
| **Start Session** | When to start processing from |
| **End Session** | When to end processing |
| **ROI File** | The txt file containing the ROIs for each plant |
| **Mask Folder** | The directory containing the segmented and tracked leaf masks |
| **Plant numbers** | Comma separated list of plants. It is preferable to do one plant at a time. |
| **CSV Output Dir** | The directory where the .csv file containing individual leaves and rosette results will be saved |
| **Override settings** | The software gets the default parameters from the PSConfig.properties file. If parameters need to be changed, click in this option and add in the correct parameters. |

When supplied with the coloured masks, the GUI will produce two .csv files: one containing the data for individual leaves and one containing the rosette data. In addition to the .csv files, the GUI will produce images of the leaf masks showing the label number assigned to each leaf. This latter is important, as it allows the user to correlate the ‘Leaf Label’ column in the .csv file with the original image (Fig. 18). These numbers are arbitrary and are not intended to correspond to the real order of the leaf numbers in the plant.


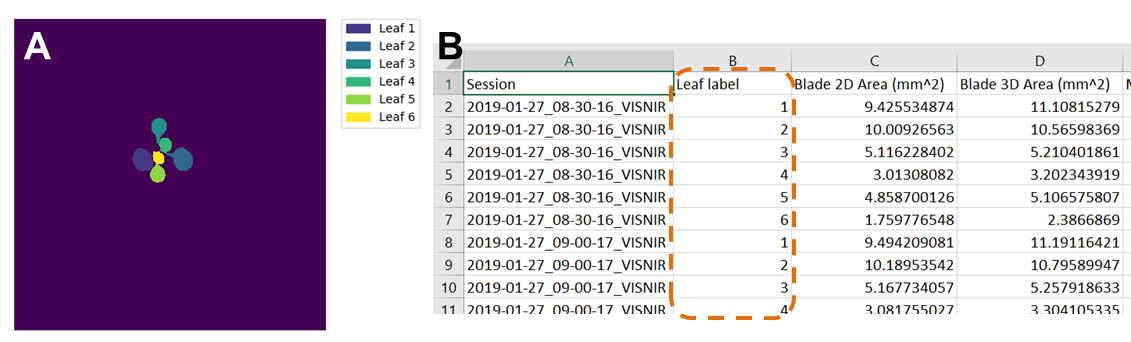


**Figure 18. Image and leaf label data produced by GenerateResultsGUI.** (A) Leaves in each image will have a number assigned (see legend). (B) The corresponding ‘Leaf Label’ column of the generated .csv file.

We have provided the individual leaf results calculated for plants 3 and 7 of the data set in the directory ‘PS-Plant test data set\8. Leaf results’ (see Table 4 for trait descriptions).

**References**

1. Qt Project. Qt: Qt Creator v.4.2.1. 2017.

2. Riverbank Computing. PyQt: PyQt v.4.11.4. 2015.

3. Frankot RT, Chellappa R. Method for Enforcing Integrability in Shape From Shading Algorithms. IEEE Trans. Pattern Anal. Mach. Intell. 1988;10:439–451.

4. He K, Gkioxari G, Dollar P, Girshick R. Mask R-CNN. Proc. IEEE Int. Conf. Comput. Vis. 2017. p. 2980–2988.

5. Allan D, Caswell T, N. K, van der Wel C. trackpy: Trackpy v0.3.2. Zenodo. 2016.
