# Supplementary material for: A photometric stereo-based 3D imaging system using computer vision and deep learning for tracking plant growth
Source: Gigascience. 2019 May 25;8(5):giz056. doi: 10.1093/gigascience/giz056 (PMC6534809; doi:10.1093/gigascience/giz056)
Supplement: giz056_Response_to_Reviewer_Comments_Original_Submission [file giz056_response_to_reviewer_comments_original_submission.pdf]

Author's Response To Reviewer Comments

Close

Dear Dr Nogoy,

The authors would like to thank the four reviewers very much for the effort and detail put into the comments and feedback. We have addressed their comments and feel that the manuscript is now much improved. In addition to the comments we have made minor corrections that we picked up during the response process. We believe the detailed experimental work in this manuscript definitely qualify this as a Research article rather than a Technical Note, and have addressed these concerns in the responses below. Where necessary, we have adjusted our conclusions.

However, we do understand that we have described a novel platform technology, and that additional details are required to encourage researchers to take up and use the PS-Plant system. Thus, we have now included i) a detailed protocol outlining the analysis workflow that includes all aspects of image analysis from capture through to dynamic growth data extraction, and more details of the learning models used (Supplementary Information S6), ii) the full software package as an open access resource on GitHub (https://bit.ly/2EFOk0O), and iii) an experimental data set to test the software available on the Edinburgh DataShare repository (https://datashare.is.ed.ac.uk/handle/10283/3279). We are happy to upload all data on Edinburgh DataShare to GigaScience’s server as required. We believe these changes have significantly increased accessibility of PS-Plant and enhance the overall impact of the manuscript, and address the associated comments raised by the reviewers. We hope that it is now suitable for publication.

We have uploaded all new components and provided the main text file with changes highlighted to compare to the previous draft. Our responses to the four reviewers are below:

Reviewer reports:

Reviewer #1: This is a novel and interesting study that utilises stereo-based 3D imaging and a low-cost and portable 3D plant phenotyping platform to track and predict the growth performance of Arabidopsis thaliana. The authors used the 'PS-Plant' 3D plant phenotyping platform to investigate rosette and individual growth analysis under a variety of conditions, and highlight a role of the daily light-dark cycle, but not the circadian oscillator, in rhythmic movements. The authors have immense research expertise in this field, the manuscript is well written, and the authors are to be commended for making the data publicly available in Edinburgh DataShare. The movies (e.g. Supplementary Data S2.mp4) are a very nice addition and are an excellent visual means of highlighting the relationship between growth / spiral phyllotaxis, and the quantitative measurements derived from the image data. In addition, an interactive model of the PS-Plant system and supporting schematics are provided. The supporting code for PS-Plant (Python-PSPlant), and additionally the supporting code for the in-house designed LED controller (Arduino) has been submitted to GigaScience.

1.1) Whereas the data is available in Edinburgh DataShare, I see inherent value in the authors additionally providing the metadata in tabular format. In this way, these metadata can be archived in the GigaScience GigaDB repository so that they are readily accessible to the research community. This will increase the findability of these data, and will allow a researcher to understand the contents of the 6.624 GB zip file of image data archived in Edinburgh DataShare prior to data download. According to the Data set description document in Edinburgh DataShare, the data set comprises 21 Arabidopsis thaliana (L. Heynh. Col-0, wild type) plants at varying time intervals (12 to 48 hours) from 11 and 24 days after germination. Consequently, I would like the authors to provide a table that unambiguously details, for each image fileset (e.g. 017-06-06_10-10-09_NIR), days after germination, and the timepoint associated with this fileset (12 to 48 hours). In addition, details of pixel resolution in regions of interest (ROIs) are currently stored in the PSConfig.properties file. I additionally invite the authors to include details of pixel resolution in this table.

We have generated the file “PS-Plant training data set – metadata.csv”, which can be found on the Edinburgh DataShare repository (https://datashare.is.ed.ac.uk/handle/10283/3280). This table contains the following details for all images in the PS-Plant data set: File name, Leaf number (total number of leaves in a given rosette), Growth temperature, Growth light intensity, Interval between imaging, Days after germination, region of interest coordinates, image resolution and image size. We have referenced this table in Supplementary Information S5, which has been renamed “PS-Plant training data set description”.

Reviewer #2: Overall, the paper is well written and carefully outlines the case for 3D imaging methods.

However, the paper draws a few conclusions which need to be addressed:

2.1) Crop Yield
The justification of work in Arabidopsis via crop improvement is a bit oversold. While the points made regarding crop yield are true, the paper does not directly focus on yield of any sort. Further, the paper does not attempt to image bio-mass accumulation in a row-crop but rather focuses on bio-mass in Arabidopsis. . To justify the application a row-crop, the authors would need to, for example, demonstrate that the segmentation techniques are not species specific.

We agree that yields and crop plants are not a focal point. As such, we have modified the text to remove excessive reference to crops and yield where appropriate:
Line 31 - Sentence modified to read “Tracking and predicting the growth performance of plants in different environments is critical for predicting the impact of global climate change”
Line 58 – Opening sentence deleted.
Line 58 - Sentence modified to read “Quantitative and accurate methods are required to aid strategies for predicting plant growth performances in our changeable natural environments.”
Line 129 - Sentence modified to read “…relationship between leaf area, biomass and yield.…”

2.2) Curvature
The authors do not carefully address the curvature of a leaf. While they do make the case for a measuring the surface normal, they do not address which curvature they are referring to for measurement. As a 2D surface embedded in 3D has two principal curvature values relating to the angle change in the surface normal with respect the change in direction along the surface, the authors need to define what they mean by curvature. One of the articles they cite, The tarani mutation alters surface curvature in 703 Arabidopsis leaves by perturbing the patterns of surface expansion and cell division, discusses these curvature differences. Other texts in the area of differential geometry also delineate these two important measures.

We agree that the measurement of surface curvature using photometric stereo requires further clarification. We have adjusted lines 215-218 in the main text to include “ as described in Supplementary Information S1” and have added a detailed sub-section titled ‘Surface curvature’ in Supplementary Information S1, which details our methodology.

2.3) Cell expansion vs division
The authors claim that under a shift in differential temperature, an increase in biomass concludes an increase in cell expansion and excludes division. Unless the authors have a paper which demonstrates this fact, it would be best to refrain from such a specific conclusion. Kinematic is the area of study which directly measures cell expansion at a tissue level without respect to the forces which drive expansion. The paper under review does not have the necessary resolution conclude or exclude at a cellular level.

We agree with the reviewer’s suggestion and have identified an appropriate paper to support our conclusion (Vile et al., 2011; PCE). We have adjusted the text from line 267 accordingly:
“Notably, in ML plants a shift from 17°C to 22°C led to an increase in biomass, while a shift from 22°C to 27°C did not. Although we have not measured leaf thickness, previous work has shown that plants grown in high temperature tend to have thinner leaves and a higher specific leaf area (the ratio of leaf area to dry mass) [Vile et al., 2011; Crawford et al 2012], which could explain the increase in area from 22°C to 27°C but no increase in biomass.”

2.4) Carbon allocation
The author show that petiole differences between high-light and low-light can be obtained with their platform via the compactness measure. They follow up this line of reasoning with the idea that "…carbon

resources are more readily allocated to leaf expansion rather than petiole growth…" (lines 270-276). The statement excludes other reasons for increase in biomass, including the roles of cell division, light quality or quantity, cell wall, and total production of "carbon resources" as control mechanism. In short, the conclusion is specific without supporting experiments. While it might be possible that the authors were intending to convey the simple idea that increase in area/mass implies that there must be a carbon increase stands to reason without their word choice.

In this particular paragraph we do not make assumptions about plant biomass, but rather focus on leaf blade area expansion and petiole elongation. However, we do agree that suggesting changes in morphology are due to differential use of carbon resources is too specific a conclusion without additional supporting evidence. Thus, we have modified the sentence on line 290 accordingly:
"This indicates that plants tend to invest more in leaf expansion compared to petiole elongation under higher light intensities."

2.5) Word choices
Improved: line 269 : improved implies more is good. Increased is a better word choice as it does not imply better but rather different only.

We agree and have changed to "increased", and modified the sentence line 283 to read:
"…growth rates were not increased by higher temperatures."

2.6) Relative Expansion Rate (RER) is also a term used in the field of kinematics. Authors might consider a different term.
The term "relative expansion rate" or "relative growth rate" have been used in several key publications in the field of plant phenotyping with the same meaning (increase of area per unit of area per unit of time). We highlight the following examples: De Vylder et al., 2012 Plant Physiol; Dhondt et al., 2014 Plant J; Apelt et al., 2015 Plant J; 2017 Plant Physiol; Minervini et al., 2017 Plant J; Dobrescu et al., 2017 Plant Methods. Therefore, we would prefer to retain "RER" as we believe readers would be more familiar with this term.

2.7) Overall, the research would stand on its own as needed phenotyping platform without the drawn conclusions. If the authors would like to draw specific conclusions, it might be best to measure some of the mutants which lead to these results.
Our key narrative is that environmental conditions have a significant impact on plant growth phenotypes. To demonstrate this, we generated a wide variety of different phenotypes specifically in a wild-type background (rather than mutant lines) using different combinations of light and temperature, and validated the capacity of PS-Plant to measure traits under these different growth conditions. Our findings highlight the dynamic relationship between light and temperature in a novel way; to enrich the discussion we have suggested several physiological mechanisms that can account for the observed growth phenotypes. We do agree with the reviewer regarding overly specific conclusions, thus to avoid misperception that additional experimental evidence is not required to complement image and growth analyses, we have added on line 307:
"Further studies on carbon allocation and starch turnover should be carried to complement these observations and hypotheses generated using PS-Plant data."

Reviewer #3: Manuscript titled "A photometric stereo-based 3D imaging system using computer vision and deep learning for tracking plant growth" presents a cost-effective photometric stereo imaging system that can image the growth performance of plants in different conditions, as well as using PS-Plant software to automate the image analysis to produce convincing quantitative growth trait measurements to assess plant growth. This work overcomes problems in 2D pixel-based computer vision and machine learning solutions designed for plant phenotypic analysis.

The results presented in the manuscript on Arabidopsis throughout the day-night cycle, different temperatures and lighting conditions are convincing. In particular, the cost-effective PS approach to investigate Arabidopsis growth architecture and growth phenotypes is highly useful for the plant research community, which has the potential to equip researchers with a powerful phenotyping tool that can soundly provide robust and novel phenotypic analysis (e.g. leaf tracking). The imaging system is well-constructed and, based on the results presented in the MS, the analysis software is also reliable.

3.1) I am a bit disappointed that the MS is fairly weak on the computer vision and deep learning parts. For example, hardware construction for image collection, the analysis workflow of PS-Plant, how the data has been constructed for training and testing, the architecture of the learning models, and the

performance of the software (e.g. computational complexity and algorithm/software related profiling). These are important items for the community to learn from PS-Plant so that other plant research groups could utilise the software and hardware presented in the MS for similar experiments, e.g. dynamic growth phenotyping and 3D phenotypic analysis.

As a methodology paper focuses on introducing new imaging device and software analytics packages (as suggested from the title of the MS), the MS might need to strengthen method sections such as imaging hardware, imaging data calibration (with different LED lighting conditions), data management, software algorithms design and possibly implementation. So the work can be reproduced. If this MS aims to discuss biological discoveries through PS-Plant, then more biological replicates, material, experimental design, etc will need to be included.

We agree with the reviewer and feel more details are required to encourage uptake of PS-Plant in the research community. Thus, we have deposited all software on a dedicated GitHub site for open source access (https://bit.ly/2EFOk0O) and have generated a detailed protocol with an accompanying video file (Supplementary Information S6) to guide users through software installation and the analysis workflow, including all aspects of image analysis from capture through to data extraction. We do state the architecture of the learning models used in the text (from line 346) as the Mask R-CNN and RNN (End-to-end instance segmentation with recurrent attention) and outline the training process (lines 351-366), but have now included the training procedure in the Table 1 legend (line 383):

"The training procedure for the RNN architecture was the same as proposed by the authors [57], while the Mask R-CNN was as follows: head layers for 10 epochs at 〚10〛^(-2) Learning Rate (LR); all layers for 30 epochs at 〚10〛^(-2) LR, 30 epochs at 〚10〛^(-3) LR, 30 epochs at 〚10〛^(-4) LR, and head layers for 10 epochs at 〚10〛^(-4) LR."

Furthermore, we have provided a test data set for testing the trained model (now available in the Edinburgh DataShare repository) as outlined in "AVAILABILITY OF SUPPORTING DATA" section (line 525). We are confident that these additions address the reviewer's concerns regarding reproduction.

Regarding biological replicates, we have stated the number of replicates used in each experiment. These are in line with published research data (e.g. De Vylder et al., 2012; Minervini et al., 2017 Plant J; Dobrescu et al., 2017 Plant Methods).

3.2) Some detailed comments are:

Line 66-68: missing citations, also add a caveat as these challenges are different for in-field plant phenotyping.

We have added additional citations for automated ground vehicles (AGVs) (Ruckelshausen et al., 2009; Underwood et al., 2017), satellite (Tattaris et al., 2016), drone (Sankaran et al., 2015) and gantry-style platform imaging of field plants (Virlet et al., 2017), and automated phenotyping of greenhouse (Tester et al., 2016, da Costa et al., 2018). We have also added on line 65: "(the challenges are different for field and indoor phenotyping)".

Line 118: define "very high resolution"

We have removed 'very' and changed this to "… high resolutions (4.1 megapixel (MP)),".

Line127: there are recent tools that count leaves based on 2D images such as rosettR, Leaf-GP, PANorama, etc.

We thank the reviewer for highlighting these and have now included additional references for Rosette Tracker (De Vylder et al., 2012), rosettR (Tomé et al., 2017) and Leaf-GP (Zhou et al., 2017).

Line 139-143: maybe list all the 2D/3D phenotypes that PS-Plant can measure?

We have added a table outlining all rosette and leaf traits that PS-Plant can generate in Supplementary Information S6 (Table 4).

Line 159: where can we download and test the software?

As above, the software is now available at https://bit.ly/2EFOk0O.

Line 172-174: it is not clear how the 3D surface and the inclination angles were calculated?

These details are provided in Supplementary Information S1, S3 and S4. We feel that we have referenced these appropriately in the text.

Line 213: how was the PRA computed, through PS-Plant or Phenotiki?

The projected rosette area PRA was derived from our PS-Plant software from surface normal data (this is not possible with 2D image analysis software, such as Phenotiki).

Line 248: what are the sample size and biological replicates?

We have double checked our figure legends – samples sizes are provided for all experimental data.

Line 277: How was RER calculated?

We have now provided the equation for this in Supporting Information S3.

Line 310-318: Do different lighting conditions impact the photometric stereo imaging?

Not in our experience. The NIR filter used allows for uniform measurements under dark and different light intensity conditions. We tested three different light intensities in the current study – we observed no impact on image acquisition between light and dark cycles. Under very high light conditions, it may be necessary to adjust the exposure time of the camera/aperture size. We have outlined how to do this in the added protocol supplement (Supplementary Information S6).

Line 337-340: what are the learning architectures? Report training and validation accuracy.

We have addressed this request in response to reviewer 3's query (3.1).

Line 363-364: "Abbreviations: SBD, Symmetric best dice; FBD foreground-background dice." is not a sentence.

We apologise for any confusion here – those lines are a caption for Table 1. We have now moved the whole caption to the Table 1 legend (lines 380-388) and included the abbreviation definitions.

Line 444-445: Has any other plant species (e.g. taller or bigger plants) been tested using PS-Plant?

No, not in its current form. Presently, the PS-Plant form factor (i.e. rig dimensions) is best suited for rosette type plants (although appropriate training data would be required to optimise segmentation in species different to Arabidopsis – we are currently exploring this). As outlined in the conclusion (line 489), incorporation of a low-cost depth camera may help for characterising taller/bigger plants with more complex architectures. This work is ongoing.

Reviewer #4: In this study, Bernotas et al. present a powerful phenotyping tool called "PS-Plant" to track 3D plant growth and leaf movement. The authors also provide a computational solution, which is based on computer vision and deep learning, to process the PS-derived data. Overall, the study is well conducted and the manuscript is well written. I have only a few minor comments.

4.1) It would be very nice if the authors could add a few sentences to discuss about the extensibility of PS-Plant to other plants (e.g., wheat, rice and so on).

Grass plants such as wheat or rice have leaves that typically grow upwards, such that most of leaf surface would not be visible using a top-down application such as PS-Plant. However, we believe that PS-Plant could be useful in young stages of non-grass crops, such as tomato, cabbage, oilseed rape, etc. We have added a sentence in the conclusion that discusses the possibility of using PS-Plant for plants other than Arabidopsis on line 484:
"In its current design, PS-Plant is optimal for measuring growth traits in rosette-shaped plants such as Arabidopsis. However, we believe it can also be used during the seedling stage of other eudicot species (e.g. tomato, cabbage, oilseed rape) to analyse circadian rhythms by observing the rhythmic

movements of cotyledons.”

4.2) The software was written as a GUI application. This is useful in most case. However, is it possible to provide a command-line version for it so that users can run the analysis in a more automatic way?

We are aware that in some cases command-line versions are preferable than GUI-based options. However, the PS-Plant framework was designed to be a GUI-based platform to ease adoption by non-computer experts. Each of the provided GUI-based scripts is designed to be as autonomous as possible. For example, a typical GUI application will process all of the required data with the selected set of parameters. Furthermore, these parameters are automatically saved in the GUI application if the same options are preferred for subsequent experiment analysis. We have made the software open source (https://bit.ly/2EFOk0O) – if community members request new functionalities, we would be happy to assist in a collaborative way.

Close
