# Supplementary material for: A photometric stereo-based 3D imaging system using computer vision and deep learning for tracking plant growth
Source: Gigascience. 2019 May 25;8(5):giz056. doi: 10.1093/gigascience/giz056 (PMC6534809; doi:10.1093/gigascience/giz056)
Supplement: giz056_Response_to_Reviewer_Comments_Revision_1 [file giz056_response_to_reviewer_comments_revision_1.pdf]

Author's Response To Reviewer Comments

Close

Dear Dr Nogoy,

The authors would like to thank you for this good news and the reviewers for their positive feedback. We have addressed the two comments from reviewer 1 and 2 below and modified the manuscript as requested in the comments of the sent .pdf. Specifically, we have reformatted the "AVAILABILITY OF SUPPORTING DATA" section, moved the "ADDITIONAL FILES" section to the end of the document, and added a gap in the references at [89] for the GigaDB DOI. We have also added this reference to line 345 in the main text (on first reference to Supplementary Information S5). Our software has been registered on the SciCrunch.org database (SCR_017032) – this information is now in the "AVAILABILITY OF SOURCE CODE AND REQUIREMENTS" section. We have now uploaded the main text as a .docx and the figures separately as high resolution .tif files.

Best regards,
Alistair McCormick

Reviewer reports:
Reviewer #1: I am delighted that the authors have responded positively to my comments, and I can confirm that the authors have provided the metadata in tabular format as requested.

One additional point is that the authors now provide a GitHub archive that they claim "allows access to the full software package as an open access resource." However, I could not find any details of an Open Source Initiative approved license being ascribed to this archive. I just wish to alert the authors that it is a de facto requirement of GigaScience that an Open Source Initiative approved license is ascribed to these software. More details of the Open Source Initiative can be found at the following link: https://opensource.org/licenses

- Thank you for this information. We have now included a GNU GPLv3 license to the PS-Plant GitHub archive.

Reviewer #2: Well done. As a note, please inspect your calculations for curvature. In figure S4 there are locations where there exist gradients for the X and Y components of the normal. As curvature is a change in the normal vector along the surface, it seems reason that these regions have a change in the angle for the normal and therefore a higher curvature. However, these regions are not highlighted in Figure S5. For example, the top most vertical leaf has a isobar-ridge running vertically along the X and Z component (gradient running along the horizonal direction). I would expect the regions with the greatest gradient of the normal component(s) to be highlighted in Figure S5. Perhaps, however, this is a matter of both scale and color mapping. Again, well done.

- We thank the reviewer for checking this (to be specific, Fig 4 and Fig 5 in Supplementary Information S1). We have double checked the calculations and found them to be correct. The reviewer is correct to notice the change in surface normal direction for the top leaf; however, this does not appear in the curvedness image. This can be explained as the change in surface normal direction is quite gradual despite being large when compared to the leaf veins of other leaves. The investigated surface curvedness is a local parameter; thus, the designed software enables the user to define the size of this area. As specified in the figure legend (Fig. 5), a kernel size of 15 was used in our case; however, it was too small to clearly distinguish the leaf vein of the top leaf, while it was adequate for the other parts of the rosette.

Close
