# Supplementary material for: A photometric stereo-based 3D imaging system using computer vision and deep learning for tracking plant growth
Source: Gigascience. 2019 May 25;8(5):giz056. doi: 10.1093/gigascience/giz056 (PMC6534809; doi:10.1093/gigascience/giz056)
Supplement: giz056_Reviewer_1_Report_Original_Submission -- Chris Armit — 12/4/2018 Reviewed [file giz056_reviewer_1_report_original_submission.pdf]

Reviewer Report

Title: A photometric stereo-based 3D imaging system using computer vision and deep learning for tracking plant growth

Version: Original Submission **Date:** 12/4/2018

Reviewer name: Chris Armit

Reviewer Comments to Author:

This is a novel and interesting study that utilises stereo-based 3D imaging and a low-cost and portable 3D plant phenotyping platform to track and predict the growth performance of Arabidopsis thaliana. The authors used the 'PS-Plant' 3D plant phenotyping platform to investigate rosette and individual growth analysis under a variety of conditions, and highlight a role of the daily light-dark cycle, but not the circadian oscillator, in rhythmic movements. The authors have immense research expertise in this field, the manuscript is well written, and the authors are to be commended for making the data publicly available in Edinburgh DataShare. The movies (e.g. Supplementary Data S2.mp4) are a very nice addition and are an excellent visual means of highlighting the relationship between growth / spiral phyllotaxis, and the quantitative measurements derived from the image data. In addition, an interactive model of the PS-Plant system and supporting schematics are provided. The supporting code for PS-Plant (Python-PSPlant), and additionally the supporting code for the in-house designed LED controller (Arduino) has been submitted to GigaScience.
Whereas the data is available in Edinburgh DataShare, I see inherent value in the authors additionally providing the metadata in tabular format. In this way, these metadata can be archived in the GigaScience GigaDB repository so that they are readily accessible to the research community. This will increase the findability of these data, and will allow a researcher to understand the contents of the 6.624 GB zip file of image data archived in Edinburgh DataShare prior to data download. According to the Dataset description document in Edinburgh DataShare, the dataset comprises 21 Arabidopsis thaliana (L. Heynh. Col-0, wild type) plants at varying time intervals (12 to 48 hours) from 11 and 24 days after germination. Consequently, I would like the authors to provide a table that unambiguously details, for each image fileset (e.g. 017-06-06_10-10-09_NIR), days after germination, and the timepoint associated with this fileset (12 to 48 hours). In addition, details of pixel resolution in regions of interest (ROIs) are currently stored in the PSConfig.properties file. I additionally invite the authors to include details of pixel resolution in this table.

Methods

Are the methods appropriate to the aims of the study, are they well described, and are necessary controls included? Choose an item.

Conclusions

Are the conclusions adequately supported by the data shown? Choose an item.

Reporting Standards

Does the manuscript adhere to the journal's guidelines on [minimum standards of reporting?]() Choose an item.

Choose an item.

Statistics

Are you able to assess all statistics in the manuscript, including the appropriateness of statistical tests used? Choose an item.

Quality of Written English

Please indicate the quality of language in the manuscript: Choose an item.

Declaration of Competing Interests

Please complete a declaration of competing interests, considering the following questions:

- Have you in the past five years received reimbursements, fees, funding, or salary from an organisation that may in any way gain or lose financially from the publication of this manuscript, either now or in the future?
- Do you hold any stocks or shares in an organisation that may in any way gain or lose financially from the publication of this manuscript, either now or in the future?
- Do you hold or are you currently applying for any patents relating to the content of the manuscript?
- Have you received reimbursements, fees, funding, or salary from an organization that holds or has applied for patents relating to the content of the manuscript?
- Do you have any other financial competing interests?
- Do you have any non-financial competing interests in relation to this paper?

If you can answer no to all of the above, write 'I declare that I have no competing interests' below. If your reply is yes to any, please give details below.

I declare that I have no competing interests.

I agree to the open peer review policy of the journal. I understand that my name will be included on my report to the authors and, if the manuscript is accepted for publication, my named report including any attachments I upload will be posted on the website along with the authors' responses. I agree for my report to be made available under an Open Access Creative Commons CC-BY license (http://creativecommons.org/licenses/by/4.0/). I understand that any comments which I do not wish to be included in my named report can be included as confidential comments to the editors, which will not be published.

I agree to the open peer review policy of the journal

To further support our reviewers, we have joined with Publons, where you can gain additional credit to further highlight your hard work (see: https://publons.com/journal/530/gigascience). On publication of this paper, your review will be automatically added to Publons, you can then choose whether or not to claim your Publons credit. I understand this statement.

Yes Choose an item.
