# Supplementary material for: A photometric stereo-based 3D imaging system using computer vision and deep learning for tracking plant growth
Source: Gigascience. 2019 May 25;8(5):giz056. doi: 10.1093/gigascience/giz056 (PMC6534809; doi:10.1093/gigascience/giz056)
Supplement: giz056_Reviewer_2_Report_Original_Submission -- Nathan Miller — 1/18/2019 Reviewed [file giz056_reviewer_2_report_original_submission.pdf]

Reviewer Report

Title: A photometric stereo-based 3D imaging system using computer vision and deep learning for tracking plant growth

Version: Original Submission **Date:** 1/18/2019

Reviewer name: Nathan Miller

Reviewer Comments to Author:

Overall, the paper is well written and carefully outlines the case for 3D imaging methods.
However, the paper draws a few conclusions which need to be addressed:
Crop Yield
The justification of work in Arabidopsis via crop improvement is a bit oversold. While the points made regarding crop yield are true, the paper does not directly focus on yield of any sort. Further, the paper does not attempt to image bio-mass accumulation in a row-crop but rather focuses on bio-mass in Arabidopsis. . To justify the application a row-crop, the authors would need to, for example, demonstrate that the segmentation techniques are not species specific.
Curvature
The authors do not carefully address the curvature of a leaf. While they do make the case for a measuring the surface normal, they do not address which curvature they are referring to for measurement. As a 2D surface embedded in 3D has two principal curvature values relating to the angle change in the surface normal with respect the change in direction along the surface, the authors need to define what they mean by curvature. One of the articles they cite, The tarani mutation alters surface curvature in 703 Arabidopsis leaves by perturbing the patterns of surface expansion and cell division, discusses these curvature differences. Other texts in the area of differential geometry also delineate these two important measures.
Cell expansion vs division
The authors claim that under a shift in differential temperature, an increase in biomass concludes an increase in cell expansion and excludes division. Unless the authors have a paper which demonstrates this fact, it would be best to refrain from such a specific conclusion. Kinematic is the area of study which directly measures cell expansion at a tissue level without respect to the forces which drive expansion. The paper under review does not have the necessary resolution conclude or exclude at a cellular level.
Carbon allocation
The author show that petiole differences between high-light and low-light can be obtained with their platform via the compactness measure. They follow up this line of reasoning with the idea that "…carbon resources are more readily allocated to leaf expansion rather than petiole growth…" (lines 270-276). The statement excludes other reasons for increase in biomass, including the roles of cell division, light quality or quantity, cell wall, and total production of "carbon resources" as control mechanism. In short, the conclusion is specific without supporting experiments. While it might be possible that the authors were intending to convey the simple idea that increase in area/mass implies that there must be a carbon increase stands to reason without their word choice.

Word choices
Improved: line 269 : improved implies more is good. Increased is a better word choice as it does not imply better but rather different only.
Relative Expansion Rate (RER) is also a term used in the field of kinematics. Authors might consider a different term.
Overall, the research would stand on its own as needed phenotyping platform without the drawn conclusions. If the authors would like to draw specific conclusions, it might be best to measure some of the mutants which lead to these results.

Methods

Are the methods appropriate to the aims of the study, are they well described, and are necessary controls included? Choose an item.

Conclusions

Are the conclusions adequately supported by the data shown? Choose an item.

Reporting Standards

Does the manuscript adhere to the journal's guidelines on minimum standards of reporting? Choose an item.

Choose an item.

Statistics

Are you able to assess all statistics in the manuscript, including the appropriateness of statistical tests used? Choose an item.

Quality of Written English

Please indicate the quality of language in the manuscript: Choose an item.

Declaration of Competing Interests

Please complete a declaration of competing interests, considering the following questions:

- Have you in the past five years received reimbursements, fees, funding, or salary from an organisation that may in any way gain or lose financially from the publication of this manuscript, either now or in the future?
- Do you hold any stocks or shares in an organisation that may in any way gain or lose financially from the publication of this manuscript, either now or in the future?
- Do you hold or are you currently applying for any patents relating to the content of the manuscript?

- Have you received reimbursements, fees, funding, or salary from an organization that holds or has applied for patents relating to the content of the manuscript?
- Do you have any other financial competing interests?
- Do you have any non-financial competing interests in relation to this paper?

If you can answer no to all of the above, write 'I declare that I have no competing interests' below. If your reply is yes to any, please give details below.

I declare that I have no competing interests

I agree to the open peer review policy of the journal. I understand that my name will be included on my report to the authors and, if the manuscript is accepted for publication, my named report including any attachments I upload will be posted on the website along with the authors' responses. I agree for my report to be made available under an Open Access Creative Commons CC-BY license (http://creativecommons.org/licenses/by/4.0/). I understand that any comments which I do not wish to be included in my named report can be included as confidential comments to the editors, which will not be published.

I agree to the open peer review policy of the journal

To further support our reviewers, we have joined with Publons, where you can gain additional credit to further highlight your hard work (see: https://publons.com/journal/530/gigascience). On publication of this paper, your review will be automatically added to Publons, you can then choose whether or not to claim your Publons credit. I understand this statement.

Yes Choose an item.
