# Supplementary material for: A photometric stereo-based 3D imaging system using computer vision and deep learning for tracking plant growth
Source: Gigascience. 2019 May 25;8(5):giz056. doi: 10.1093/gigascience/giz056 (PMC6534809; doi:10.1093/gigascience/giz056)
Supplement: giz056_Reviewer_3_Report_Original_Submission -- Ji Zhou — 2/6/2019 Reviewed [file giz056_reviewer_3_report_original_submission.pdf]

Reviewer Report

Title: A photometric stereo-based 3D imaging system using computer vision and deep learning for tracking plant growth

Version: Original Submission **Date:** 2/6/2019

Reviewer name: Ji Zhou

Reviewer Comments to Author:

Manuscript titled "A photometric stereo-based 3D imaging system using computer vision and deep learning for tracking plant growth" presents a cost-effective photometric stereo imaging system that can image the growth performance of plants in different conditions, as well as using PS-Plant software to automate the image analysis to produce convincing quantitative growth trait measurements to assess plant growth. This work overcomes problems in 2D pixel-based computer vision and machine learning solutions designed for plant phenotypic analysis.
The results presented in the manuscript on Arabidopsis throughout the day-night cycle, different temperatures and lighting conditions are convincing. In particular, the cost-effective PS approach to investigate Arabidopsis growth architecture and growth phenotypes is highly useful for the plant research community, which has the potential to equip researchers with a powerful phenotyping tool that can soundly provide robust and novel phenotypic analysis (e.g. leaf tracking). The imaging system is well-constructed and, based on the results presented in the MS, the analysis software is also reliable.
I am a bit disappointed that the MS is fairly weak on the computer vision and deep learning parts. For example, hardware construction for image collection, the analysis workflow of PS-Plant, how the data has been constructed for training and testing, the architecture of the learning models, and the performance of the software (e.g. computational complexity and algorithm/software related profiling). These are important items for the community to learn from PS-Plant so that other plant research groups could utilise the software and hardware presented in the MS for similar experiments, e.g. dynamic growth phenotyping and 3D phenotypic analysis.
As a methodology paper focuses on introducing new imaging device and software analytics packages (as suggested from the title of the MS), the MS might need to strengthen method sections such as imaging hardware, imaging data calibration (with different LED lighting conditions), data management, software algorithms design and possibly implementation. So the work can be reproduced. If this MS aims to discuss biological discoveries through PS-Plant, then more biological replicates, material, experimental design, etc will need to be included.
Some detailed comments are:
Line 66-68: missing citations, also add a caveat as these challenges are different for in-field plant phenotyping
Line 118: define "very high resolution"
Line127: there are recent tools that count leaves based on 2D images such as rosettR, Leaf-GP, PANorama, etc.
Line 139-143: maybe list all the 2D/3D phenotypes that PS-Plant can measure?

Line 159: where can we download and test the software?
Line 172-174: it is not clear how the 3D surface and the inclination angles were calculated?
Line 213: how was the PRA computed, through PS-Plant or Phenotiki?
Line 248: what are the sample size and biological replicates?
Line 277: How was RER calculated?
Line 310-318: Do different lighting conditions impact the photometric stereo imaging?
Line 337-340: what are the learning architectures? Report training and validation accuracy.
Line 363-364: "Abbreviations: SBD, Symmetric best dice; FBD foreground-background dice." is not a sentence.
Line 444-445: Has any other plant species (e.g. taller or bigger plants) been tested using PS-Plant?

Methods

Are the methods appropriate to the aims of the study, are they well described, and are necessary controls included? Choose an item.

Conclusions

Are the conclusions adequately supported by the data shown? Choose an item.

Reporting Standards

Does the manuscript adhere to the journal's guidelines on minimum standards of reporting? Choose an item.

Choose an item.

Statistics

Are you able to assess all statistics in the manuscript, including the appropriateness of statistical tests used? Choose an item.

Quality of Written English

Please indicate the quality of language in the manuscript: Choose an item.

Declaration of Competing Interests

Please complete a declaration of competing interests, considering the following questions:

- Have you in the past five years received reimbursements, fees, funding, or salary from an organisation that may in any way gain or lose financially from the publication of this manuscript, either now or in the future?
- Do you hold any stocks or shares in an organisation that may in any way gain or lose financially from the publication of this manuscript, either now or in the future?

- Do you hold or are you currently applying for any patents relating to the content of the manuscript?
- Have you received reimbursements, fees, funding, or salary from an organization that holds or has applied for patents relating to the content of the manuscript?
- Do you have any other financial competing interests?
- Do you have any non-financial competing interests in relation to this paper?

If you can answer no to all of the above, write 'I declare that I have no competing interests' below. If your reply is yes to any, please give details below.

I declare that I have no competing interests

I agree to the open peer review policy of the journal. I understand that my name will be included on my report to the authors and, if the manuscript is accepted for publication, my named report including any attachments I upload will be posted on the website along with the authors' responses. I agree for my report to be made available under an Open Access Creative Commons CC-BY license (http://creativecommons.org/licenses/by/4.0/). I understand that any comments which I do not wish to be included in my named report can be included as confidential comments to the editors, which will not be published.

I agree to the open peer review policy of the journal

To further support our reviewers, we have joined with Publons, where you can gain additional credit to further highlight your hard work (see: https://publons.com/journal/530/gigascience). On publication of this paper, your review will be automatically added to Publons, you can then choose whether or not to claim your Publons credit. I understand this statement.

Yes Choose an item.
